# Local-electrostatics-induced oxygen octahedral distortion in perovskite oxides and insight into the structure of Ruddlesden–Popper phases

**DOI:** 10.1038/s41467-021-25889-6

**Published:** 2021-09-20

**Authors:** Youngjae Hong, Pilgyu Byeon, Jumi Bak, Yoon Heo, Hye-Sung Kim, Hyung Bin Bae, Sung-Yoon Chung

**Affiliations:** 1 Department of Materials Science and Engineering and KAIST Institute for the Nanocentury, Korea Advanced Institute of Science and Technology, Yuseong-gu, Daejeon 34141 Korea; 2 KAIST Analysis Center, Korea Advanced Institute of Science and Technology, Yuseong-gu, Daejeon 34141 Korea

**Keywords:** Inorganic chemistry, Chemical physics, Materials for energy and catalysis, Structure of solids and liquids

## Abstract

As the physical properties of ABX_3_ perovskite-based oxides strongly depend on the geometry of oxygen octahedra containing transition-metal cations, precise identification of the distortion, tilt, and rotation of the octahedra is an essential step toward understanding the structure–property correlation. Here we discover an important electrostatic origin responsible for remarkable Jahn–Teller-type tetragonal distortion of oxygen octahedra during atomic-level direct observation of two-dimensional [AX] interleaved shear faults in five different perovskite-type materials, SrTiO_3_, BaCeO_3_, LaCoO_3_, LaNiO_3_, and CsPbBr_3_. When the [AX] sublayer has a net charge, for example [LaO]^+^ in LaCoO_3_ and LaNiO_3_, substantial tetragonal elongation of oxygen octahedra at the fault plane is observed and this screens the strong repulsion between the consecutive [LaO]^+^ layers. Moreover, our findings on the distortion induced by local charge are identified to be a general structural feature in lanthanide-based A_*n* + 1_B_*n*_X_3*n* + 1_-type Ruddlesden–Popper (RP) oxides with charged [LnO]^+^ (Ln = La, Pr, Nd, Eu, and Gd) sublayers, among more than 80 RP oxides and halides with high symmetry. The present study thus demonstrates that the local uneven electrostatics is a crucial factor significantly affecting the crystal structure of complex oxides.

## Abstract (web summary)

The authors observe two-dimensional shear faults in five different perovskite-type materials and detailed structures of more than 80 oxides and halides to elucidate an electrostatic origin for Jahn–Teller-type tetragonal distortion of oxygen octahedra.

## Introduction

The crystal structure of ABO_3_-type perovskites is based on the geometric stability of packing for ions of different sizes and valence states. While the relatively large cation A is coordinated with 12 oxygen anions at the dodecahedral sites, the smaller cation B is located in the oxygen octahedra connected to each other by corner-sharing, agreeing well with Pauling’s first rule for ionic compounds^1^. In particular, the distortion, including tilt and rotation, of oxygen octahedra is known to be a crucial factor governing the dielectric, magnetic, optical, and catalytic properties of perovskites^2, 3^. As a consequence, the direct identification and description of the geometry of oxygen octahedra in perovskites and derivative oxides have been key issues over the last decade^4–10^.

In addition to the relative ionic size and the cation–anion bond strength, a significant electronic contribution to the structural distortion in oxides has been noted as the Jahn–Teller effect^11, 12^. The central argument of this effect is that the energy of *d*-orbital electrons of transition-metal ions can be significantly lowered by spontaneous tetragonal elongation in such a way as to remove the degeneracy of the *d*-orbital levels. As is already known very well, high-spin Mn^3+^ (3*d*^4^) and low-spin Cu^2+^ (3*d*^9^) are typical Jahn–Teller active cations, demonstrating that the splitting of the degenerate *d*_*x*2−*y*2_ and *d*_*z*2_ levels of the *e*_*g*_ subshells and subsequent energy stabilization of electrons is achieved via substantial tetragonal distortion of oxygen octahedra. A key message of the Jahn–Teller effect is that the electronic structure of transition-metal ions should be taken into account to fully understand the crystal (or molecular) structure.

Recently, intriguing local distortion of oxygen octahedra was reported in heteroepitaxial LaNiO_3_ thin films^13^. Even though this nickelate has a pseudocubic structure, tetragonal elongation of {NiO_6_} octahedra was identified to take place exclusively at the planes of Ruddlesden–Popper (RP) two-dimensional shear faults. Taking into account that the shear faults, consisting of consecutive [LaO]^+^ layers, have electrostatically repulsive instability, the protrusion of negatively charged oxygen anions toward the shear plane was reasonably claimed to be energetically favorable for alleviating the strong repulsion between the [LaO]^+^ layers. Although this previous study was confined to a single material, LaNiO_3_, provided a physically sound explanation, further extensive work remains necessary in order to clarify and generalize the local-charge hypothesis as another important electrostatic origin responsible for the remarkable tetragonal distortion of oxygen octahedra in perovskite-derivative oxides.

To this end, in this work, we extended our atomic-scale direct observations of two-dimensional shear faults^14–18^ into five different perovskite-type materials, SrTiO_3_, BaCeO_3_, LaCoO_3_, LaNiO_3_, and CsPbBr_3_. First, we observed substantial tetragonal distortion of {BO_6_} octahedra at the fault plane, when an interleaved [AO]–[AO] shear fault has a local charge (for example, [LaO]^+^ in LaCoO_3_ and LaNiO_3_). In stark contrast, no significant structural distortion was probed in the {BO_6_} octahedra when the [AO] sublayer has no effective charge ([SrO] in SrTiO_3_, [BaO] in BaCeO_3_, and [CsBr] in CsPbBr_3_). Furthermore, by examining a series of 87 A_*n* + 1_B_*n*_X_3*n* + 1_-type Ruddlesden–Popper (RP) oxides and halides with high symmetry, we found that this local-charge-induced tetragonal elongation of oxygen octahedra is a striking common feature observed in most of the RP phases having the charged [AO] layer. Consequently, our findings on the electrostatic origin of the distortion appear to be a general phenomenon in oxides containing multiple cations.

## Results and discussion

### Observation of shear faults

**Fig. 1:**
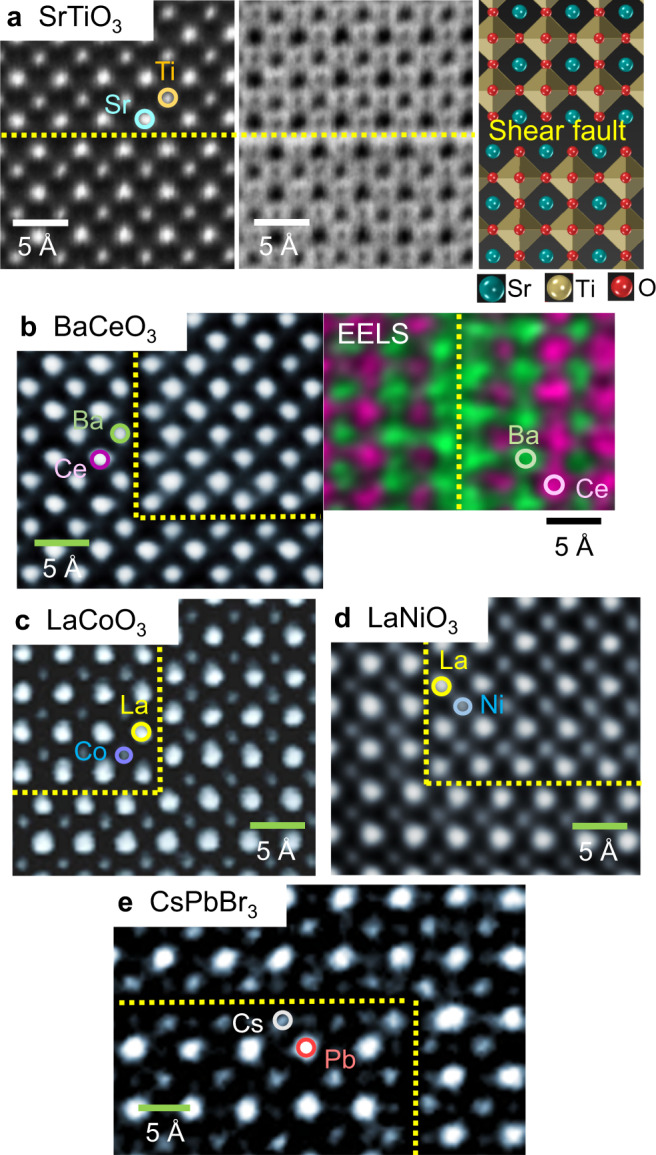
STEM images for the formation of homologous shear faults. Each image was acquired in **a** SrTiO_3_, **b** BaCeO_3_, **c** LaCoO_3_, **d** LaNiO_3_, and **e** CsPbBr_3_. The yellow broken lines in each image indicate the fault planes consisting of two consecutive [AX] sublayers, as schematically described in **a** together with an annular bright-field image. The atomic-scale chemical map shown in **b** was obtained by using EELS to verify the consecutive [BaO] sublayers.

For fault observations, we prepared polycrystalline dense SrTiO_3_, BaCeO_3_, and LaCoO_3_ sintered pellets, epitaxial LaNiO_3_ thin films, and CsPbBr_3_ nanocrystals (see the “Methods” section), all of which contain homologous stacking faults represented by two consecutive rocksalt [AX] layers (A = Sr, Ba, La, Cs; X = O, Br). Figure 1 shows a series of high-angle annular dark-field (HAADF) scanning transmission electron microscopy (STEM)^19–21^ images at atomic resolution, verifying the presence of rocksalt [AX] faults in all five perovskite crystals (see Supplementary Fig. 1 for lower magnification images). As the atomic number, *Z*, of Ba (*Z*_Ba_ = 56) and Ce (*Z*_Ce_ = 58) is very similar, individual columns are not readily discriminated in BaCeO_3_, although the image contrast of the faults is sufficiently distinguishable at a lower magnification (Supplementary Fig. 1b). A chemical map obtained by electron energy-loss spectroscopy (EELS) is thus provided in Fig. 1b to confirm the [BaO]–[BaO] layers at the fault. In addition, note that the *Z* value of Cs (*Z*_Cs_ = 55) is much lower than that of Pb (*Z*_Pb_ = 82) in CsPbBr_3_. (See Supplementary Fig. 2 for additional STEM images of CsPbBr_3_ nanocrystals). Consequently, the column intensity at the [CsBr] faults in a HAADF image should be distinctively lower, as shown in Fig. 1e, in contrast to the image feature at the faults observed in SrTiO_3_, LaCoO_3_, and LaNiO_3_.

### Oxygen displacement and local electrostatics

**Fig. 2:**
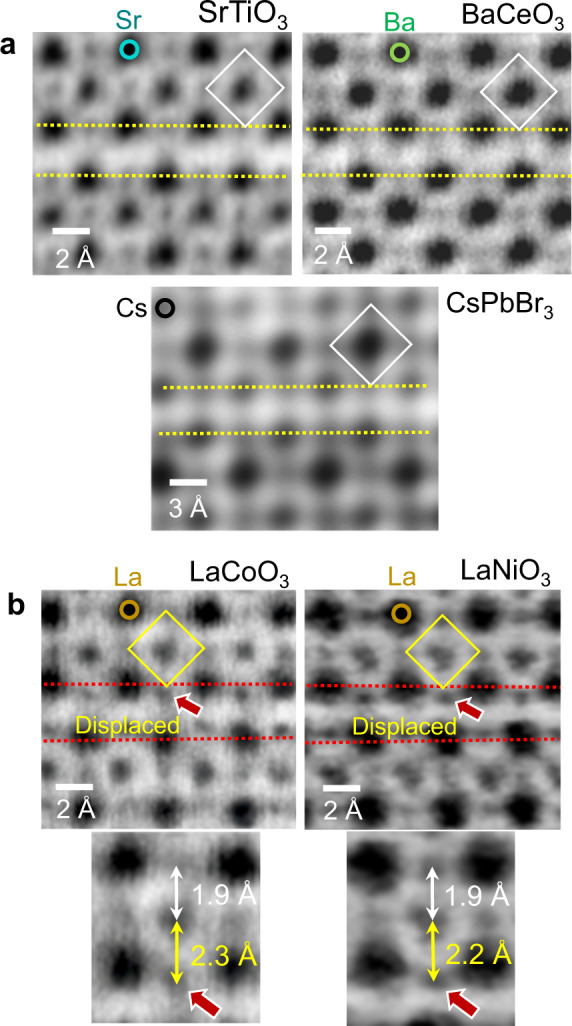
ABF-STEM images of the shear faults to visualize the position of anions. **a** As denoted by a white square diamond in each image, no distortion of anion octahedra is observed at the faults (yellow broken lines) in SrTiO_3_, BaCeO_3_, and CsPbBr_3_. **b** In contrast, as indicated by a red arrow in each image, substantial displacement of oxygen anions at the fault planes should be noted in LaCoO_3_ and LaNiO_3_, showing the position deviation from the yellow square diamonds. Magnified images directly provide quantitative information on the elongation of bond lengths in Co–O (2.3 Å) and Ni–O (2.2 Å).

To visualize the position of light-element anions, we utilized the annular bright-field (ABF) mode in STEM^22–29^. All the ABF images acquired from the fault region in the five perovskites are provided in Fig. 2 (see Supplementary Fig. 3 for pairs of ABF and HAADF images of the bulk crystals as references). A readily recognizable structural feature from this set of atomic-scale observations is that oxygen octahedra at the faults are asymmetrically elongated along the *z* axis in LaCoO_3_ and LaNiO_3_ (Fig. 2b), while no distortion of anion octahedra is identified in the other three perovskites, SrTiO_3_, BaCeO_3_, and CsPbBr_3_, as denoted by a white square diamond in each image in Fig. 2a. In particular, more than a 20% increment in the Co–O length from 1.9 to 2.3 Å in LaCoO_3_ and a 15% increment in the Ni–O length from 1.9 to 2.2 Å in LaNiO_3_ are presented in the magnified images in Fig. 2b.

**Fig. 3:**
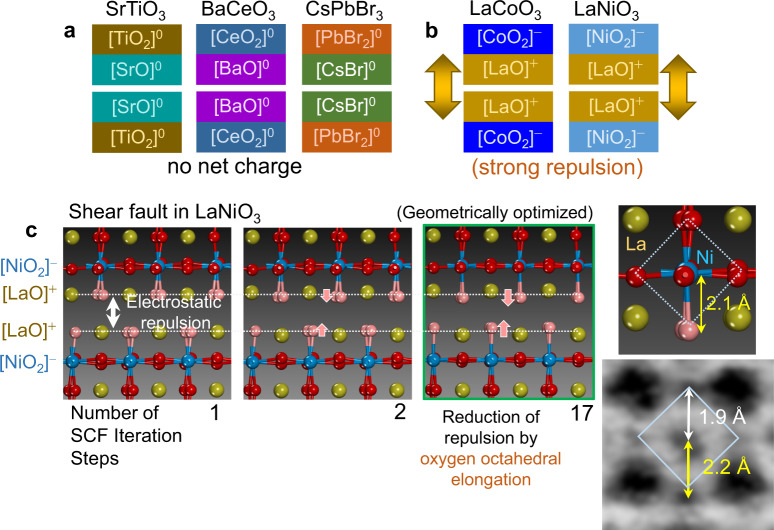
Description of sublayer polarity in perovskites and geometry optimization of atom configuration during DFT calculation. The effective charges of [AX] and [BX_2_] sublayers in the five perovskites are indicated. **a** No polarity of sublayers is present in SrTiO_3_, BaCeO_3_, and CsPbBr_3_. **b** In contrast, [LaO]^+^ in LaCoO_3_ and LaNiO_3_ is noted to contain strong electrostatic repulsion at the fault. **c** Geometry optimization with SCF steps at the fault in LaNiO_3_ is achieved by the oxygen displacements toward the fault plane, as denoted by small arrows in the illustrations. Good agreement of the Ni–O elongation obtained by the DFT calculation and the direct ABF-STEM observation is verified.

These two perovskite groups revealing distinct octahedral aspects have their own local electrostatic characteristics from the viewpoint of a layer structure. As schematically depicted in Fig. 3a, no effective local charge is noted in each of the [AX] and [BX_2_] sublayers in three perovskites, SrTiO_3_, BaCeO_3_, and CsPbBr_3_. For example, because the valence states of Sr, Ti, and O in SrTiO_3_ are 2+, 4+, and 2−, respectively, the sublayers, [SrO]^0^ and [TiO_2_]^0^, have no net charges^30, 31^. Therefore, no electrostatic perturbation is induced at the homologous [SrO]–[SrO] fault plane, indicating that the (pseudo)cubic bulk structure can be preserved at the fault region without showing elongation of oxygen octahedra. In the same manner, there is no local charge at the faults in BaCeO_3_ ([BaO]^0^) and CsPbBr_3_ ([CsBr]^0^).

In contrast, the La-based perovskite oxides, LaCoO_3_ and LaNiO_3_, contain local net charge at each of the sublayers, as described in Fig. 3b. As the valence states of La, Co (and Ni), and O are 3+, 3+, and 2−, respectively, the bulk structure of LaCoO_3_ (and LaNiO_3_) consists of positively charged [LaO]^+^ layers and negatively charged [CoO_2_]^−^ (and [NiO_2_]^−^) layers in an alternating manner along the *z* direction. Consequently, if two [LaO]^+^ layers are consecutively placed at the faults, electrostatically strong repulsion is inevitably induced, resulting in serious local instability (Fig. 3b). One of the easiest way to reduce the positive-charge repulsion between two [LaO]^+^ layers is that the negatively charged O^2−^ anions adjacent to the fault displace toward the fault plane and thereby screen the effective positive charge. Even if the displacement of oxygen anions is at an angstrom level, it appears to be fairly efficient to significantly reduce the repulsive instability at the fault plane.

### Theoretical calculations

The displacement of oxygen anions was also verified by ab initio density functional theory (DFT) calculation. Figure 3c shows the atom position variation in a LaNiO_3_ supercell containing a shear fault during the DFT calculation for geometry optimization. As shown in the supercell illustration of the first self-consistent field (SCF) iteration step, the initial configuration of oxygen atoms (light red) at the fault plane was set to be identical to the position of oxygen in the bulk. It is noted that the Ni–O bonds adjacent to the fault substantially elongate even after one SCF step, as can be seen in the second illustration, together with significant stabilization of the lattice energy (see Supplementary Fig. 4 for the plot of the lattice energy variation with SCF steps). The third illustration shows the finally optimized configuration of the supercell cell, revealing that the Ni–O bond length adjacent to the fault plane is 2.1 Å in the magnified illustration. This value after geometry optimization is in fairly good agreement with the experimentally observed Ni–O length, 2.2 Å, as directly compared in the ABF-STEM image. The DFT calculation thus confirms that elongation of the Ni–O bond length at the fault is an energetically stable configuration. At the same time, La^3+^ cations are also observed to displace along the *z* axis out of the fault plane in the series of supercell illustrations in order to facilitate the reduction of the repulsion at the fault.

We also carried out another set of DFT calculations to compare the density of states (DOS) of the Ni 3*d* and O 2*p* orbitals in LaNiO_3_ with and without a fault. As demonstrated in Supplementary Fig. 5, the overall substantial degree of overlap between the Ni 3*d* and O 2*p* states in addition to the metallic behavior with no bandgap does not significantly vary between the two DOS plots. However, as indicated by arrows, the noticeable increases of O 2*p* and Ni 3*d* states at the bottom, in the middle, and at the top of the band in the DOS plot are key changes of the electronic structure that result from the *z* axis elongation of [NiO_6_] octahedra at the fault plane. When six oxygen ligands around a metal cation are located in an octahedral symmetry, the metal 3*d* states are discreetly split as two degenerate *t*_2g_ and *e*_g_ levels. In contrast, if this symmetry is broken either by oxygen octahedral elongation or via other perturbation^27, 28, 32, 33^, the degeneracy of the two levels is removed, resulting in a wide range of energy levels of the metal 3*d* states in addition to the O 2*p* states for hybridization.

### EELS analyses

**Fig. 4:**
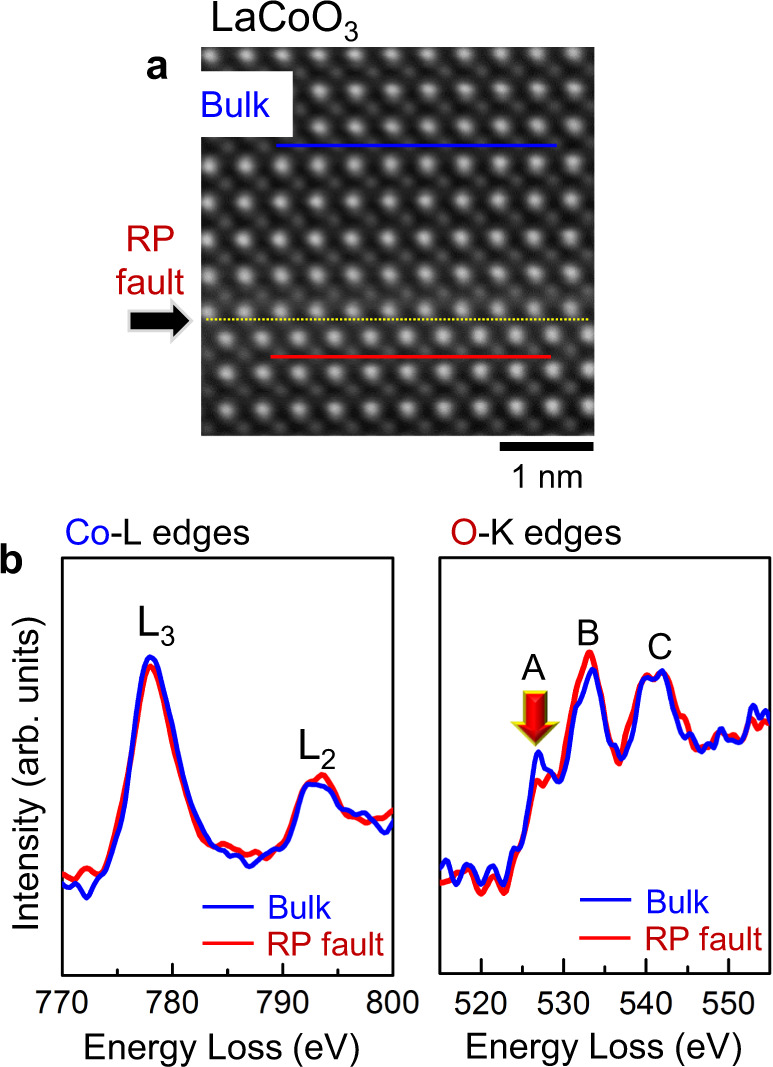
EELS analysis at the RP fault in LaCoO_3_. **a** As indicated in the image, Co-*L* and O-*K* edges were acquired from the bulk (blue line) and the RP fault (red line) in LaCoO_3_. **b** Three major peaks in the O *K*-edges represent the hybridizations of O 2*p* with Co 3*d* (A), La 5*d* (B), and Co 4*s*/4*p* (C) orbitals, respectively. No noticeable peak shift or intensity variation is observed in the Co-*L* edges, showing the invariant oxidation states of Co^3+^ at the fault plane. A red arrow in the plot of O-*K* edges indicates the intensity reduction of peak A at the fault (red curves), whereas the intensities of peaks B and C hardly vary between the bulk and the fault.

Atomic-column resolved EELS analyses on the RP shear faults were performed to examine the variation of energy-loss spectra in addition to the oxidation state of transition metals. As the Ni-L_3_ edge seriously overlaps with the La-M_4_ edge, we selected LaCoO_3_ and SrTiO_3_ samples containing a shear fault instead of LaNiO_3_ for the EELS analyses. Figure 4 shows a comparison of Co-L and O-K edges acquired from the bulk and the fault in LaCoO_3_. First, no noticeable changes of the peak intensity and position in the Co-L_2,3_ edges from the bulk and the fault were identified, directly indicating the oxidation state of Co^3+^ is invariant at the fault. Second, the three major peaks in the O-K edge are represented for the hybridizations of O 2*p* with Co 3*d* (peak A), La 5*d* (peak B) and Co 4*s*/4*p* (peak C), respectively^34–37^. The intensity of peak A obtained from the fault substantially diminishes, as denoted by a red arrow in the plot, while the intensity of the other peaks does not significantly vary between the bulk and the fault. This reduction of the pre-peak in the O-*K* edge appears to consistently correlate with the elongation of the Co–O bond length at the faults. Because the bond distance between Co and O becomes longer, the effect of oxygen ligands on Co should be reduced, and thereby the degree of hybridization of O 2*p* with Co 3*d* orbitals decreases. Consequently, as demonstrated in a perovskite cobaltate^35^ and layered lithium transition-metal oxides^38, 39^, which contain oxygen vacancies, the lower intensity of peak A likely stems from the comparatively weak orbital interaction. Indeed, such a peak reduction was not observable in the O-*K* edge obtained from the fault in SrTiO_3_, where no elongation of Ti–O bond length is identified at the faults (see Supplementary Fig. 6 for details on the EELS analysis of the fault in SrTiO_3_).

### Local structures of RP oxides

**Fig. 5:**
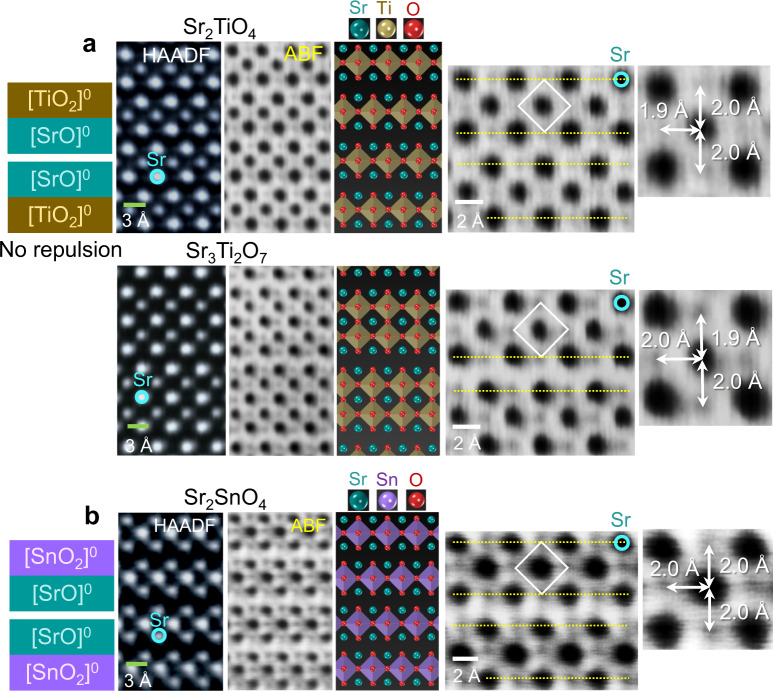
STEM images showing the structure of Sr-based RP oxides. Atomic-column-resolved images were acquired in **a** Sr_2_TiO_4_ and Sr_3_Ti_2_O_7_ and **b** Sr_2_SnO_4_, as listed in Supplementary Table 1. Because these RP oxides have no effective charge in [SrO] sublayers, no distortion of oxygen octahedra is identified in each structure. Magnified ABF images directly provide quantitative information on the Ti–O and Sn–O bond lengths.

In addition to directly demonstrating the distortion of oxygen octahedra at the faults in perovskite oxides, the findings in this work have further notable implications for the in-depth understanding of the structures of a variety of RP oxides and halides, which exhibit a very wide range of physical properties encompassing dielectric and unusual ferroic, multiferroic, electrocatalytic, and optical behavior^7–10, 40–45^. As can be easily recognized from their general formula, A_*n* + 1_B_*n*_X_3*n* + 1_ or equivalently AX[ABX_3_]_*n*_, the presence of consecutive [AX] sublayers is a common structural feature irrespective of the value of *n*. In particular, it is noted that every anion octahedron in layer-structured A_2_BX_4_ (*n* = 1) (or AX[ABX_3_]) is in contact with two shear planes, as denoted by yellow broken lines in the atomic illustration of Sr_2_TiO_4_ in Fig. 5a. If the [AX] layer has an effective charge, as aforementioned in [LaO]^+^, charge-induced electrostatic repulsion would considerably accumulate over the entire crystal lattice of A_2_BX_4_. Consequently, substantial elongation of {BX_6_} octahedra should take place to significantly reduce the repulsive instability in the consecutive [AX] layers.

We examined more than 80 RP-type oxides and halides with high symmetry from the open database of inorganic materials (the Materials Project) and the Inorganic Crystal Structure Database (ICSD) to scrutinize whether the local net charge of [AX] sublayers exerts a general influence on the tetragonal distortion of anion octahedra in RP phases. Note that most of the RP oxides and halides under our examination in this work were confined to centrosymmetric phases with a single composition at each of the A and B sites in order to exclude any other complexity originating from the polarity induced by either cation off-centering from the second-order Jahn–Teller effect^46, 47^, octahedral rotations^5–7, 10^, or cation ordering^48, 49^. Taking this into consideration, structure comparisons were made largely between RP phases with a space group, *I*4/*mmm*^9, 49^.

Supplementary Tables 1–3 list all the structural information regarding the ratios of *x* axis and *z* axis B–X bond lengths, *L*_*z*_/*L*_*x*_, in 87 RP oxides and halides (see the detailed illustration in Supplementary Table 1 for the definition of *L*_*z*_/*L*_*x*_); Ca-, Sr-, and Ba-based RP oxides with no local charge in Supplementary Table 1, K- and Cs-based RP halides in Supplementary Table S2, and lanthanide-based RP oxides in Supplementary Table 3. “2.1.4.”, “3.2.7.”, and “4.3.10.” in each table represent A_2_BX_4_ (*n* = 1), A_3_B_2_X_7_ (*n* = 2), and A_4_B_3_X_10_ (*n* = 3) RP phases, respectively. All of the 87 crystal structures are also illustrated in Supplementary Figs. 7–60. This series of tables and supplementary figures demonstrates the notable and exclusive effect of the [AX] local charge on the *z* axis elongation of octahedra (*L*_*z*_/*L*_*x*_ > 1.04) in the cases of lanthanide-based (A = La, Pr, Nd, Eu, and Gd) RP oxides. We set the criterion of 4% elongation by rounding down the minimum value, 1.048 (which corresponds to 4.8% elongation in the case of EuCoO_3_ in Supplementary Table 3), to three decimal places. As denoted by the red-brown background in Supplementary Table 3, all the Ln-based RP oxides exhibit more than 4% substantial elongation of *z* axis B–O bond lengths, showing strong tetragonal distortion of octahedra (see Supplementary Figs. 46–60 for their crystal structures).

### Direct observations in RP oxides

To experimentally visualize the local-charge-induced elongation of oxygen octahedra in addition to referencing the structural database, we carried out direct atomic-column-resolved ABF-STEM observations in a variety of RP oxides selected from Supplementary Tables 1 and 3. Figure 5 exemplifies ABF images and their enlargements of three Sr-based RP oxides, Sr_2_TiO_4_, Sr_3_Ti_2_O_7_, and Sr_2_SnO_4_ (see Supplementary Figs. 61 for low-magnification STEM images), with no local charge at the fault plane of [SrO]^0^–[SrO]^0^ consecutive layers, as listed in Supplementary Table 1. The nearly identical *x* axis and *z* axis Ti–O (and Sn–O) bond lengths (*L*_*z*_ and *L*_*x*_) in each oxide agree well with the information provided in the table, verifying the ratio *L*_*z*_/*L*_*x*_ ≈ 1.

**Fig. 6:**
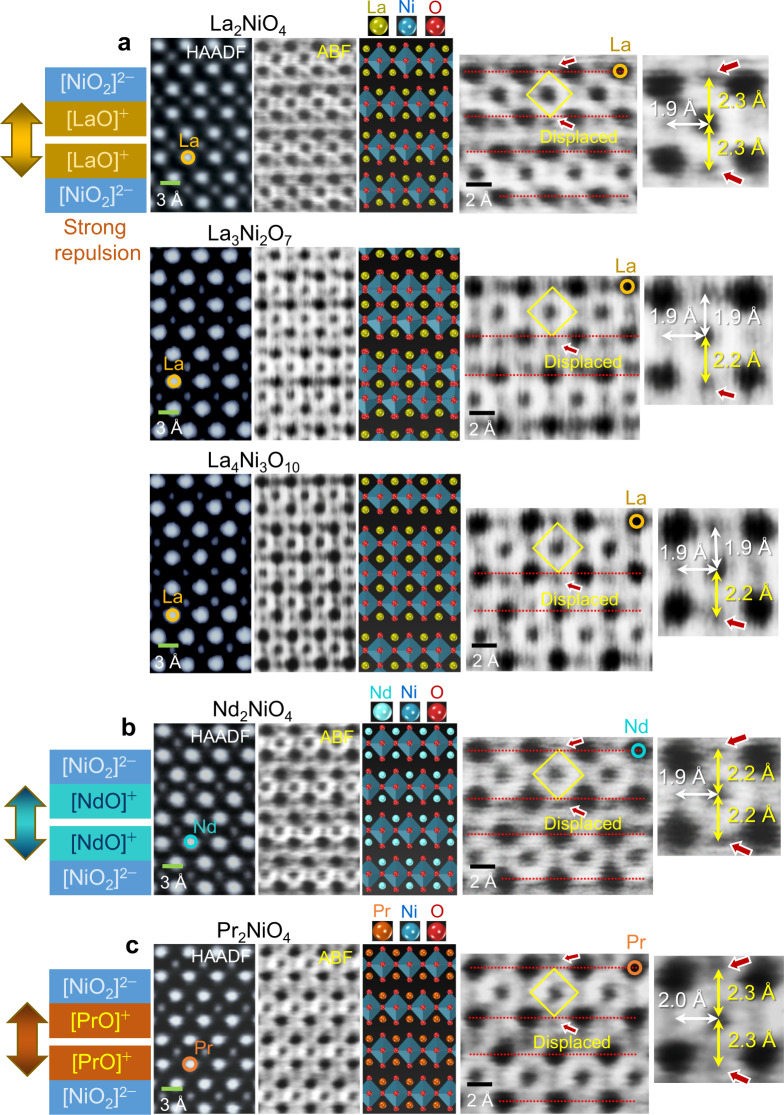
STEM images showing the structure of lanthanide-based RP nickelates. Atomic-column-resolved images were acquired in **a** La_2_NiO_4_, La_3_Ni_2_O_7_, and La_4_Ni_3_O_10_, **b** Nd_2_NiO_4_, and **c** Pr_2_NiO_4_, as listed in Supplementary Table 3. Strong local repulsion between [LnO]^+^ sublayers is illustrated in the left-hand column. Significant elongation of the Ni–O bonds at the shear planes is denoted by yellow arrows in the ABF images, providing quantitative information on the bond lengths. Red arrows in each image indicate substantial displacement of oxygen anions.

**Fig. 7:**
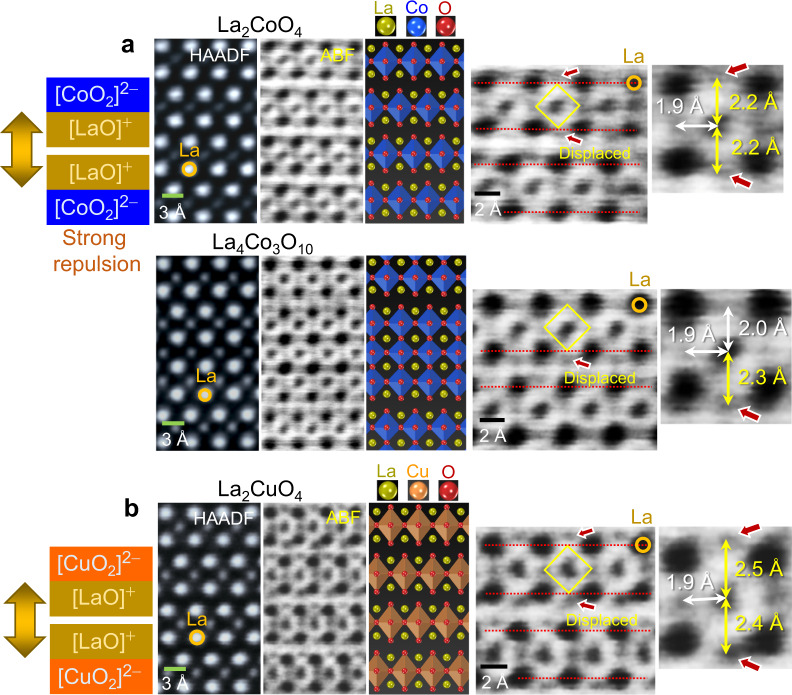
STEM images showing the structure of La-based RP oxides. Atomic-column-resolved images were acquired in **a** La_2_CoO_4_ and La_4_Co_3_O_10_ and **b** La_2_CuO_4_, as listed in Supplementary Table 3. Strong local repulsion between [LaO]^+^ sublayers is illustrated in the left-hand column. Significant elongation of the Co–O and Cu–O bonds at the shear planes is denoted by yellow arrows in the ABF images, providing quantitative information on the bond lengths. As Cu^2+^ is known as a strong Jahn–Teller cation, {CuO_6_} octahedra in La_2_CuO_4_ show more prominent *z* axis elongation. Red arrows in each image indicate substantial displacement of oxygen anions.

Figure 6 shows the actual structures and their atomic-scale ABF images of five lanthanide-based RP nickelates, La_2_NiO_4_, La_3_Ni_2_O_7_, La_4_Ni_3_O_10_, Nd_2_NiO_4_, and Pr_2_NiO_4_, selected from Supplementary Table 3, with an effective local charge of the sublayers, [LaO]^+^, [NdO]^+^, and [PrO]^+^, as schematically illustrated in the left-hand column (see Supplementary Figs. 62 for low-magnification STEM images). In particular, Fig. 6b, c specifically exemplify the tetragonal elongation of every oxygen octahedra in Nd_2_NiO_4_, and Pr_2_NiO_4_, in addition to La_2_NiO_4_ in Fig. 6a. The Ni–O bond lengths in each RP nickelate can be directly acquired from the ABF images in the right-hand column. As denoted by yellow arrows in the enlargements, the substantial elongation (more than 15%) of *z* axis Ni–O bonds toward the fault planes is readily recognized. This structural feature is consistently confirmed in La-based RP cobaltates and cuprates, as demonstrated in Fig. 7. Moreover, Cu^2+^ is a well-known strong Jahn–Teller cation. As a consequence, more prominent *z* axis elongation (>27%) of {CuO_6_} octahedra in La_2_CuO_4_ (Fig. 7b) than that of {CoO_6_} in La_2_CoO_4_ and {NiO_6_} in La_2_NiO_4_ is identified by a combination of the [LaO]^+^ local charge and the Jahn–Teller effect. To clarify that this >27% strong elongation of {CuO_6_} octahedra is a common aspect in Pr_2_CuO_4_ and Nd_2_CuO_4_ as well (Supplementary Table 3), an additional structure comparison is provided in Supplementary Fig. 63.

We summarize several representative A_2_BX_4_ (*n* = 1) oxides and fluorides among the RP phases with the space group *I*4/*mmm* in Supplementary Fig. 64 to highlight the impact of the net charge of the sublayers on the significant elongation of oxygen octahedra. As schematically described in Supplementary Fig. 64a, [SrO] in Sr_2_TiO_4_, [KF] in K_2_NiF_4_, and [CsF] in Cs_2_CuF_4_ have no effective charge. The six B–X bond lengths in each of {TiO_6_}, {NiF_6_}, and {CuF_6_} octahedra are thus nearly identical (<4% variation), showing no substantial geometric distortion. In contrast, as the [LaO]^+^ sublayers in all the La-based RP oxides are positively charged, the protrusion of oxygen anions toward the shear planes is identified and this reduces the repulsive electrostatic instability. Even though B-site cations are the same (Ni^2+^ in K_2_NiF_4_ and La_2_NiO_4_ and Cu^2+^ in Cs_2_CuF_4_ and La_2_CuO_4_, respectively), the distinct elongation of {NiO_6_} in La_2_NiO_4_ and {CuO_6_} in La_2_CuO_4_ in contrast to {NiF_6_} in K_2_NiF_4_ and {CuF_6_} in Cs_2_CuF_4_ clarifies the crucial influence of the electrostatic charge on the tetragonal distortion of oxygen octahedra in RP oxides. Similarly, because the other lanthanide cations, Ln = Pr, Nd, Gd, at the A sites in RP oxides are trivalent, the [LnO]^+^ sublayers are also positively charged, as in the case of the La-based RP oxides. As specifically exemplified in Supplementary Fig. 64c for Pr- and Nd-based cobaltates, therefore, substantial *z* axis octahedral elongation is consistently identified.

**Fig. 8:**
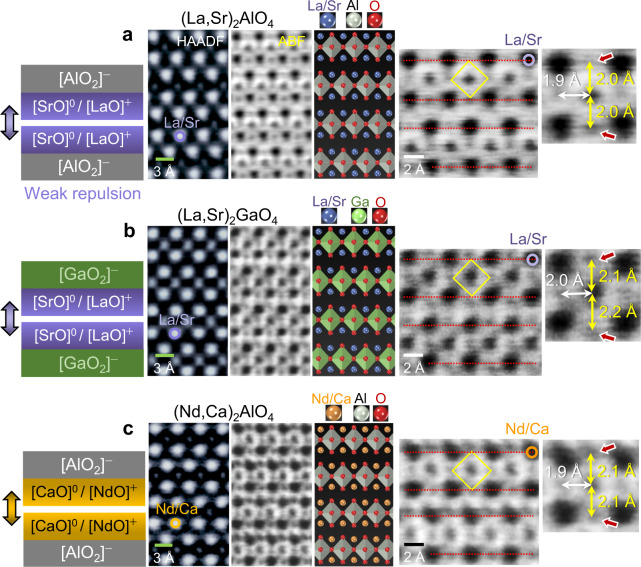
STEM images showing the structure of (A′A″)_2_BO_4_-type RP oxides. Atomic-column-resolved images were acquired in **a** (La,Sr)_2_AlO_4_, **b** (La,Sr)_2_GaO_4_, and **c** (Nd,Ca)_2_AlO_4_. Note that the A-site sublayers include [SrO]^0^ (or [CaO]^0^) with no effective charge and subsequently the electrostatic repulsion between the layers diminishes. Therefore, the elongation of the Al–O and Ga–O bonds toward the shear planes is not remarkable, showing a bond length of ≤ 2.2 Å. Magnified ABF images directly provide quantitative information on the bond lengths. Red arrows in each image indicate a slight displacement of oxygen anions.

When the structure of A-site solid solution (A′A″)_2_BO_4_, (where the valence states of A′ and A″ are 3+ and 2+, respectively) is scrutinized, the effect of electrostatic charge on the elongation of oxygen octahedra is further supported. Figure 8 shows the STEM images of (A′A″)_2_BO_4_-type (La,Sr)_2_AlO_4_, (La,Sr)_2_GaO_4_, and (Nd,Ca)_2_AlO_4_ (see Supplementary Fig. 65 for composition verification of A-site solid solutions). As schematically depicted in the illustrations in the left-hand column, the A-site sublayers of these RP oxides encompass [SrO]^0^ (or [CaO]^0^) with no local charge in addition to charged [LaO]^+^ (or [NdO]^+^) as a solid solution. Consequently, the positive-charge electrostatics between the consecutive shear layers should diminish, resulting in comparatively weak interlayer repulsion. Considerably strong elongation of oxygen octahedra for screening the effective positive charge at the fault is thus no longer necessary. Indeed, as denoted in each of the magnified ABF images in the right-hand column, the length of *z* axis Al–O and Ga–O bonds does not exceed 2.2 Å, in contrast to a value of 2.3 Å in some nickelates (Fig. 6) and cobaltates (Fig. 7a) and even 2.5 Å in cuprates (Fig. 7b). Therefore, this series of direct observations in Fig. 8 provides consistent evidence for our findings on the electrostatics-induced distortion of oxygen octahedral in RP oxides.

In conclusion, we have observed the formation of [AX]–[AX] interleaved shear faults as common planar defects in five ABX_3_ perovskite-type phases, SrTiO_3_, BaCeO_3_, LaCoO_3_, LaNiO_3_, and CsPbBr_3_. As each fault generated in LaCoO_3_ and LaNiO_3_ consists of two positively charged [LaO]^+^ sublayers in contrast to [AX] with no local charge in SrTiO_3_, BaCeO_3_, and CsPbBr_3_, strong electrostatic repulsion can be established at the fault plane. We identified that this charge-induced instability could remarkably diminish via the asymmetric tetragonal distortion of oxygen octahedra by the protrusion of oxygen anions toward the fault plane and thereby effectively screen the repulsion. Furthermore, via direct atomic-column-resolved visualization by ABF imaging in 14 representative RP oxides, our atomic-level findings regarding the tetragonal distortion of oxygen octahedra at the shear faults provide crucial insight to consistently explain the octahedron elongation commonly observed in lanthanide-based RP oxides among more than 80 RP-type oxides and halides. In addition to the electronic structure of metal cations on the basis of the Jahn–Teller effect, the present study suggests that the local electrostatic instability is a significant cause of the tetragonal distortion of oxygen octahedra in complex oxides.

## Methods

### Preparation of polycrystalline samples

To prepare polycrystalline perovskite and RP phase samples, powders were first synthesized via a conventional solid-state reaction method using SrCO_3_ (99.9%, Sigma Aldrich) and TiO_2_ (99.9%, Sigma Aldrich) for SrTiO_3_, Sr_2_TiO_4_, and Sr_3_Ti_2_O_7_; SrCO_3_ and SnO_2_ (99.9%, Sigma Aldrich) for Sr_2_SnO_4_; BaCO_3_ (99.999%, Sigma Aldrich) and CeO_2_ (99.95%, Sigma Aldrich) for BaCeO_3_; La(OH)_3_ (99.9%, Sigma Aldrich) and NiO (99.8%, Sigma Aldrich) for La_3_Ni_2_O_7_ and La_4_Ni_3_O_10_; La(OH)_3_ and Co_3_O_4_ (99.5%, Sigma Aldrich) for LaCoO_3_; La(OH)_3_ and CuO (99.99%, Sigma Aldrich) for La_2_CuO_4_; Nd_2_O_3_ (99.9%, Sigma Aldrich) and NiO for Nd_2_NiO_4_; and Pr(NO_3_)_3_·6H_2_O (99.9%, Sigma Aldrich) and Ni(CH_3_COO)_2_·4H_2_O (99%, Sigma Aldrich) for Pr_2_NiO_4_. Each mixture of the starting materials with 5%-excess A-site cations for perovskites and stoichiometric composition for RP phases was ball-milled in high-purity ethyl alcohol for 24 h with a zirconia jar and balls. After being dried, the slurries were calcined in air at 1100 °C for 10 h for SrTiO_3_; at 1200 °C for 10 h for Sr_2_TiO_4_ and Sr_3_Ti_2_O_7_; at 1000 °C for 8 h for Sr_2_SnO_4_; at 1050 °C for 10 h for BaCeO_3_; at 1050 °C for 12 h for LaCoO_3_ and La_2_CuO_4_; at 1100 °C for 12 h for Nd_2_NiO_4_ and Pr_2_NiO_4_; and at 1200 °C in an O_2_-flow atmosphere for 24 h for La_3_Ni_2_O_7_ for solid-state synthesis. For better sinterability and a dense microstructure, 10% Gd was doped at the Ce sites, when polycrystalline BaCeO_3_ samples were prepared. The calcined powders were ball-milled again to finally obtain fine particles. X-ray diffractometry (D/Max-2500, Rigaku) with Cu-*K*α radiation confirmed that single-phase powders were synthesized. Each of the powders was slightly pressed into disks of 10 mm diameter and isostatically pressed under 200 MPa. The pellets were sintered in air at 1400 °C for 10 h for SrTiO_3_, Sr_2_TiO_4_, and Sr_3_Ti_2_O_7_; at 1550 °C for 12 h for Sr_2_SnO_4_; at 1400 °C for 5 h for BaCeO_3_ and LaCoO_3_; at 1300 °C for 2 h for La_2_CuO_4_; at 1400 °C for 2 h for Nd_2_NiO_4_ and Pr_2_NiO_4_; and at 1350 °C for 2 h for La_3_Ni_2_O_7_ in an O_2_-flow atmosphere to obtain dense polycrystalline microstructure.

### Thin-film fabrication

Epitaxial LaNiO_3_, La_2_NiO_4_, La_2_CoO_4_, and La_4_Co_3_O_10_ thin films were fabricated by using a sol–gel process. La(NO_3_)_3_·6H_2_O (99.999%, Sigma Aldrich) and Ni(CH_3_COO)_2_·4H_2_O (99.998%, Sigma Aldrich) for LaNiO_3_ and La_2_NiO_4_, La(NO_3_)_3_·6H_2_O and Co(CH_3_COO)_2_·4H_2_O (99.999%, Alfa Aesar) for La_2_CoO_4_ and La_4_Co_3_O_10_ were used as starting materials for the preparation of precursor solutions. They were dissolved in 2-methoxyethanol under a constant stirring condition to prepare precursor solutions with 0.2 M. To induce the favorable formation of shear faults, the La-excess nonstoichiometry was controlled by adding 3 mol% more of the La nitrate for LaNiO_3_ films. For complete dissolution of the source materials, refluxing was carried out at 80 °C for 1 h. Each of the precursor solutions was deposited on (001) LaAlO_3_ and (001) SrTiO_3_ (Shinkosha, Japan) single-crystal substrates by a spin-coating method at 5000 rpm for 10 s. The wet films were dried at 150 °C for 10 min on a hot plate, and then annealed at 800 °C for 1 h in the air for LaNiO_3_ films, at 900 °C for 1 h in the air for La_2_NiO_4_ films, and at 1000 °C for 1 h in N_2_-flow atmosphere for La_2_CoO_4_ and La_4_Co_3_O_10_ films for crystallization. X-ray diffractometry (X’Pert-PRO MRD, PANalytical) and STEM observation verified the epitaxy of grown films. Commercially available (001) single-crystal substrates (MTI corporation) were purchased for structure observation in LaSrAlO_4_, LaSrGaO_4_, and NdCaAlO_4_.

### Nanocrystal synthesis

CsPbBr_3_ nanocrystals were synthesized by a microwave-assisted method^50^. A solvent mixture was prepared by using 1-octadecene (5 mL), oleylamine (0.5 mL), and oleic acid (0.5 mL). This mixture was degassed by evacuation and stirred in a round bottom flask at 170 °C on a hot plate for 30 min. After the degassing process, the mixture was transferred into a 10-mL vial and followed by adding and dispersing Cs_2_CO_3_ (0.0217 g, 99.995%, Sigma Aldrich) and PbBr_2_ (0.0738 g, 99.999%, Sigma Aldrich) with a vortex mixer. This vial was placed into a microwave oven and subsequent microwave irradiation was carried out at 700 W for 5 min. The 5-min irradiation together with 1-min intermission was further repeated eight more times, resulting in irradiation for 45 min in total. To separate residual solvents and a solid product, the mixture after the microwave irradiation was centrifuged at 1398×*g* for 5 min. The solid product was dispersed in toluene and further purified by centrifugation at 5590×*g* for 5 min so as to finally acquire a toluene solution containing homogeneously dispersed CsPbBr_3_ nanocrystals.

### STEM, EELS, and specimen preparation

Specimens for STEM observation of sintered polycrystalline samples were prepared by mechanical grinding to a thickness of 80 μm, dimpling to a thickness of <10 μm, and finally ion-beam thinning by using a precision ion polishing system (PIPS, Gatan Inc.) for electron transparency. STEM specimens for LaNiO_3_ and NdNiO_3_ thin films were prepared by lift out via ion-beam milling in a focused ion-beam system (Quanta 3D FEG and Helios G4 UX, ThermoFisher Scientific). Protective amorphous carbon and thin Pt layers were applied over the region of interest before milling. To minimize the sidewall damage and sufficiently thin the specimen for electron transparency, final milling was carried out at a voltage of ~2 kV. HAADF- and ABF-STEM images were acquired with a transmission electron microscope (Titan cubed G2 60–300, ThermoFisher Scientific) at 200 kV or 300 kV with a spherical aberration (Cs) corrector (CEOS GmbH). The optimum size of the electron probe was ~1 Å with a convergence semiangle of 19 mrad. The collection semiangles of the STEM detectors were set to 40.0–200 mrad for HAADF imaging and 9.61−53.6 (or 10.1–19.1) mrad for ABF imaging. EELS analysis was performed with a Gatan Image Filter (GIF Quantum 965, Gatan Inc.). Electron energy-loss spectra for the Ba-M_5_ (781 eV) and Ce-M_5_ (883 eV) edges were acquired for atomic-column spectrum imaging in BaCeO_3_ and with a dispersion of 0.25 eV per channel and a collection aperture of 5 mm in diameter. The O-K, Ti-L_2,3_, and Co-L_2,3_ edges were also collected in SrTiO_3_ and LaCoO_3_ samples containing shear faults to examine whether the oxidation states of Ti and Co and the orbital hybridization with oxygen vary at the fault plane.

### DFT calculations

Ab initio DFT calculations for DOS variation in LaNiO_3_ were carried out using the spin-polarized local density approximation (LDA) functional for exchange-correlation, along with the ultrasoft pseudopotentials for ionic cores, as implemented in the CASTEP code (Biovia Inc.). To consider the electron localization around Ni ions, the LDA + U method with the Hubbard *U* parameter (*U* = 4.0 eV for Ni 3*d* states) was employed. A low-spin (*t*_2*g*_^6^)(*e*_*g*_^1^) for *d*^7^ Ni^3+^ configuration was assumed for the spin-polarized calculations. The plane-wave basis set for the kinetic energy cutoff was 500 eV. Relaxation of the internal coordinates for each atom was performed using the Broyden–Fletcher–Goldfarb–Shanno (BFGS) algorithm with convergence tolerances of 0.1 eV/Å for the maximum ionic force, 5 × 10^−5^ eV/atom for the total energy, and 0.005 Å for the maximum ionic displacement.

## Supplementary information

Supplementary Information

## Data Availability

The data sets generated during and/or analyzed during the current study are available from the corresponding author on reasonable request.
