# Supplementary material for: Local-electrostatics-induced oxygen octahedral distortion in perovskite oxides and insight into the structure of Ruddlesden–Popper phases
Source: Nat Commun. 2021 Sep 20;12:5527. doi: 10.1038/s41467-021-25889-6 (PMC8452630; doi:10.1038/s41467-021-25889-6)
Supplement: Supplementary file 1 — Supplementary Information [file 41467_2021_25889_MOESM1_ESM.pdf]

Supplementary Information

Local-electrostatics-induced oxygen octahedral distortion in perovskite oxides and insight into the structure of Ruddlesden–Popper phases

Y. Hong et al.

Supplementary Table 1. Ratios of the *B*–O bond lengths in Ca-, Sr-, Ba-, and Eu-based RP oxides. Except four cases (indicated by a red-brown background), no significant elongation (>4%) is identified in each structure. The illustration below this table describes the definition of a bond-length ratio, L_z / L_x, in each oxygen octahedron. "*2.1.4.*", "*3.2.7.*", and "*4.3.10.*" represent A_2BO_4 (n = 1), $A_3B_2O_7$ (n = 2), and $A_4B_3O_{10}$ (n = 3), respectively. Note that Eu in titanates and ziconartes is divalent (Eu^{2+}) (see *Appl. Phys. Lett.* **94**, 212509 (2009) and *J. Appl. Phys.* **112**, 083719 (2012)). The asterisks (*) in $Ba_3Ti_2O_7$, $Ba_4Ti_3O_{10}$, and $Sr_3V_2O_7$ indicate that each of the bond-lengths, L_z and L_x, is the arithmetic mean value of the two distinct lengths, as the Ti (or V) off-centering is substantial (see Supplementary Fig. 22 for details). See Supplementary Figs. 7–28 for the crystal structures of the phases.

[*A* site ions: 2+]

Elongation (>4%)

Sr		L_z / L_x
$SrTiO_3$	***2.1.4.***	1.022
	3.2.7.	0.999
	4.3.10.	0.996
$SrRuO_3$	***2.1.4.***	1.077
	3.2.7.	1.053
	4.3.10.	1.023
$SrFeO_3$	***2.1.4.***	1.014
	3.2.7.	1.005
$SrMnO_3$	***2.1.4.***	1.018
	3.2.7.	0.996
$SrSnO_3$	***2.1.4.***	1.012
	3.2.7.	0.996
$SrVO_3$	***2.1.4.***	1.069
	3.2.7.	0.965*
	4.3.10.	1.011
$SrZrO_3$	***2.1.4.***	1.022
	3.2.7.	1.003
$SrCoO_3$	*2.1.4..*	0.9786

Ca		L_z / L_x
$CaTiO_3$	***2.1.4.***	1.032
	3.2.7.	1.014
	4.3.10.	1.014
$CaRuO_3$	***2.1.4.***	1.017
	3.2.7.	0.982
$CaFeO_3$	***3.2.7.***	0.999
$CaMnO_3$	***2.1.4.***	1.043
	3.2.7.	1.002
	4.3.10.	0.989
$CaSnO_3$	***3.2.7.***	0.987
$CaZrO_3$	***3.2.7.***	0.994
$CaCoO_3$	***3.2.7.***	0.993

2.1.4.: (n = 1) A_2BO_4
3.2.7.: (n = 2) $A_3B_2O_7$
4.3.10.: (n = 3) $A_4B_3O_{10}$

Ba		L_z / L_x
$BaTiO_3$	***3.2.7.***	0.981*
	4.3.10.	0.987*
$BaRuO_3$	***2.1.4.***	1.023
$BaPbO_3$	***2.1.4.***	0.995
	4.3.10.	0.995
$BaSnO_3$	***2.1.4.***	0.991
$BaZrO_3$	***2.1.4.***	1.009
	3.2.7.	0.994

Eu		L_z / L_x
$EuTiO_3$	***2.1.4***	1.038
	3.2.7	1.026
	4.3.10	1.011
$EuZrO_3$	***2.1.4***	1.021

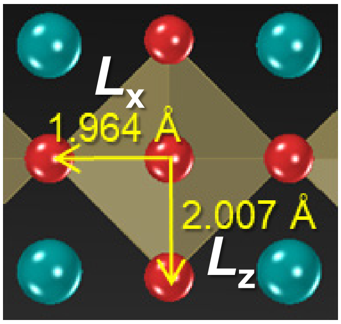

Ex) Sr_2TiO_4

L_z / L_x = 2.007 Å / 1.964 Å = 1.022

Supplementary Table 2. Ratios of the *B*–X bond lengths in K- and Cs-based RP fluorides and chlorides (X = F, Cl). no significant elongation is identified in each structure. "*2.1.4.*", "*3.2.7.*", and "*4.3.10.*" represent A_2BX_4 (n = 1), $A_3B_2X_7$ (n = 2), and $A_4B_3X_{10}$ (n = 3), respectively. See Supplementary Figs. 29–45 for the crystal structures of the phases.

[*A* site ions: 1+]

K		L_z / L_x
**$KMnF_3$**	***2.1.4.***	1.007
	3.2.7.	0.995
**$KNiF_3$**	***2.1.4.***	0.996
	3.2.7.	0.989
**$KCoF_3$**	***2.1.4.***	0.988
	3.2.7.	1.032
**$KCuF_3$**	***2.1.4.***	0.965
	3.2.7.	0.991
**$KMgF_3$**	***2.1.4.***	1.006
**$KZnF_3$**	***2.1.4.***	0.990
	3.2.7.	0.986

K		L_z / L_x
**$KMnCl_3$**	***2.1.4.***	0.975
**$KMgCl_3$**	***2.1.4.***	1.004
	3.2.7.	0.991

Cs		L_z / L_x
**$CsMnF_3$**	***2.1.4.***	0.966
**$CsCuF_3$**	***2.1.4.***	1.017
**$CsAgF_3$**	***2.1.4.***	0.965
**$CsCaF_3$**	***2.1.4.***	1.001

Cs		L_z / L_x
**$CsMnCl_3$**	***2.1.4.***	0.962
**$CsCrCl_3$**	***2.1.4.***	0.948
**$CsCdCl_3$**	***2.1.4.***	0.956
**$CsCaCl_3$**	***2.1.4.***	1.008
	3.2.7.	0.994
**$CsMgCl_3$**	***3.2.7.***	0.970

2.1.4.: (n = 1) A_2BX_4
3.2.7.: (n = 2) $A_3B_2X_7$
4.3.10.: (n = 3) $A_4B_3X_{10}$
(X = F, Cl)

Supplementary Table 3. Ratios of the B–O bond lengths in lanthanide-based RP oxides. Note that all of the bond-length ratios are substantially larger than one, directly indicating strong Jahn–Teller type elongation . "*2.1.4.*", "*3.2.7.*", and "*4.3.10.*" represent A_2BO_4 (n = 1), $A_3B_2O_7$ (n = 2), and $A_4B_3O_{10}$ (n = 3), respectively. See Supplementary Figs. 46–60 for the crystal structures of the phases.

[*A* site ions: 3+]

Elongation (>4%)

La		L_z / L_x
$LaNiO_3$	***2.1.4***	1.142
	3.2.7	1.123
	4.3.10	1.111
$LaCoO_3$	***2.1.4***	1.189
	4.3.10	1.148
$LaCuO_3$	***2.1.4***	1.292
$LaAgO_3$	***2.1.4***	1.226

Pr		L_z / L_x
$PrNiO_3$	***2.1.4***	1.141
$PrCoO_3$	***2.1.4***	1.178
$PrCuO_3$	***2.1.4***	1.270

Nd		L_z / L_x
$NdNiO_3$	***2.1.4***	1.139
	3.2.7	1.117
$NdCoO_3$	***2.1.4***	1.175
	4.3.10	1.130
$NdCuO_3$	***2.1.4***	1.276

Gd		L_z / L_x
$GdNiO_3$	***2.1.4***	1.133

Eu		L_z / L_x
$EuVO_3$	***2.1.4***	1.091
	3.2.7	1.081
$EuNiO_3$	***2.1.4***	1.131
$EuCoO_3$	***3.2.7***	1.048
$EuAlO_3$	***3.2.7***	1.082

2.1.4.: (n = 1) A_2BO_4
3.2.7.: (n = 2) $A_3B_2O_7$
4.3.10.: (n = 3) $A_4B_3O_{10}$

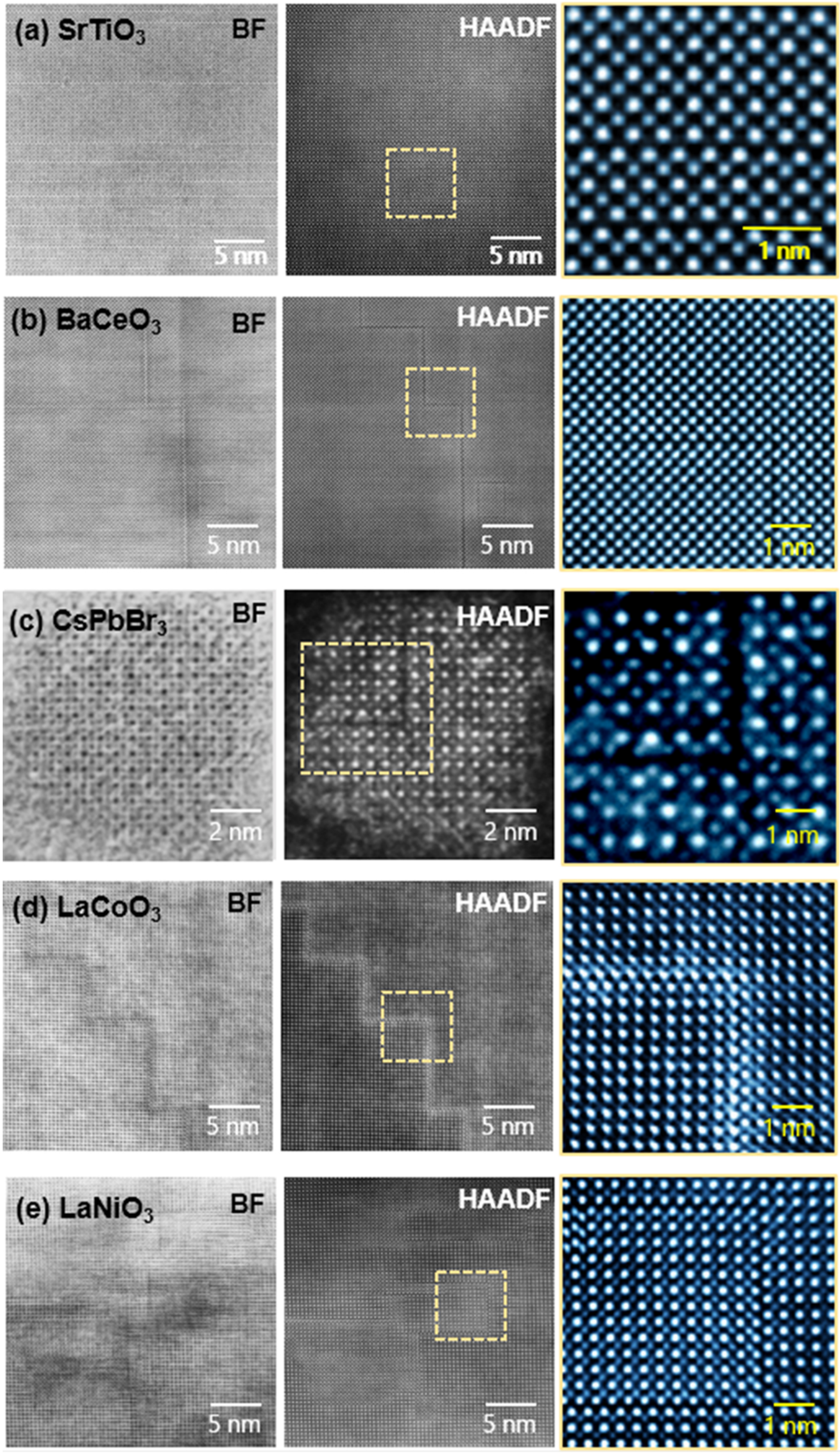

Supplementary Fig. 1. Additional STEM images showing homologous shear faults in five different perovskite materials. Pairs of low-magnification bright-field and dark-field images are shown together with enlarged images for the regions denoted by squares in (**a**) $SrTiO_3$, (**b**) $BaCeO_3$, (**c**) $CsPbBr_3$, (**d**) $LaCoO_3$, and (**e**) $LaNiO_3$.

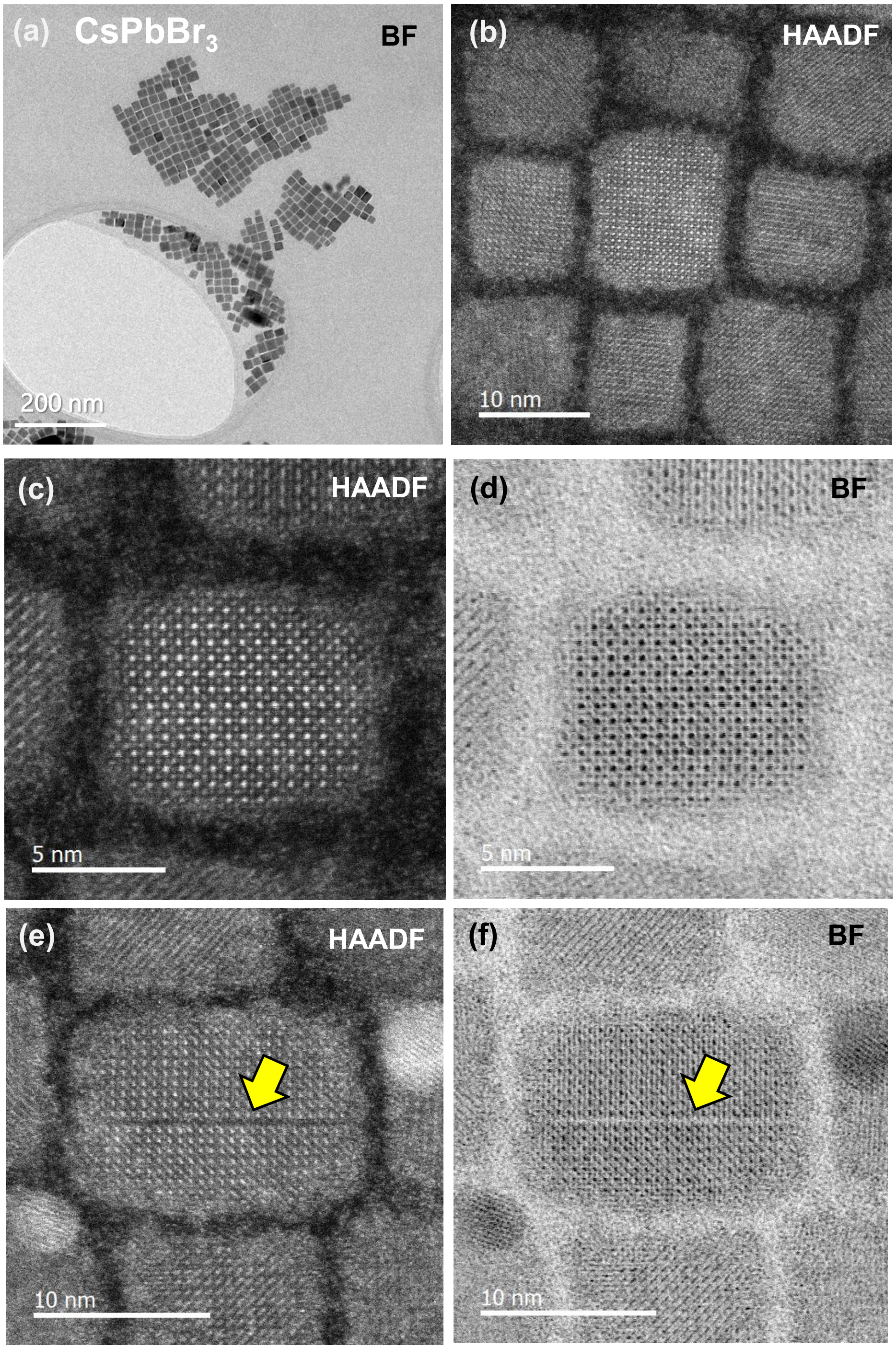

Supplementary Fig. 2 Additional STEM images showing $CsPbBr_3$ nanocrystals. (**a**,**b**) Low-magnification bright-field and HAADF images are provided to verify the successful synthesis of $CsPbBr_3$ nanocrystals by a microwave-assisted method. (**c**,**d**) A pair of atomic-column-resolved ABF and HAADF images is shown for a $CsPbBr_3$ nanocrystal without faults. (**e**,**f**) The presence of a shear fault inside a nanocrystal is indicated by a red arrow in each image.

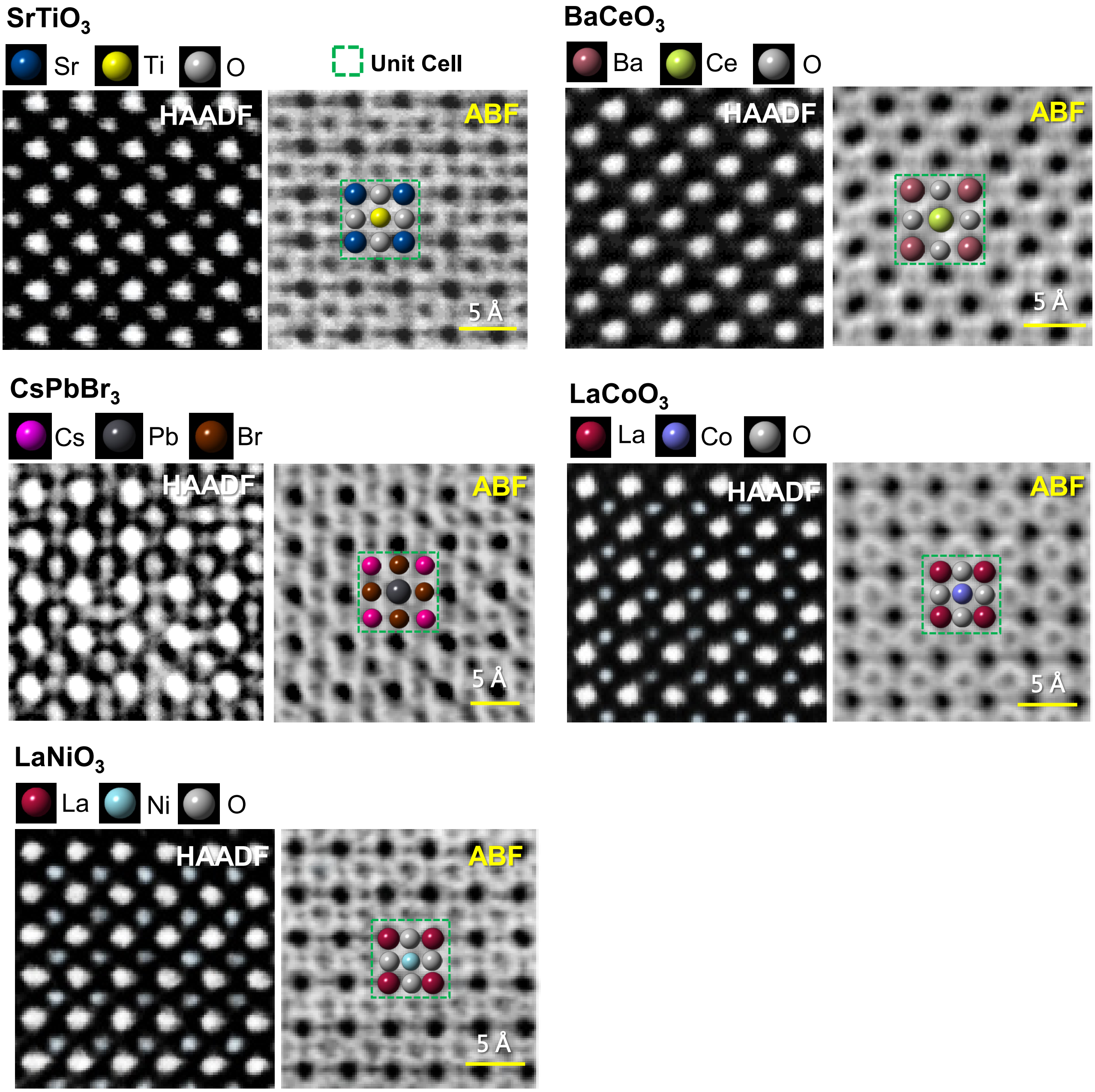

Supplementary Fig. 3 Atomic-column-resolved ABF and HAADF images of the bulk for the five perovskite phases. Note that relatively light elements, such as O and Br, are easily visualized in the ABF mode in STEM.

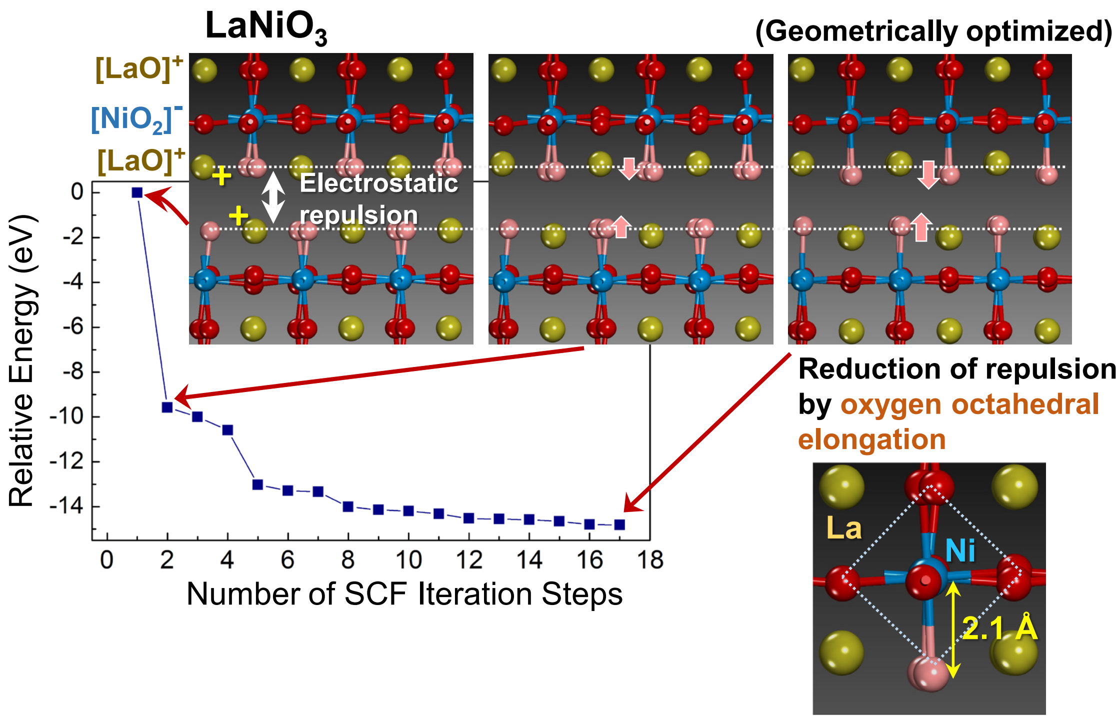

Supplementary Fig. 4 Geometry optimization of atom configuration in the fault region in $LaNiO_3$. This plot shows the relative lattice energy variation with SCF iteration steps. Lattice energy stabilization is achieved by the oxygen displacements toward the fault plane, as denoted by small arrows in the illustrations. At the same time, La^{3+} cations are also observed to displace along the *z* axis out of the fault plane in the series of supercell illustrations in order to facilitate reduction of the repulsion at the fault.

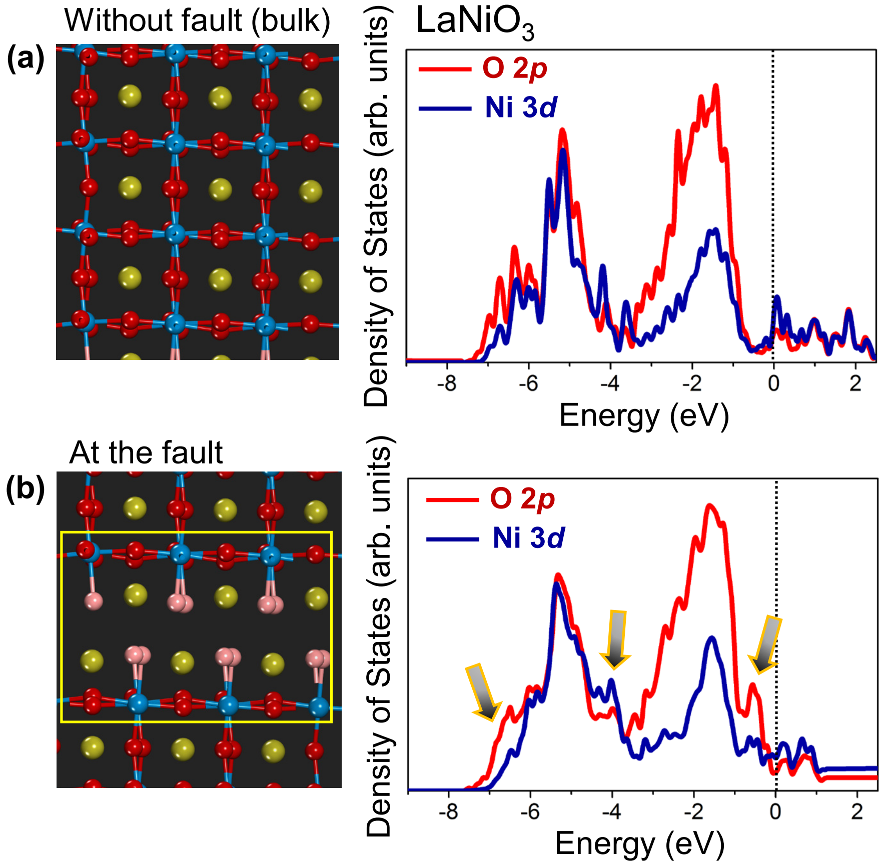

Supplementary Fig. 5 Comparison of density of states (DOS) variations with and without a fault. This set shows the DOS of (**a**) the bulk $LaNiO_3$ and (**b**) the RP fault. In addition to the metallic behavior with no bandgap, a high degree of overlap between the Ni 3*d* and O 2*p* states is noted as a common feature. However, as denoted by an arrow in the DOS plot in (b), the notable increase of O 2*p* and Ni 3*d* states near the Fermi level (E_F = 0 eV) is a major variation of the electronic structure at the fault plane where *z*-axis elongation of $[NiO_6]$ takes place. The DOS plots are reproduced from ref. 18 in the main text with permission. Copyright 2020 American Chemical Society.

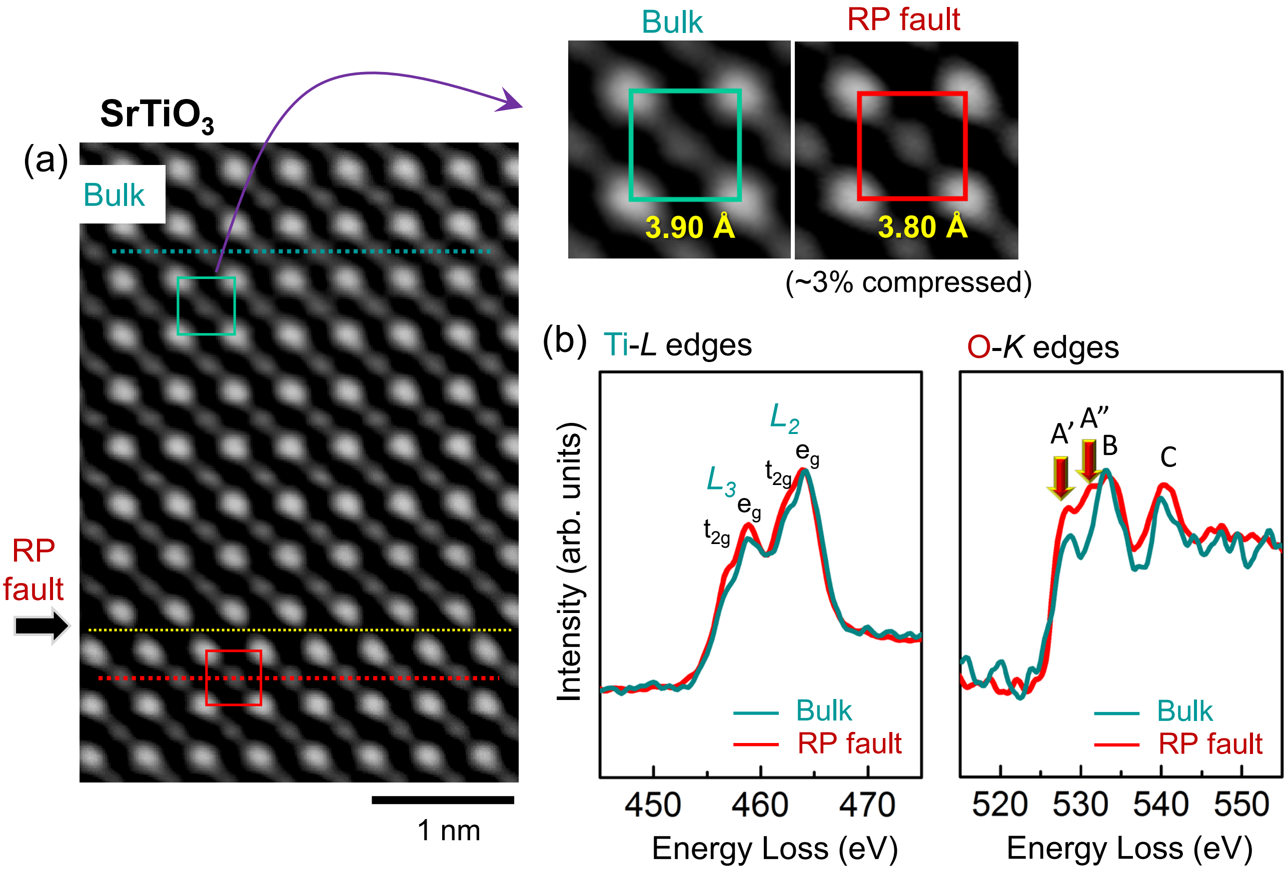

Supplementary Fig. 6 EELS analysis at the RP fault in $SrTiO_3$. (**a**) As indicated in the image, Ti-*L* and O-*K* edges were acquired from the bulk (cyan broken line) and the RP fault (red broken line) in $SrTiO_3$. Two magnified images are provided to show that the lattice parameter of a unit cell at the fault plane is ~3% smaller than that of a unit cell in the bulk. Consequently, each unit cell at the fault plane is in a compressed state. (**b**) The major peaks in the O *K*-edges represent the hybridizations of O 2*p* with Ti 3*d* t_{2g} (A'), Ti 3*d* e_g (A''), Sr 4*d* (B), and Ti 4*s*/4*p* (C) orbitals in $SrTiO_3$. No noticeable peak shift is observed in the Ti-$L_{2,3}$ edges, showing the invariant oxidation states of Ti^{4+} at the fault plane. Red arrows in the plot of O-*K* edges indicate the intensity increment of peaks A' and A'' at the fault (red curve). The reduction of lattice parameters results in a relative decrease of the Ti–O bond length. Therefore, on the contrary to the results for $LaCoO_3$, the higher intensities of peaks A' and A'' for the hybridized O 2*p* bands with Ti 3*d* bands are reasonably understood on the basis of the slightly compressed lattice at the fault.

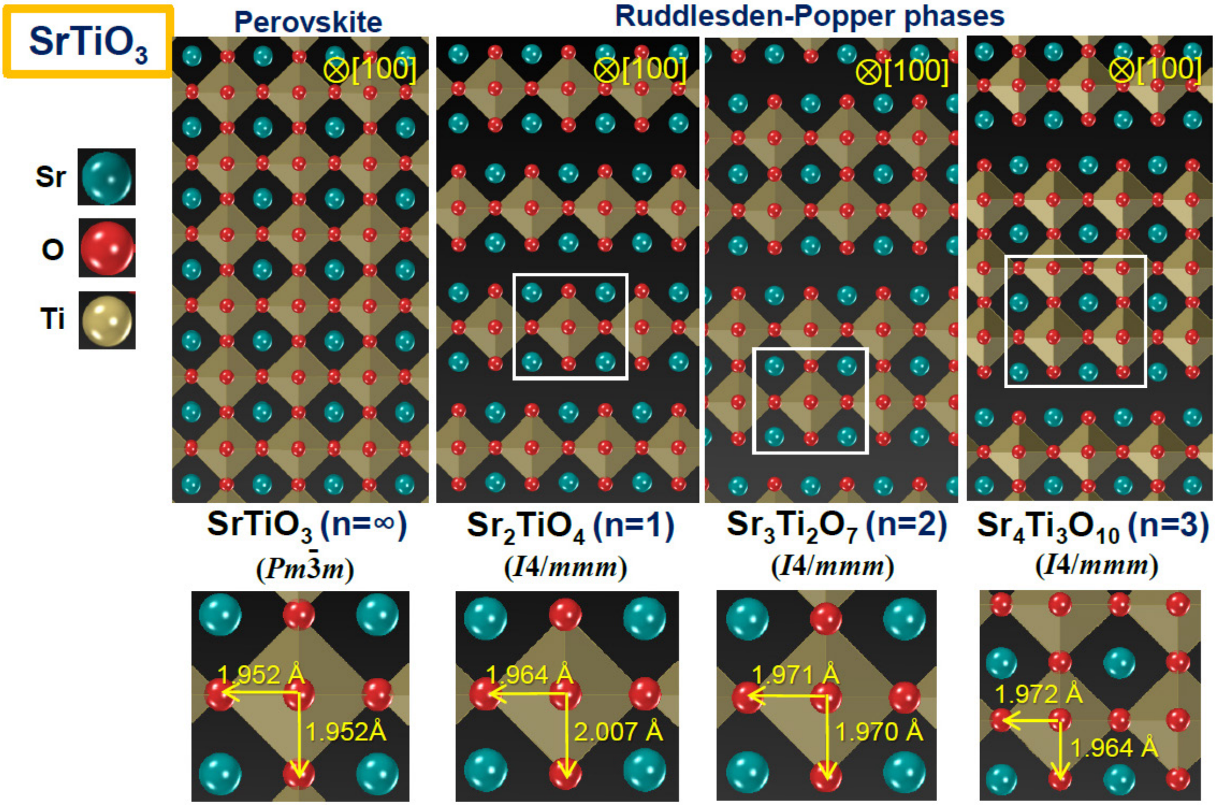

Supplementary Fig. 7 Crystal structures of Sr–Ti RP oxides along with the perovskite counterpart. No substantial *z*-axis elongation of oxygen octahedra in each phase is observed.

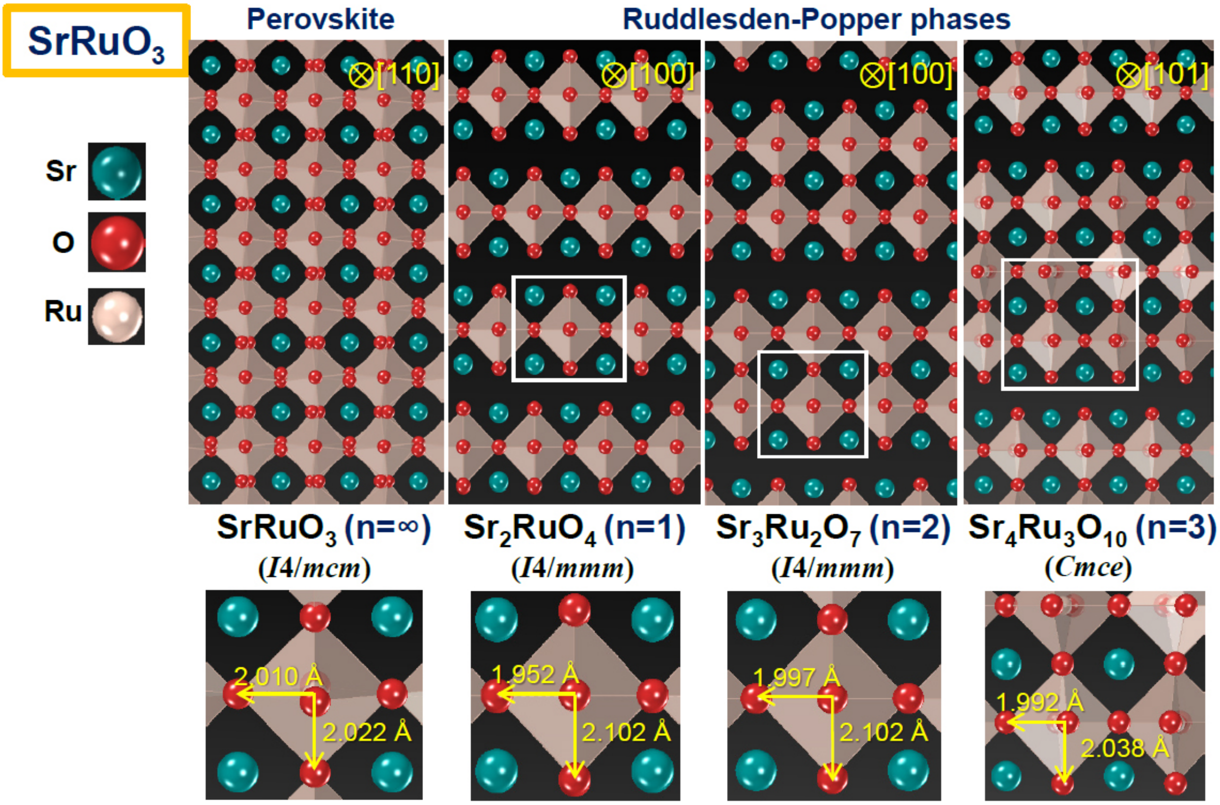

Supplementary Fig. 8 Crystal structures of Sr–Ru RP oxides along with the perovskite counterpart. Approximately 5–8% *z*-axis elongation of oxygen octahedra in Sr_2RuO_4 and $Sr_3Ru_2O_7$ is identified as exceptions.

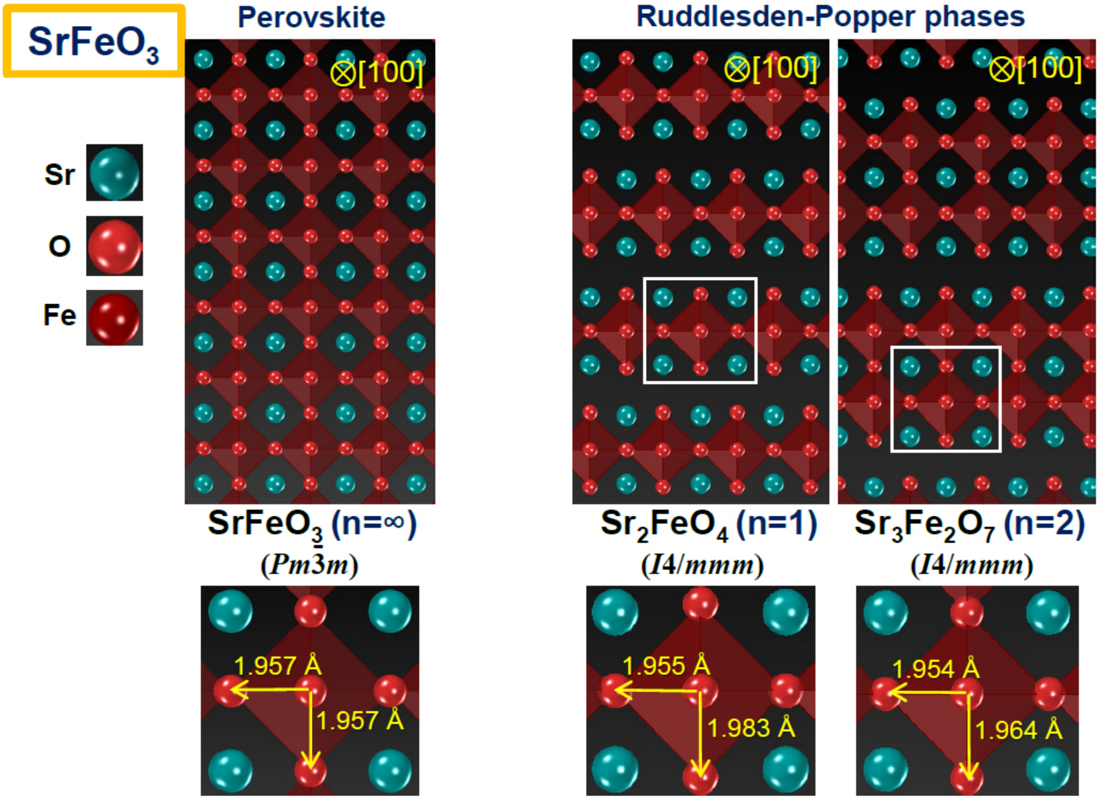

Supplementary Fig. 9 Crystal structures of Sr–Fe RP oxides along with the perovskite counterpart. No substantial *z*-axis elongation of oxygen octahedra in each phase is observed.

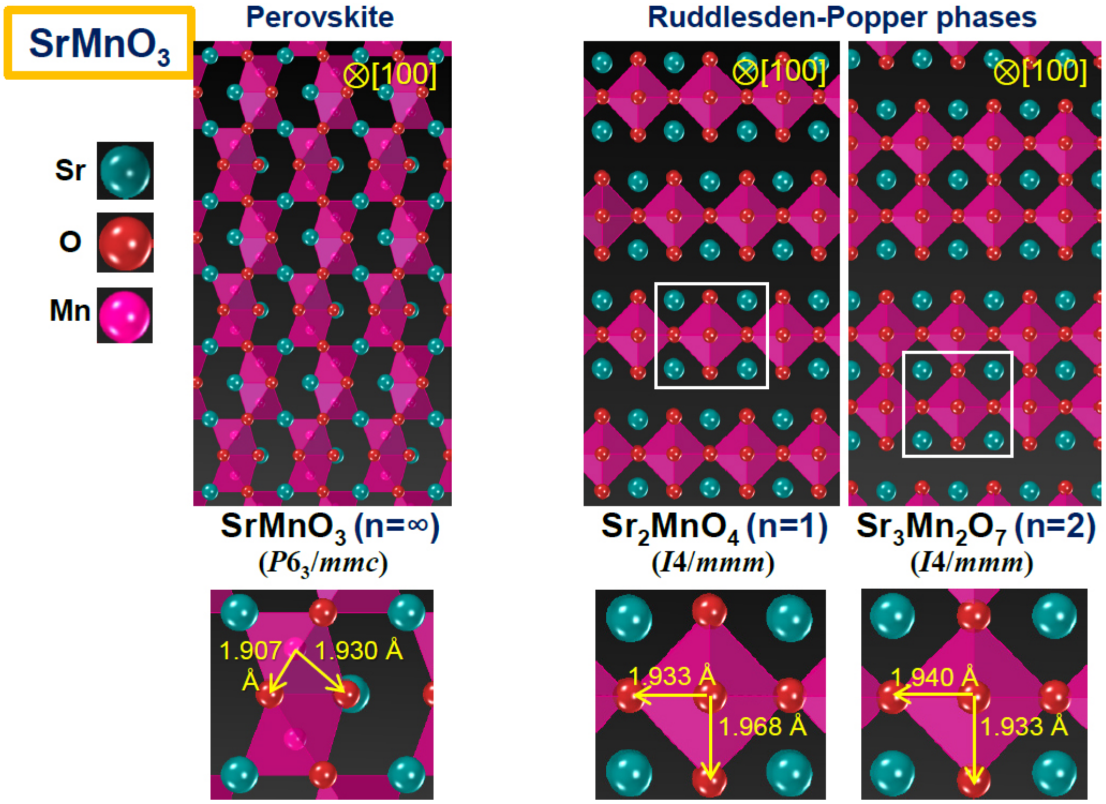

Supplementary Fig. 10 Crystal structures of Sr–Mn RP oxides along with the perovskite counterpart. No substantial *z*-axis elongation of oxygen octahedra in each phase is observed.

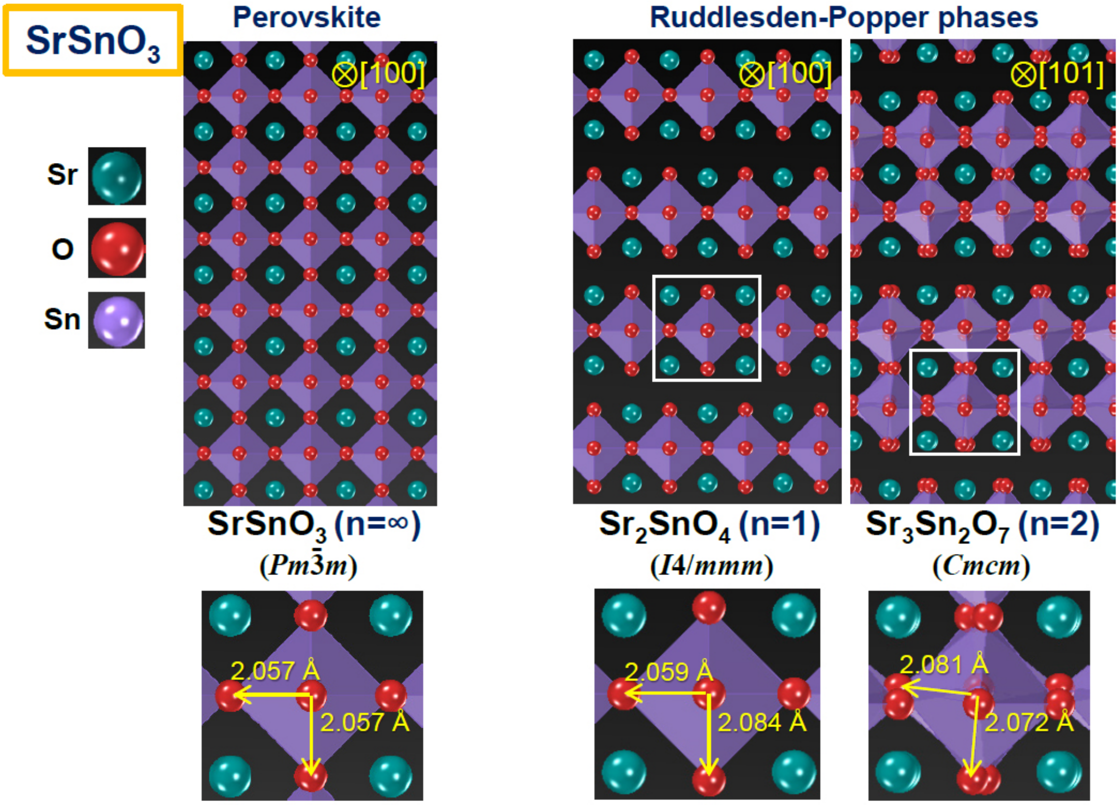

Supplementary Fig. 11 Crystal structures of Sr–Sn RP oxides along with the perovskite counterpart. No substantial *z*-axis elongation of oxygen octahedra in each phase is observed.

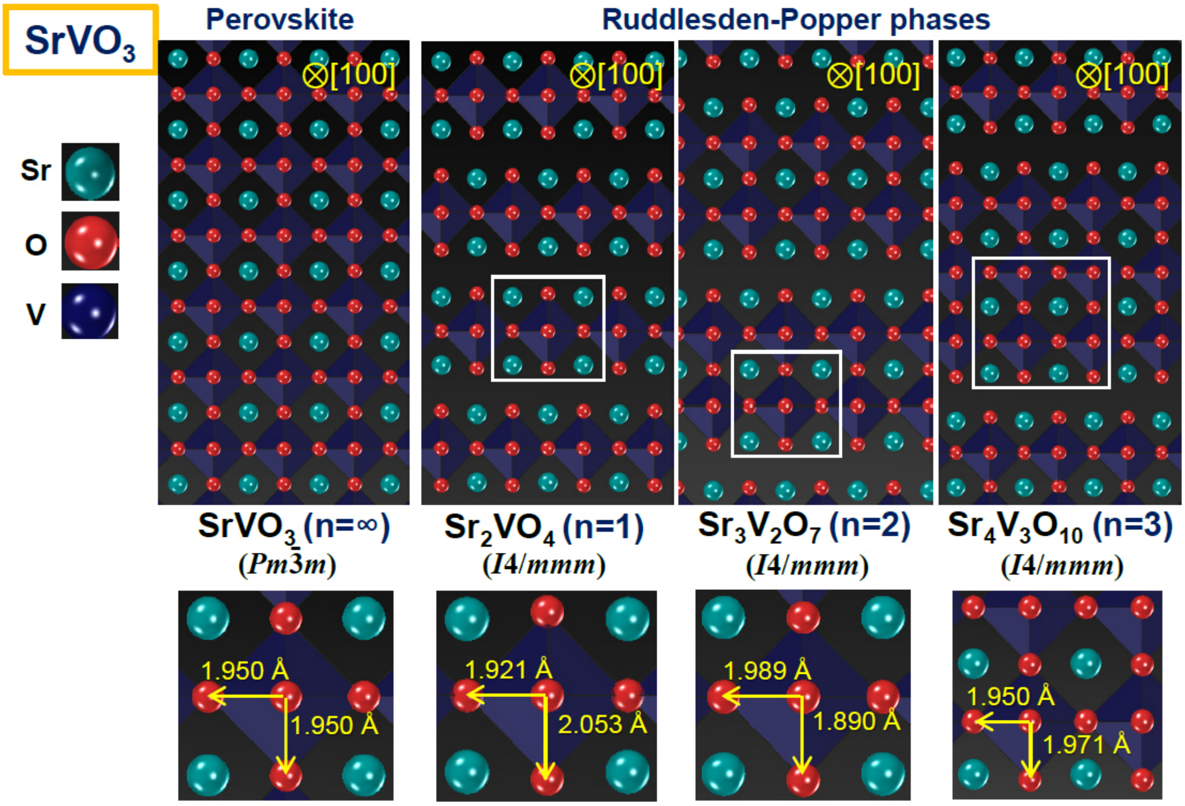

Supplementary Fig. 12 Crystal structures of Sr–V RP oxides along with the perovskite counterpart.

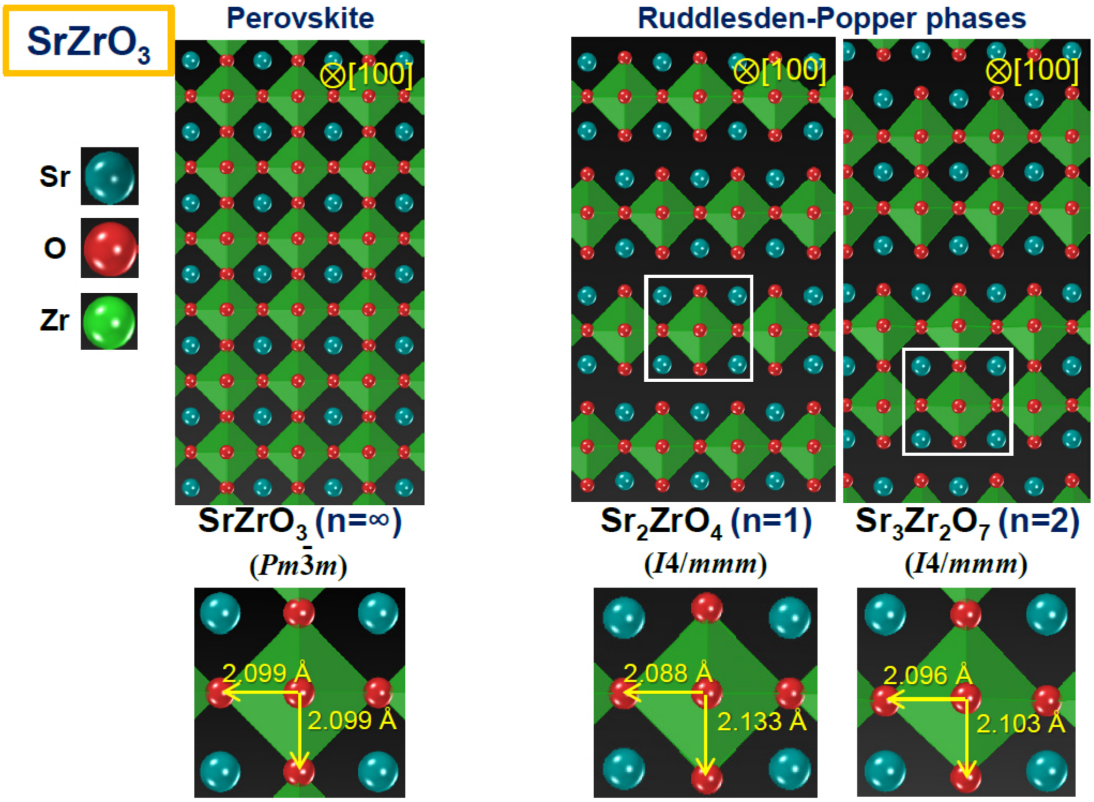

Supplementary Fig. 13 Crystal structures of Sr–Zr RP oxides along with the perovskite counterpart. No substantial *z*-axis elongation of oxygen octahedra in each phase is observed.

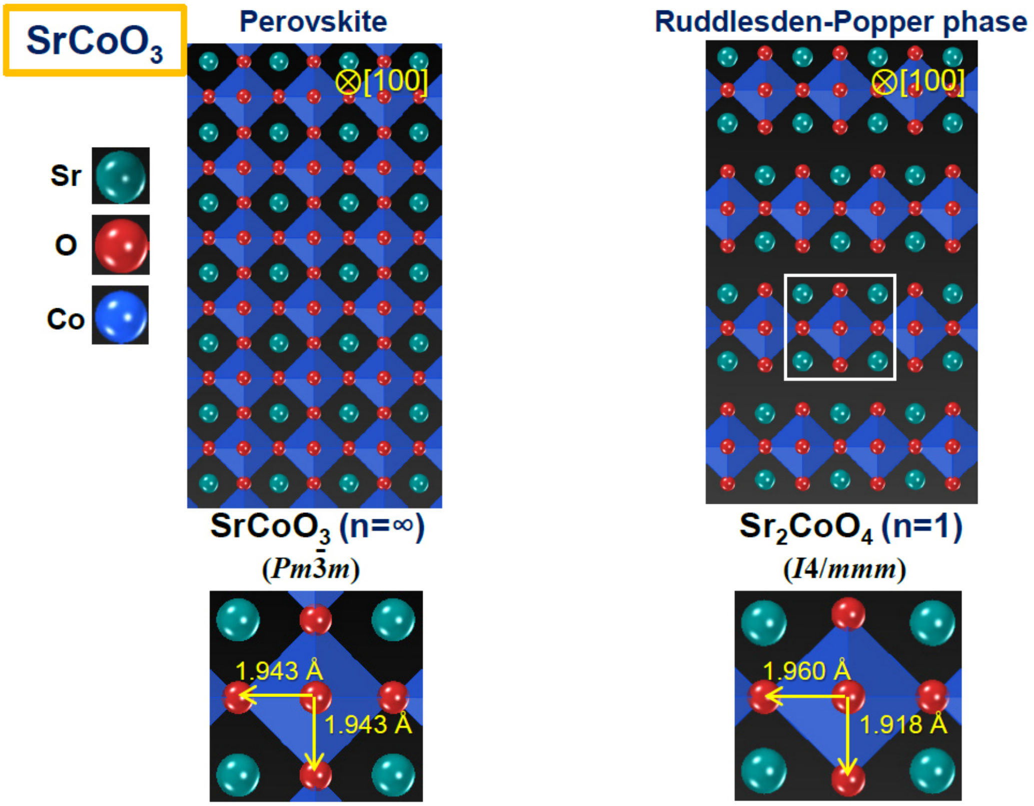

Supplementary Fig. 14 Crystal structures of Sr_2CoO_4 and its perovskite counterpart. No substantial *z*-axis elongation of oxygen octahedra is observed.

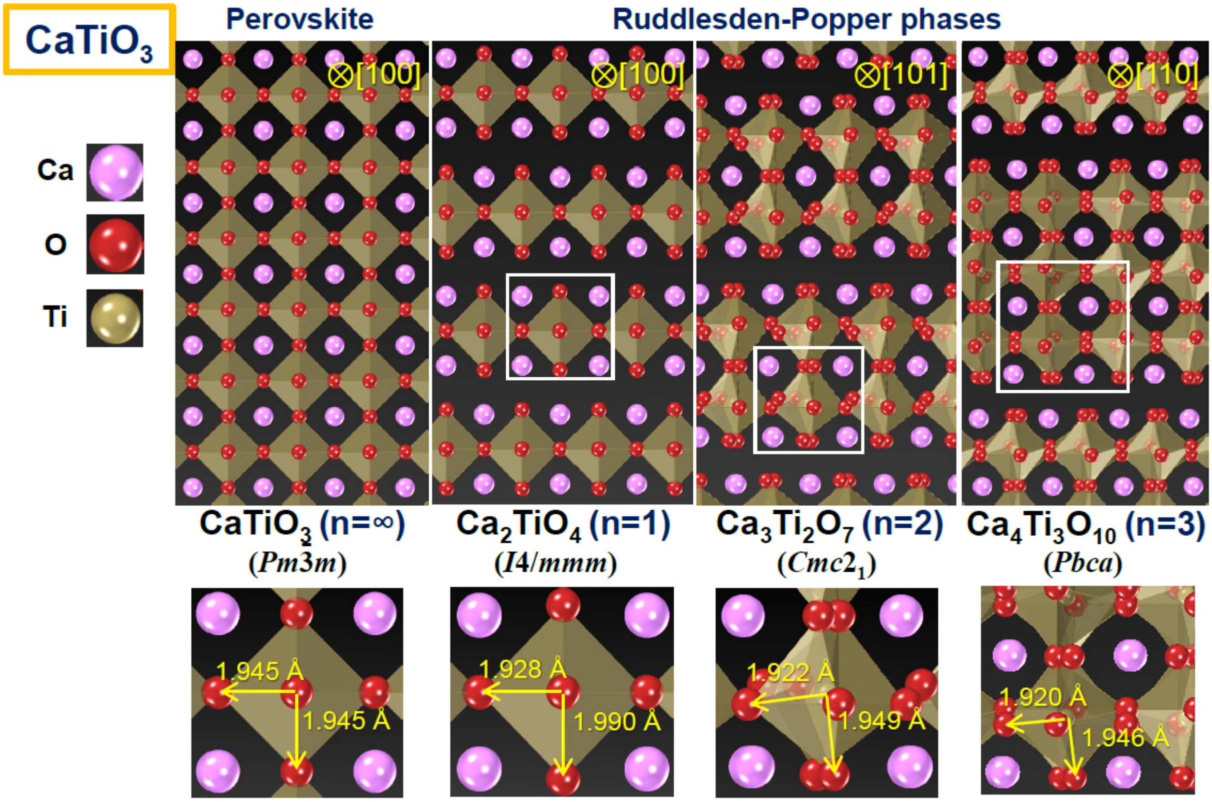

Supplementary Fig. 15 Crystal structures of Ca–Ti RP oxides along with the perovskite counterpart. No substantial *z*-axis elongation of oxygen octahedra in each phase is observed.

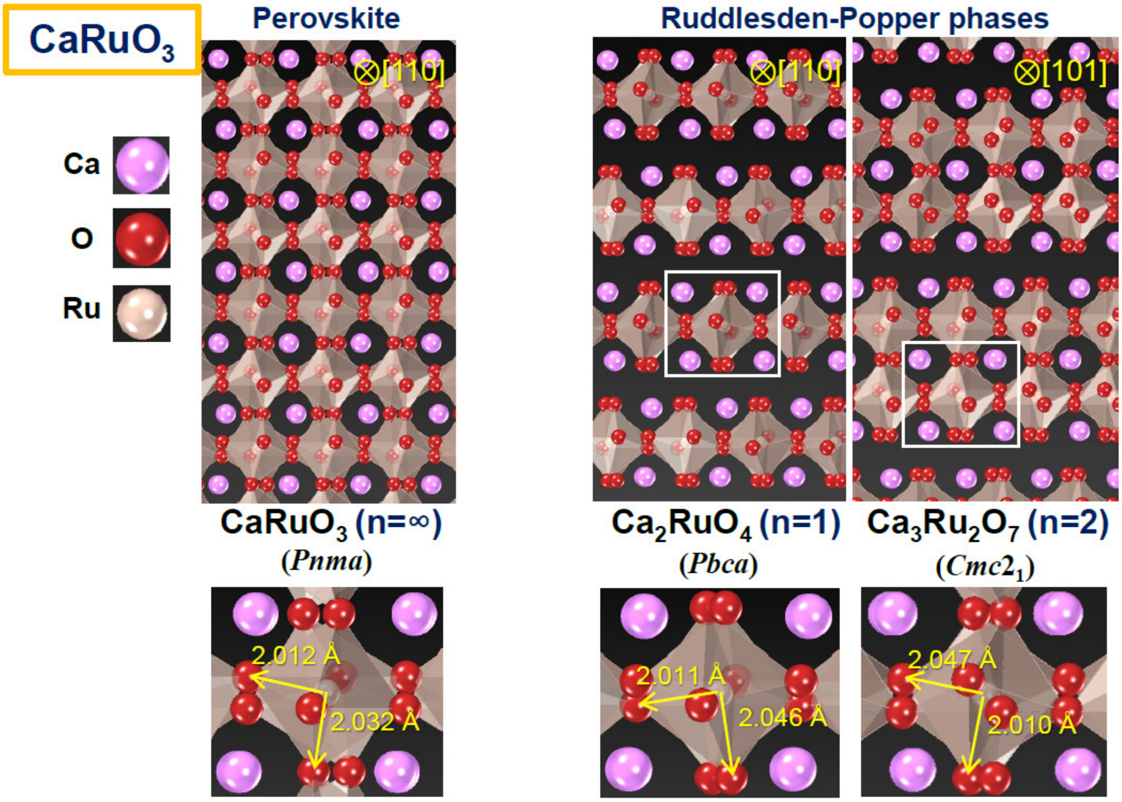

Supplementary Fig. 16 Crystal structures of Ca–Ru RP oxides along with the perovskite counterpart. No substantial *z*-axis elongation of oxygen octahedra in each phase is observed.

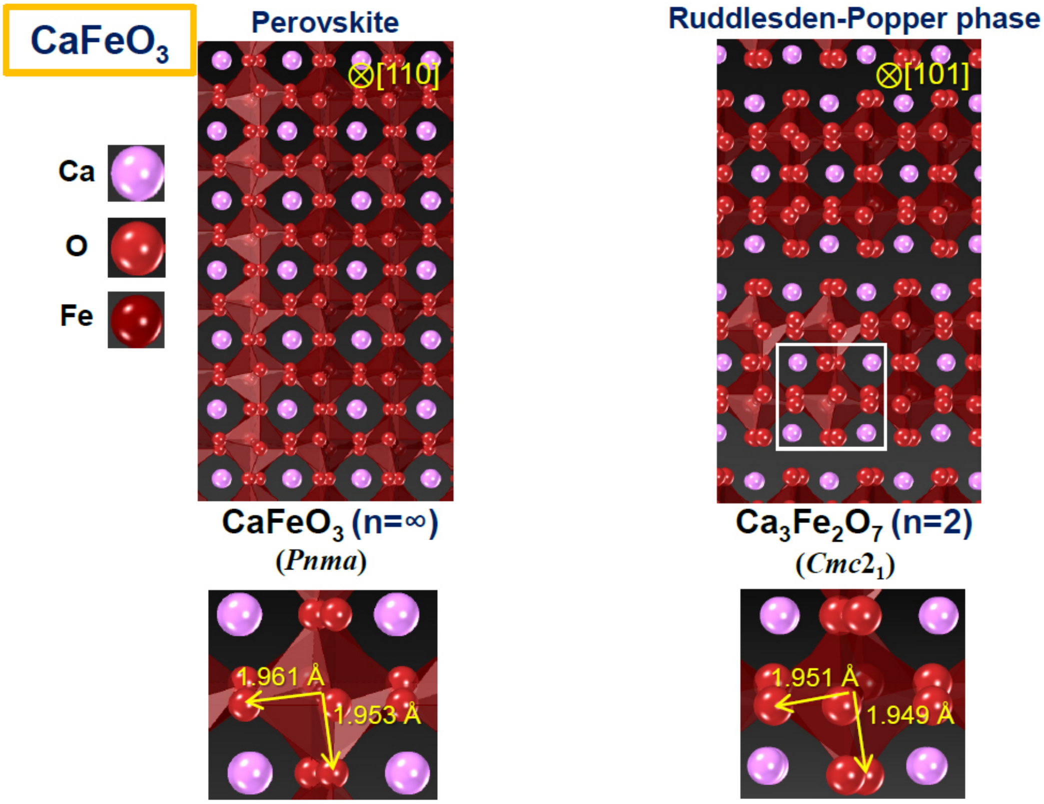

Supplementary Fig. 17 Crystal structures of $Ca_3Fe_2O_7$ and its perovskite counterpart. No substantial *z*-axis elongation of oxygen octahedra is observed.

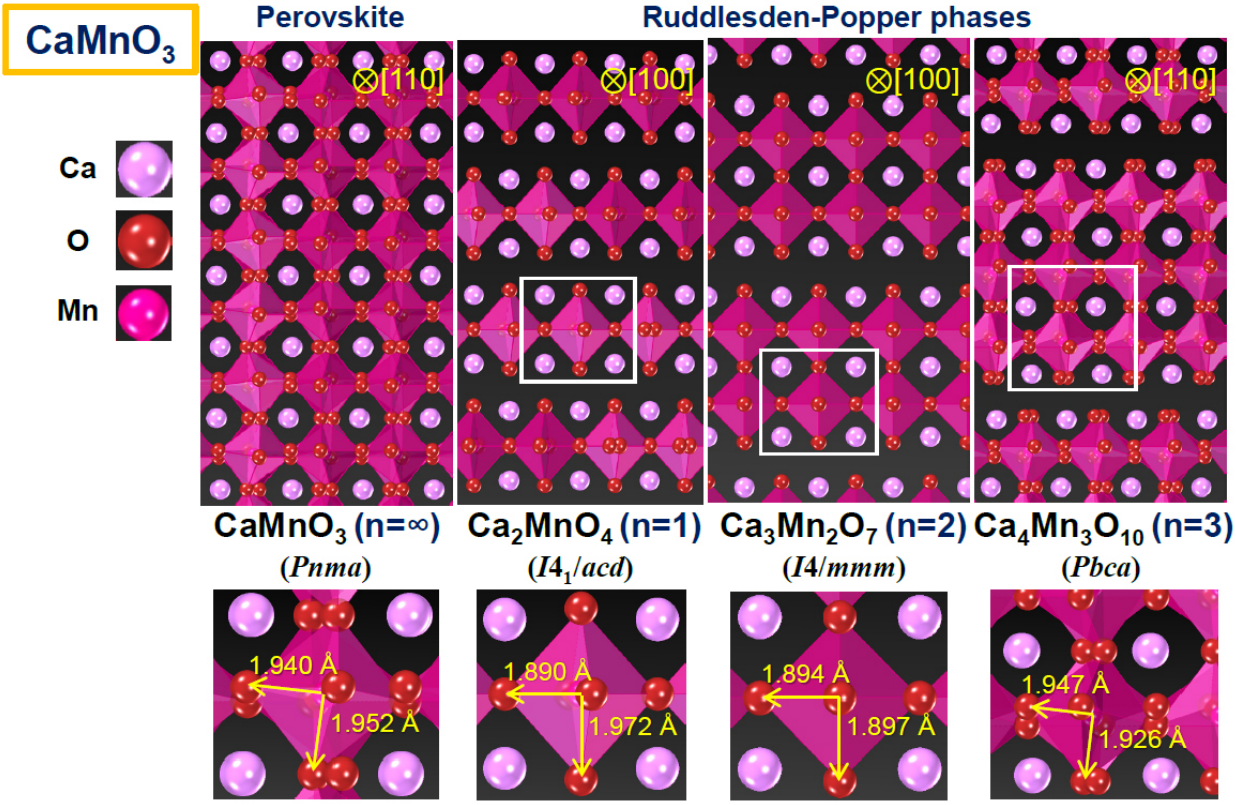

Supplementary Fig. 18 Crystal structures of Ca–Mn RP oxides along with the perovskite counterpart. No significant *z*-axis elongation of oxygen octahedra in each phase is observed.

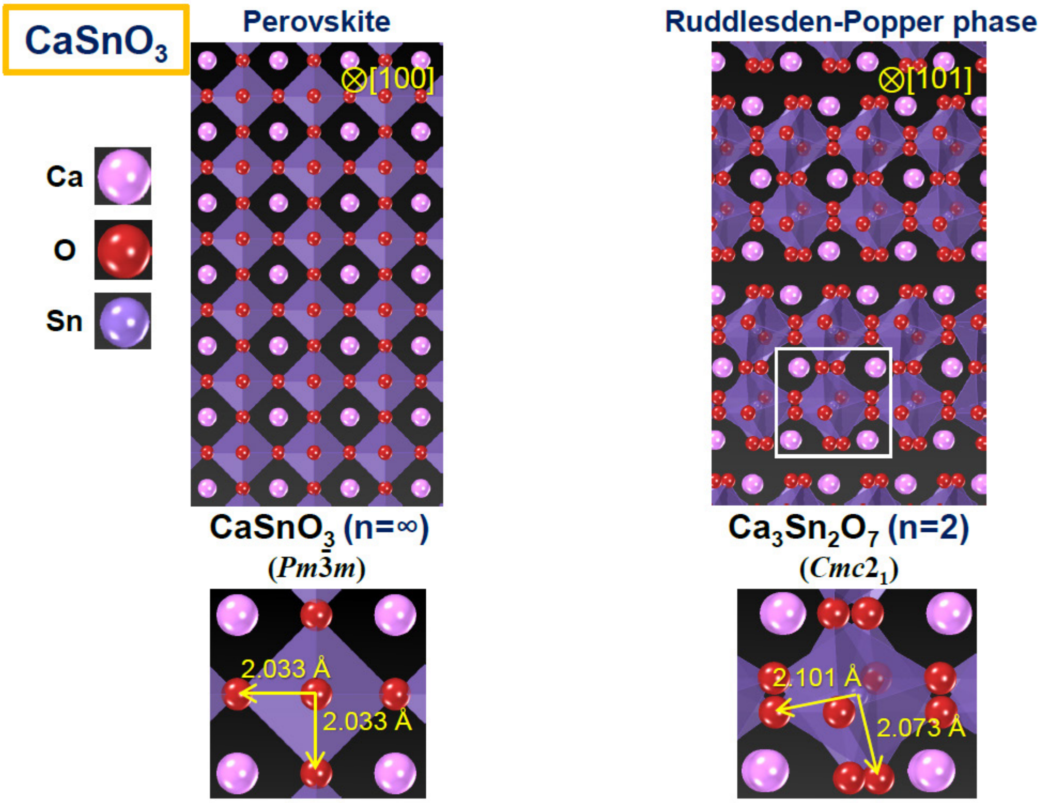

Supplementary Fig. 19 Crystal structures of $Ca_3Sn_2O_7$ and its perovskite counterpart. No substantial *z*-axis elongation of oxygen octahedra is observed.

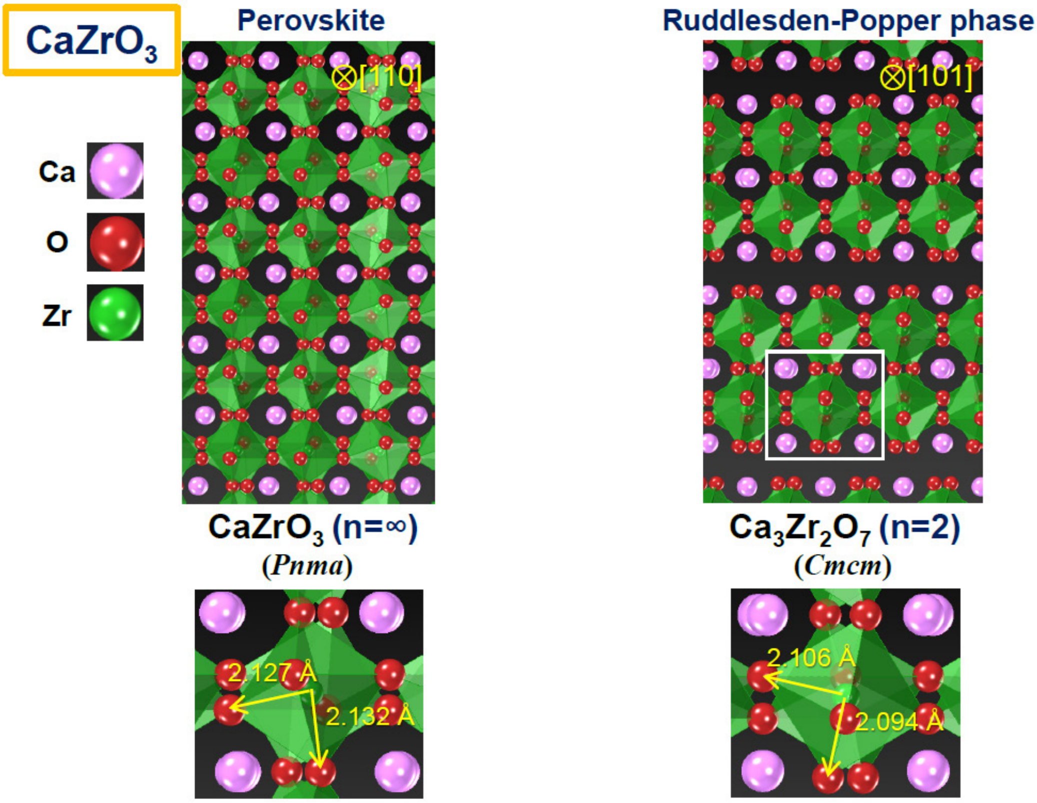

Supplementary Fig. 20 Crystal structures of $Ca_3Zr_2O_7$ and its perovskite counterpart. No significant *z*-axis elongation of oxygen octahedra is observed.

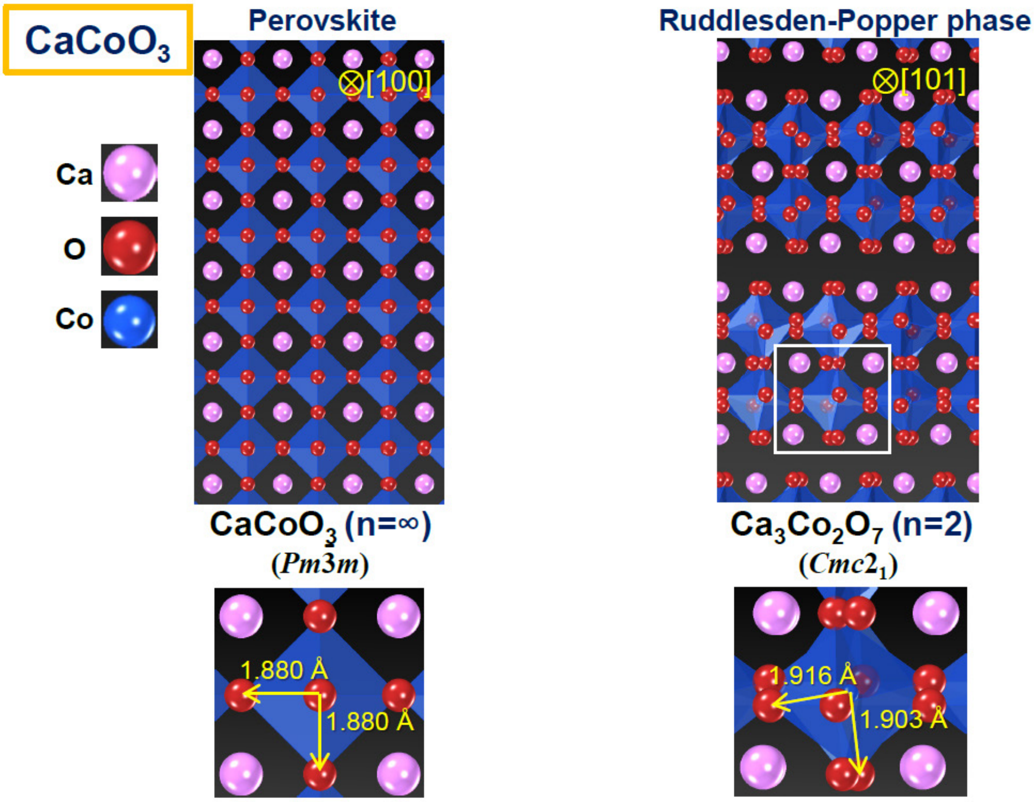

Supplementary Fig. 21 Crystal structures of $Ca_3Co_2O_7$ and its perovskite counterpart. No substantial *z*-axis elongation of oxygen octahedra is observed.

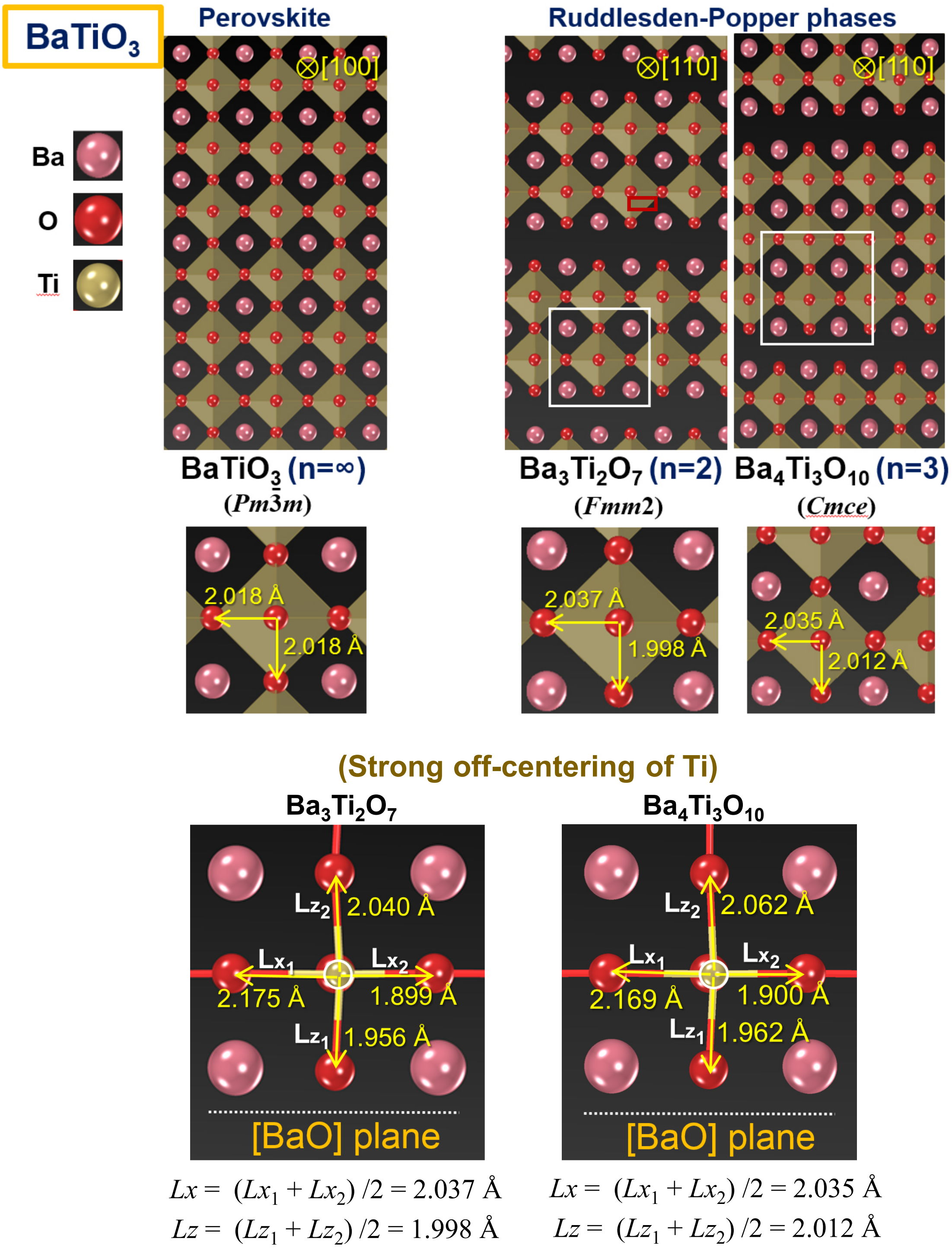

Supplementary Fig. 22 Crystal structures of Ba–Ti RP oxides along with the perovskite counterpart. As shown in the lower panel, Ti off-centering at each oxygen octahedron is significant in $Ba_3Ti_2O_7$ and $Ba_4T_3O_{10}$. Therefore, we take the arithmetic mean values for *Lx* and *Lz* in each crystal system to consider this off-centering geometry.

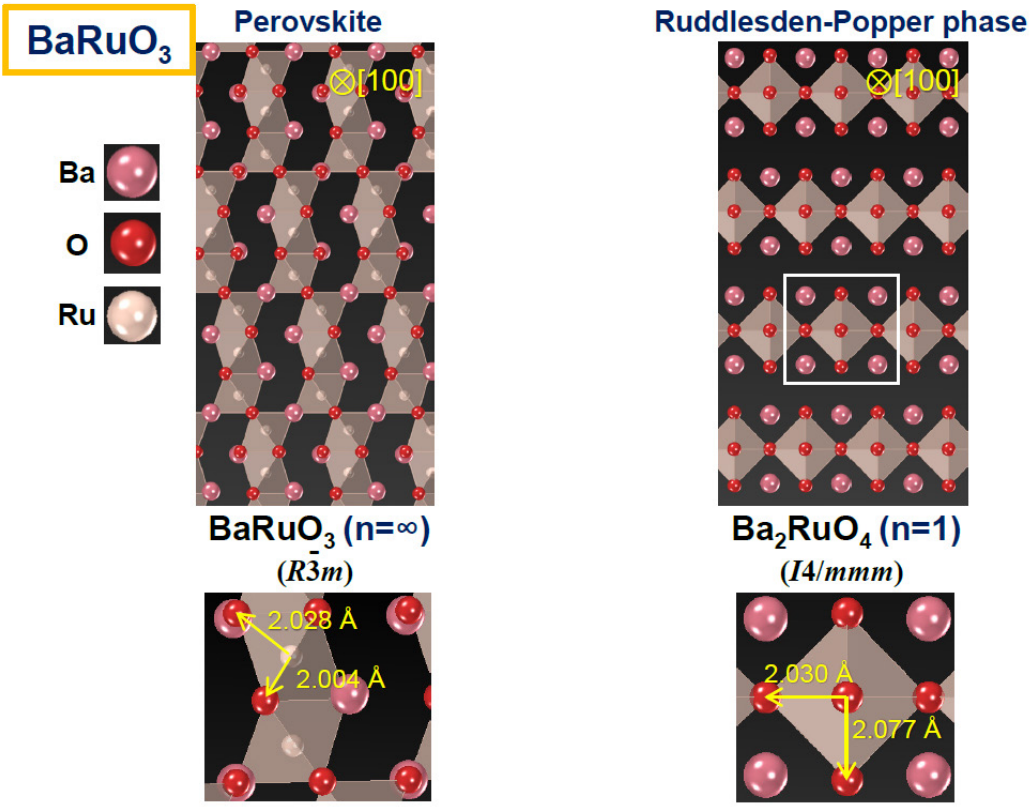

Supplementary Fig. 23 Crystal structures of Ba_2RuO_4 and its perovskite counterpart. No substantial *z*-axis elongation of oxygen octahedra is observed.

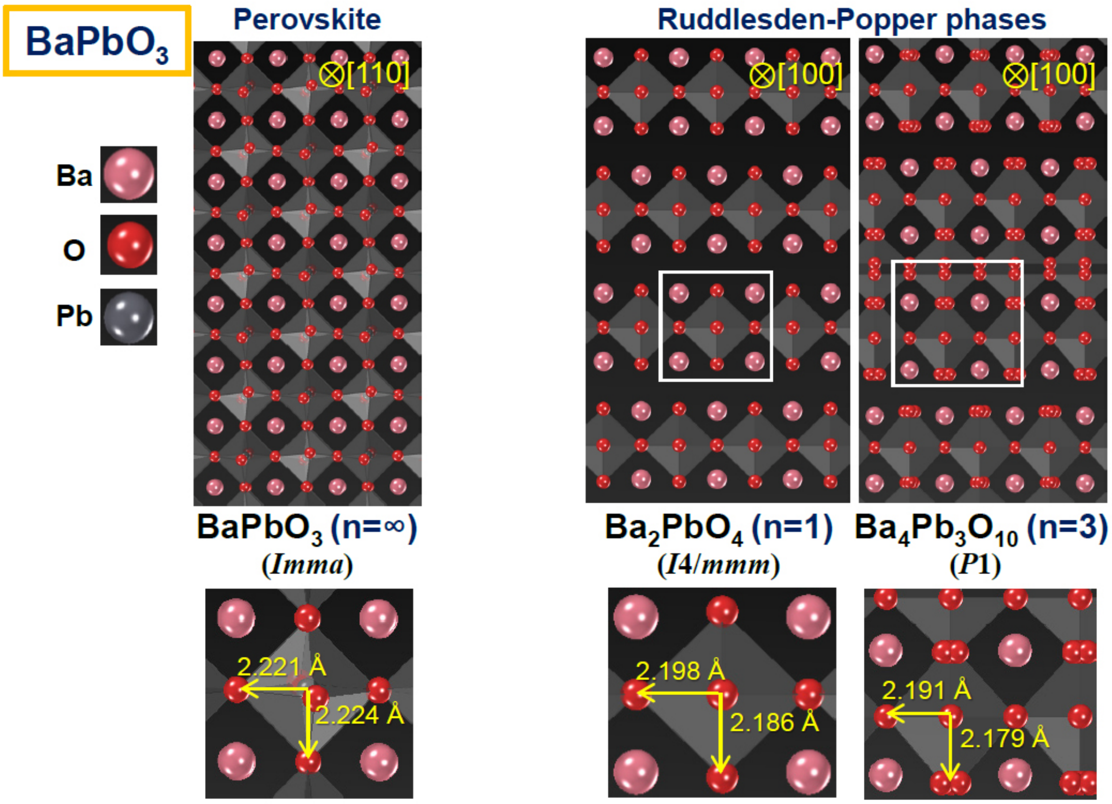

Supplementary Fig. 24 Crystal structures of Ba–Pb RP oxides along with the perovskite counterpart. No significant *z*-axis elongation of oxygen octahedra in each phase is observed.

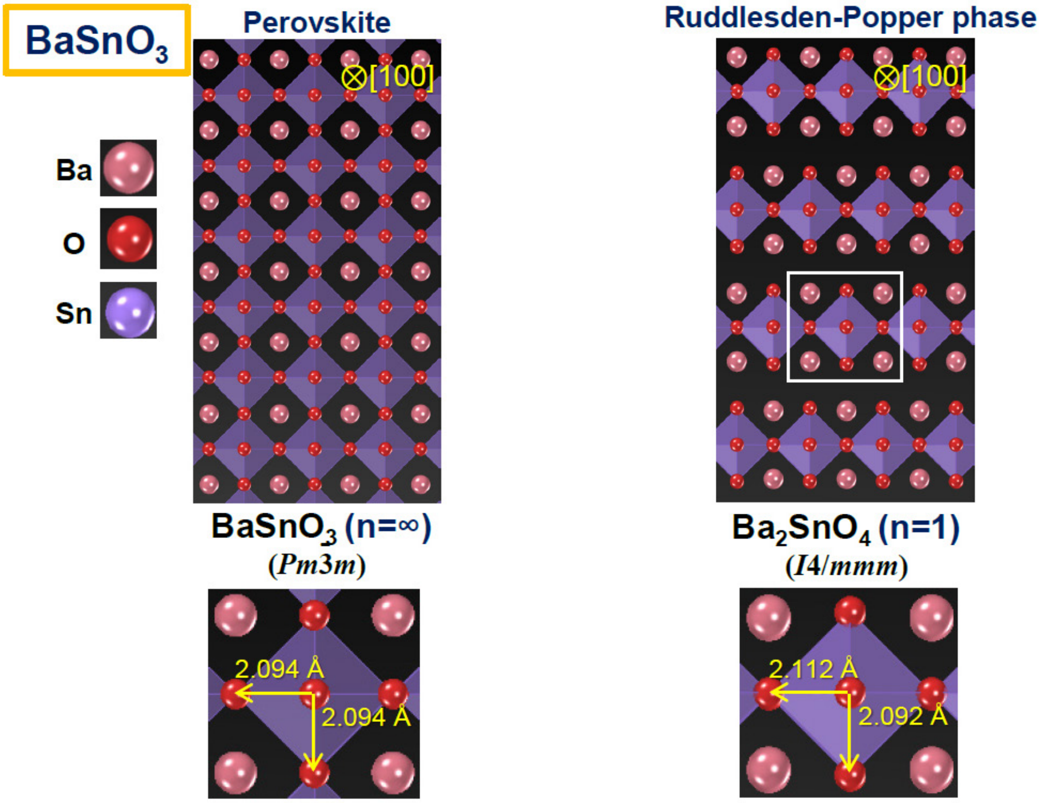

Supplementary Fig. 25 Crystal structures of Ba_2SnO_4 and its perovskite counterpart. No substantial *z*-axis elongation of oxygen octahedra is observed.

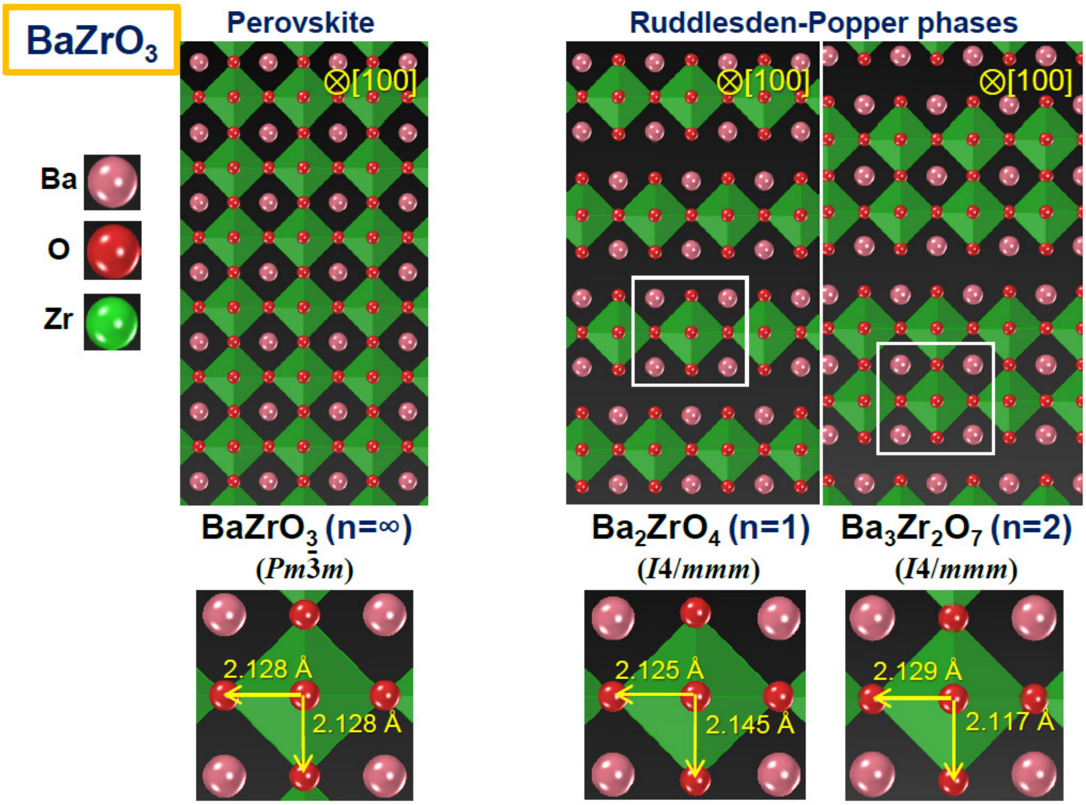

Supplementary Fig. 26 Crystal structures of Ba–Zr RP oxides along with the perovskite counterpart. No significant *z*-axis elongation of oxygen octahedra in each phase is observed.

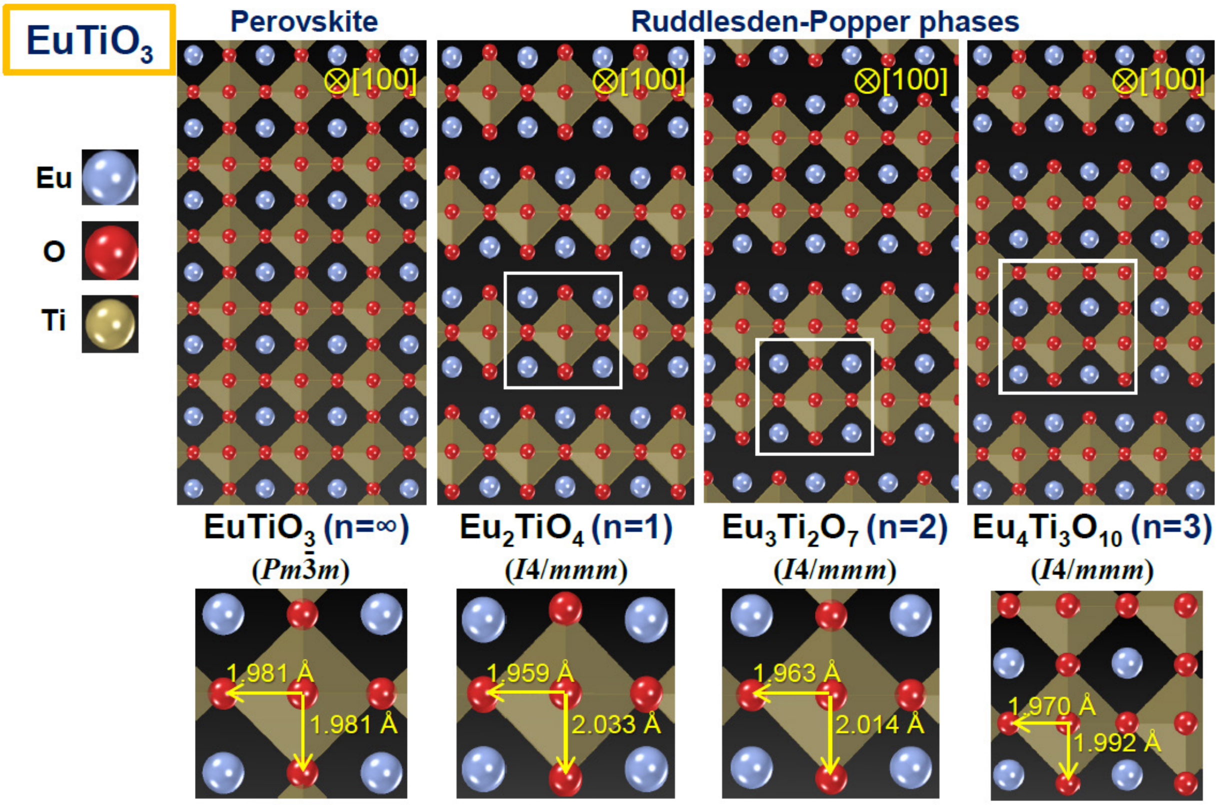

Supplementary Fig. 27 Crystal structures of Eu–Ti RP oxides along with the perovskite counterpart. No significant z-axis elongation of oxygen octahedra in each phase is observed. Note that Eu is divalent (Eu^{2+}).

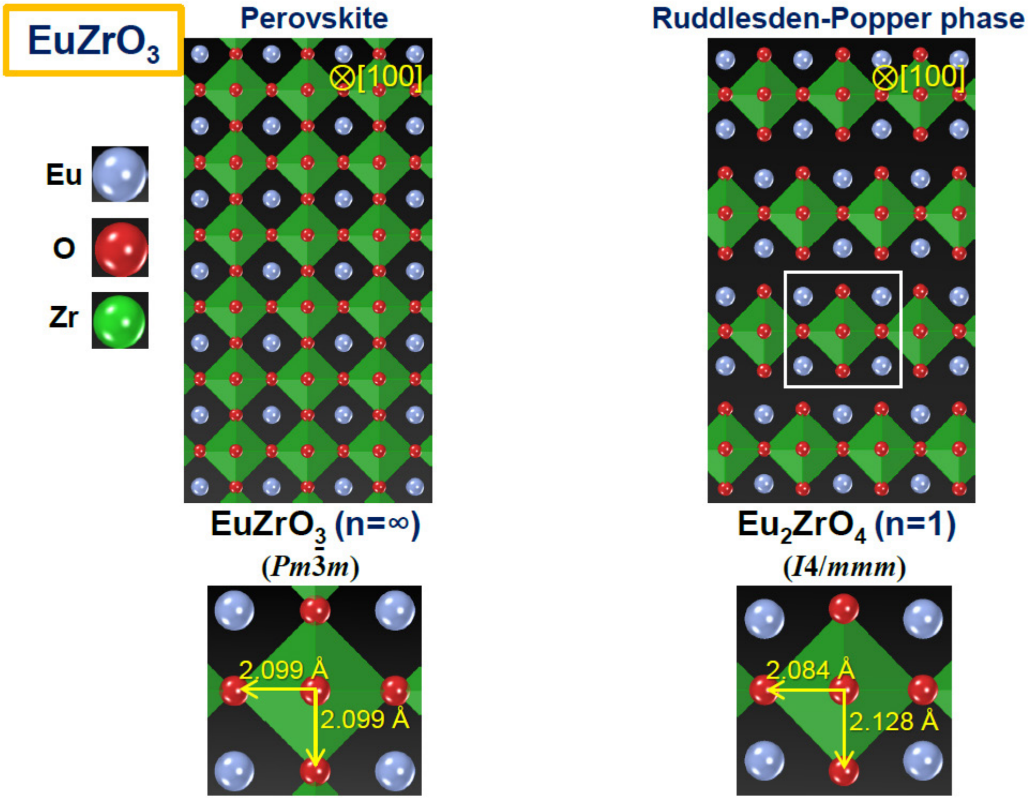

Supplementary Fig. 28 Crystal structures of Eu_2ZrO_4 and its perovskite counterpart. No significant z-axis elongation of oxygen octahedra in each phase is observed. Note that Eu is divalent (Eu^{2+}).

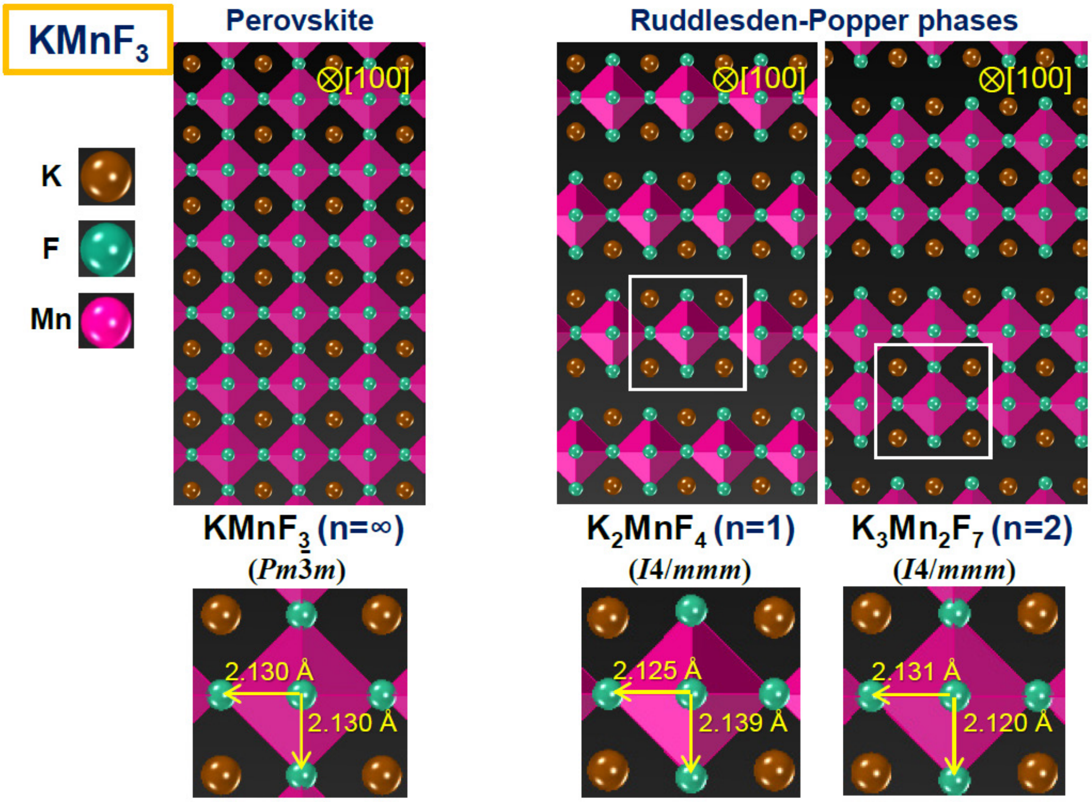

Supplementary Fig. 29 Crystal structures of K–Mn RP fluorides along with the perovskite counterpart. No substantial *z*-axis elongation of F-anion octahedra is observed.

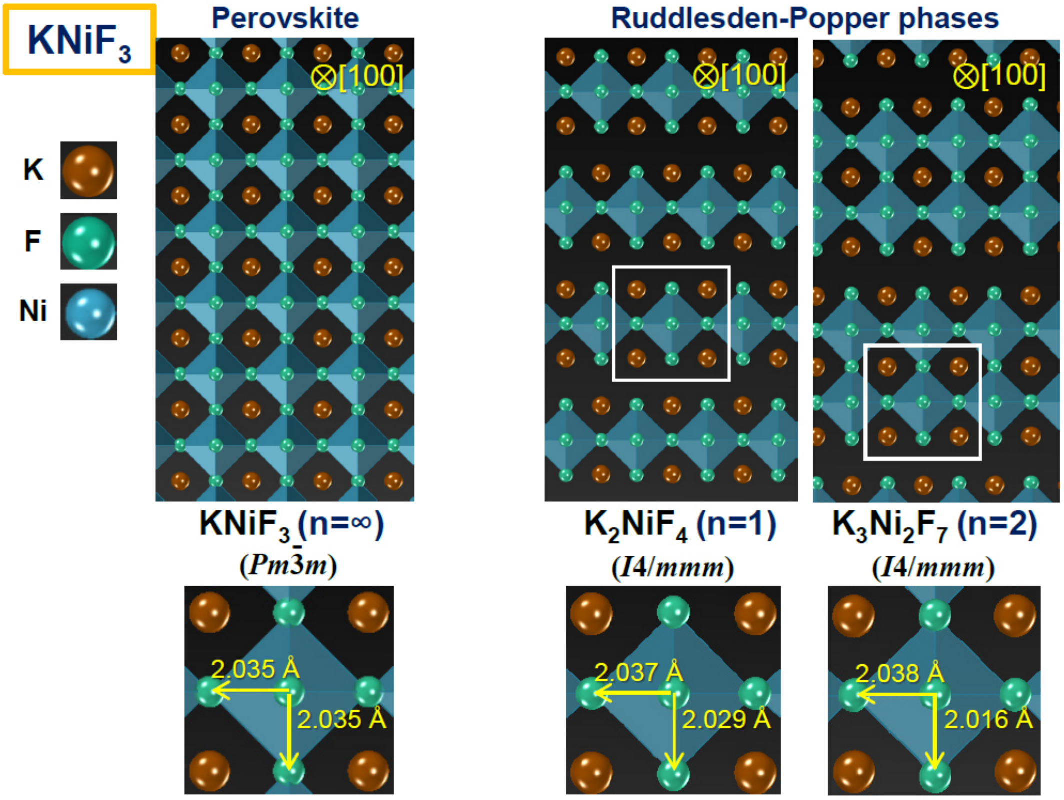

Supplementary Fig. 30 Crystal structures of K–Ni RP fluorides along with the perovskite counterpart. No significant *z*-axis elongation of F-anion octahedra in each phase is observed.

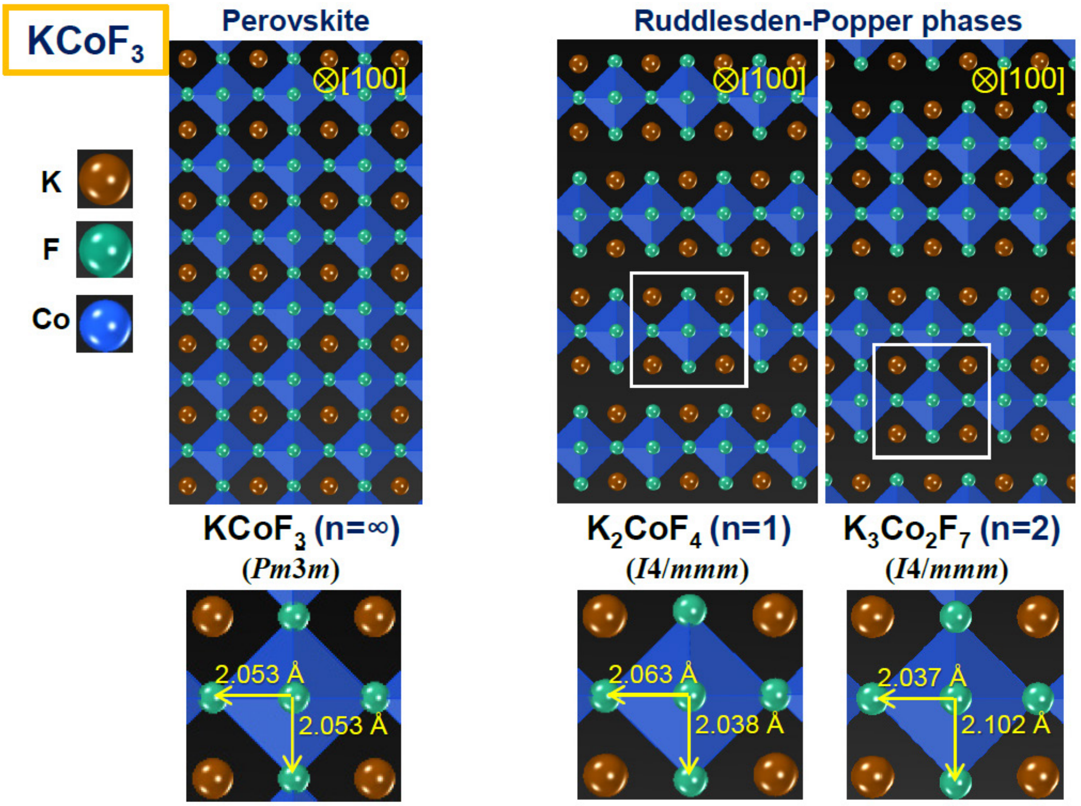

Supplementary Fig. 31 Crystal structures of K–Co RP fluorides along with the perovskite counterpart. No substantial *z*-axis elongation of F-anion octahedra is observed.

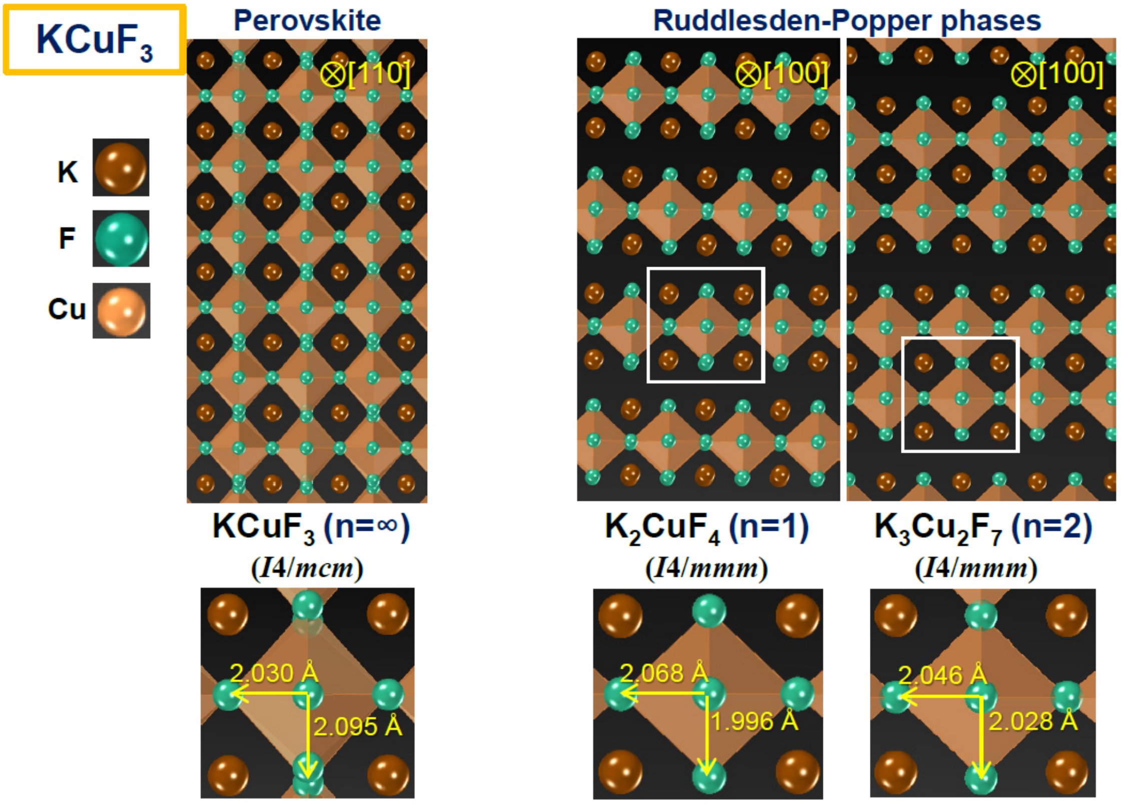

Supplementary Fig. 32 Crystal structures of K–Cu RP fluorides along with the perovskite counterpart. No significant *z*-axis elongation of F-anion octahedra in each phase is observed.

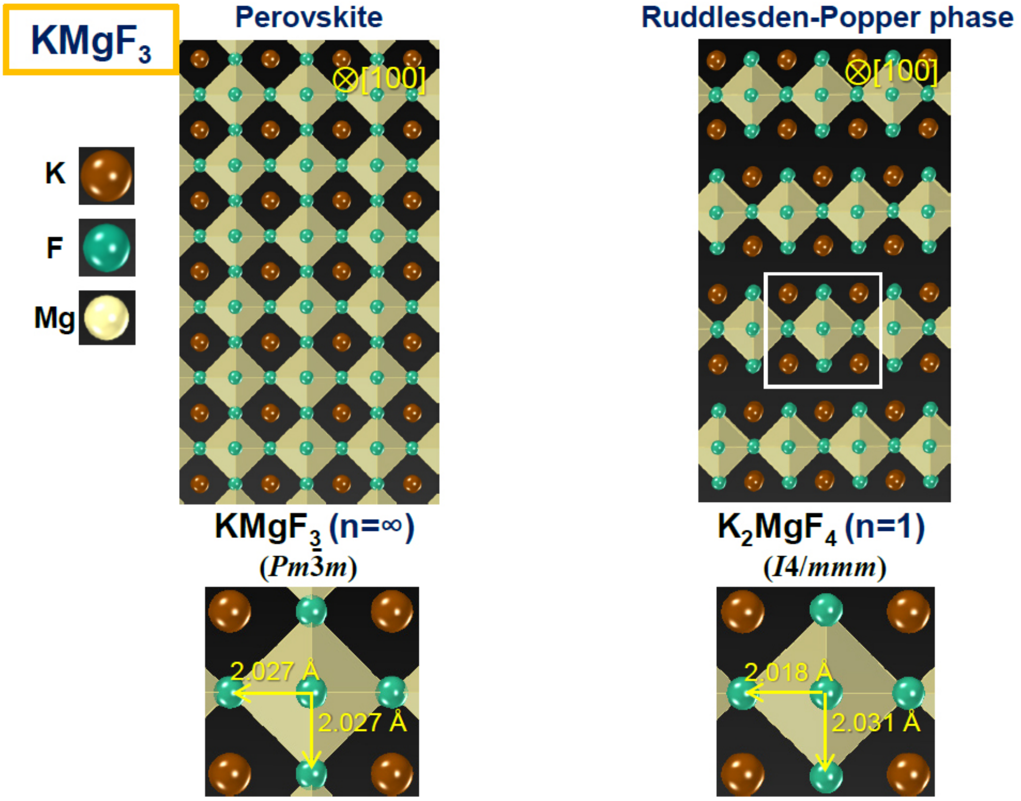

Supplementary Fig. 33 Crystal structures of K_2MgF_4 and its perovskite counterpart. No substantial *z*-axis elongation of F-anion octahedra is observed.

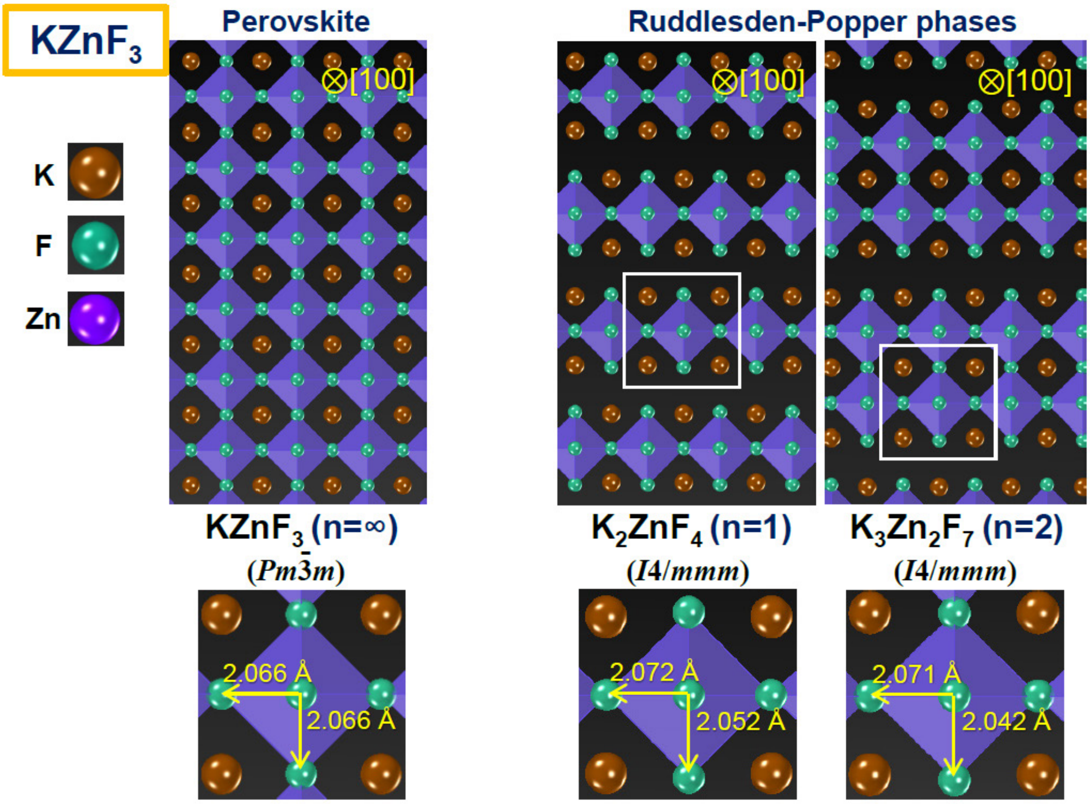

Supplementary Fig. 34 Crystal structures of K–Zn RP fluorides along with the perovskite counterpart. No significant *z*-axis elongation of F-anion octahedra in each phase is observed.

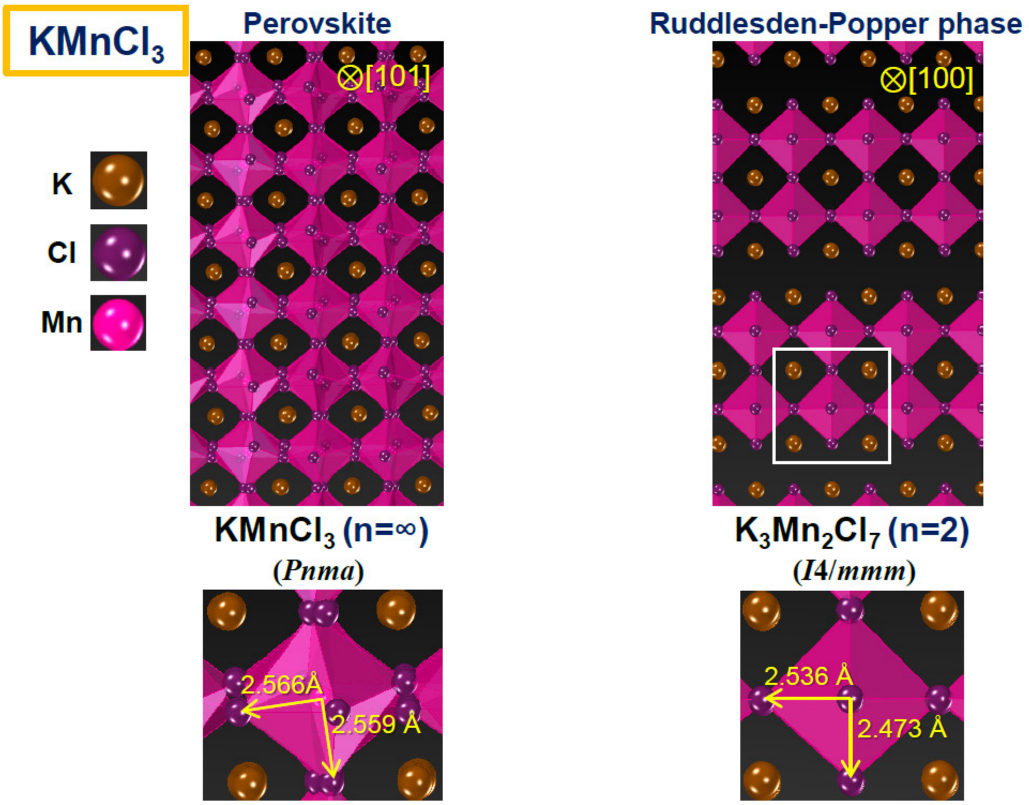

Supplementary Fig. 35 Crystal structures of $K_3Mn_2Cl_7$ and its perovskite counterpart. No substantial *z*-axis elongation of Cl-anion octahedra is observed.

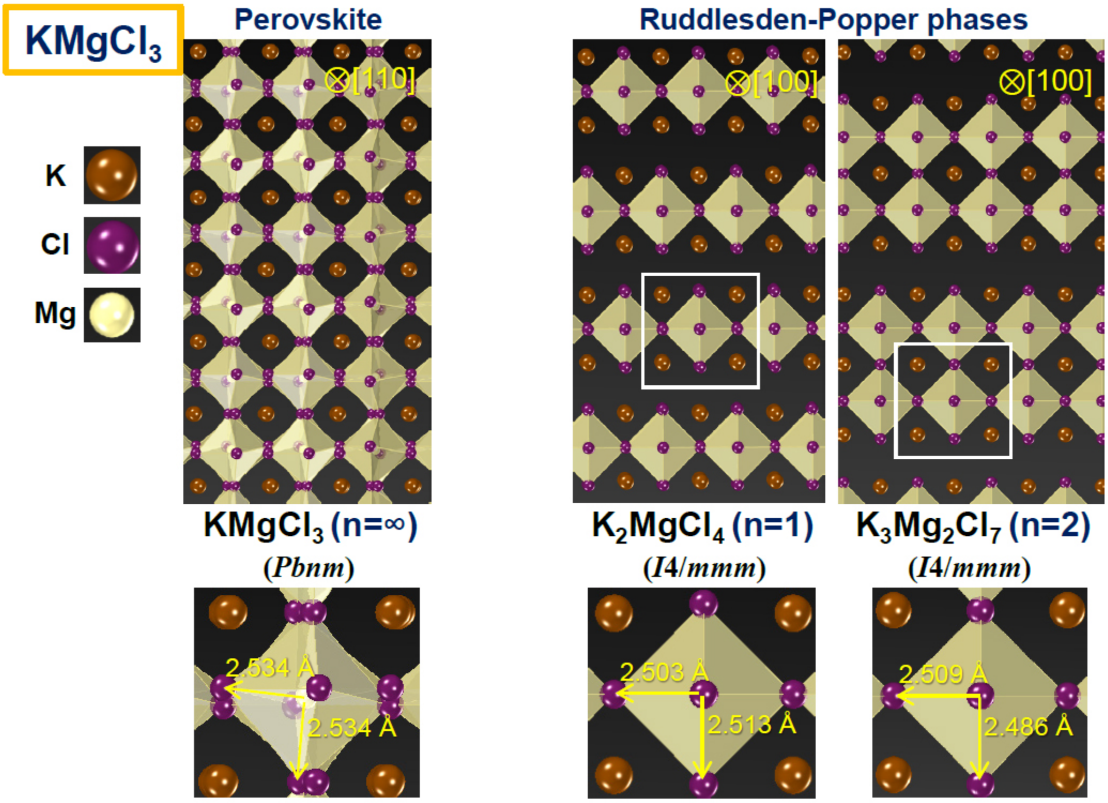

Supplementary Fig. 36 Crystal structures of K−Mg RP chlorides along with the perovskite counterpart. No significant *z*-axis elongation of Cl-anion octahedra in each phase is observed.

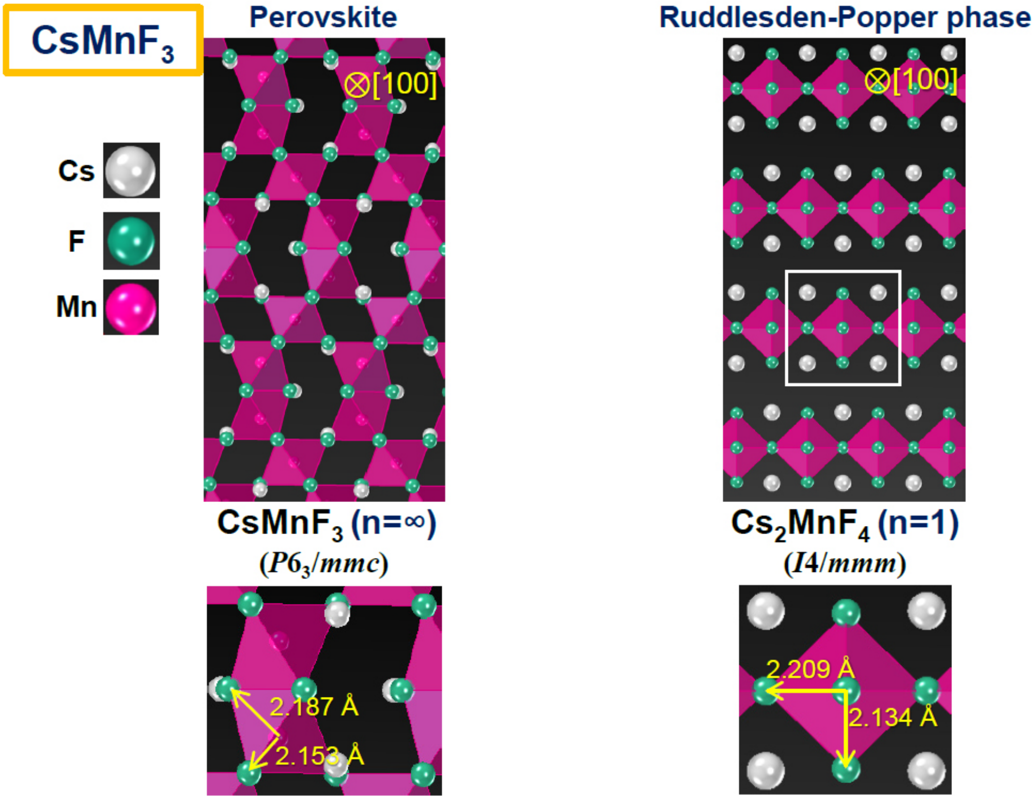

Supplementary Fig. 37 Crystal structures of Cs_2MnF_4 and its perovskite counterpart. No substantial *z*-axis elongation of F-anion octahedra is observed.

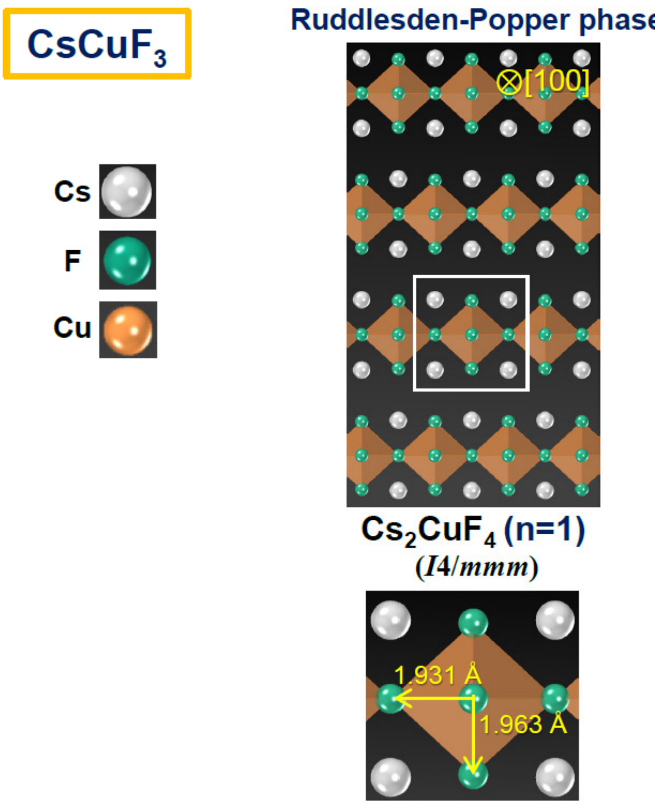

Supplementary Fig. 38 Crystal structure of Cs_2CuF_4. No significant *z*-axis elongation of F-anion octahedra in each phase is observed.

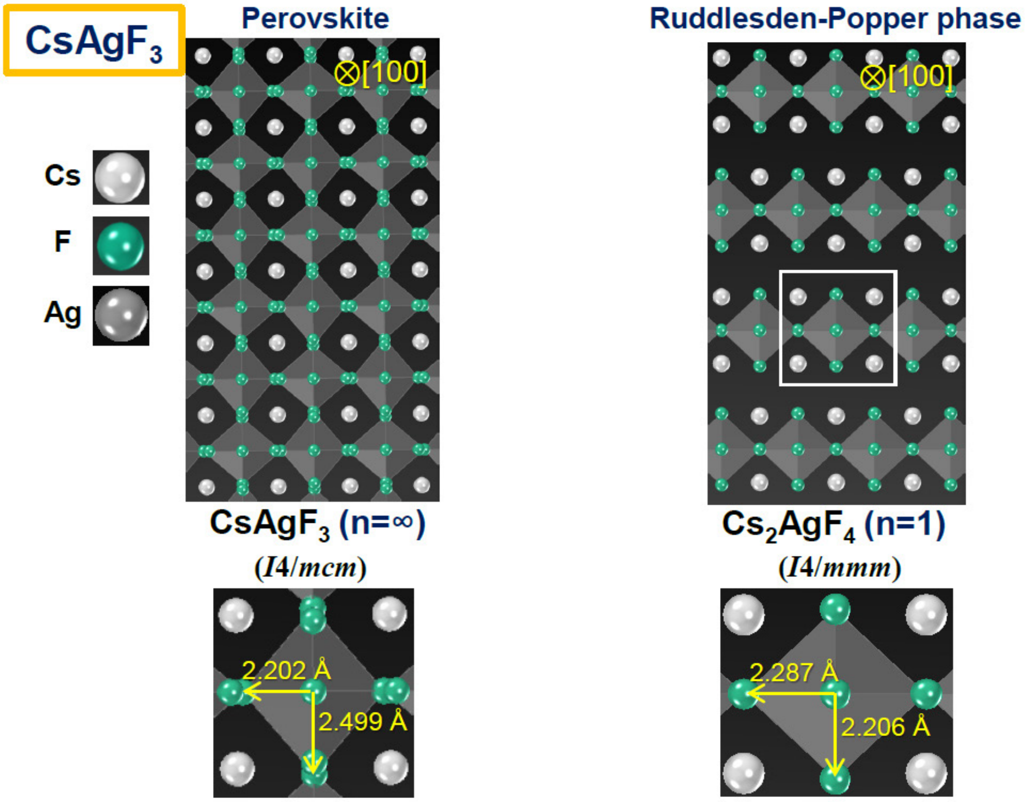

Supplementary Fig. 39 Crystal structures of Cs_2AgF_4 and its perovskite counterpart. No substantial *z*-axis elongation of F-anion octahedra is observed.

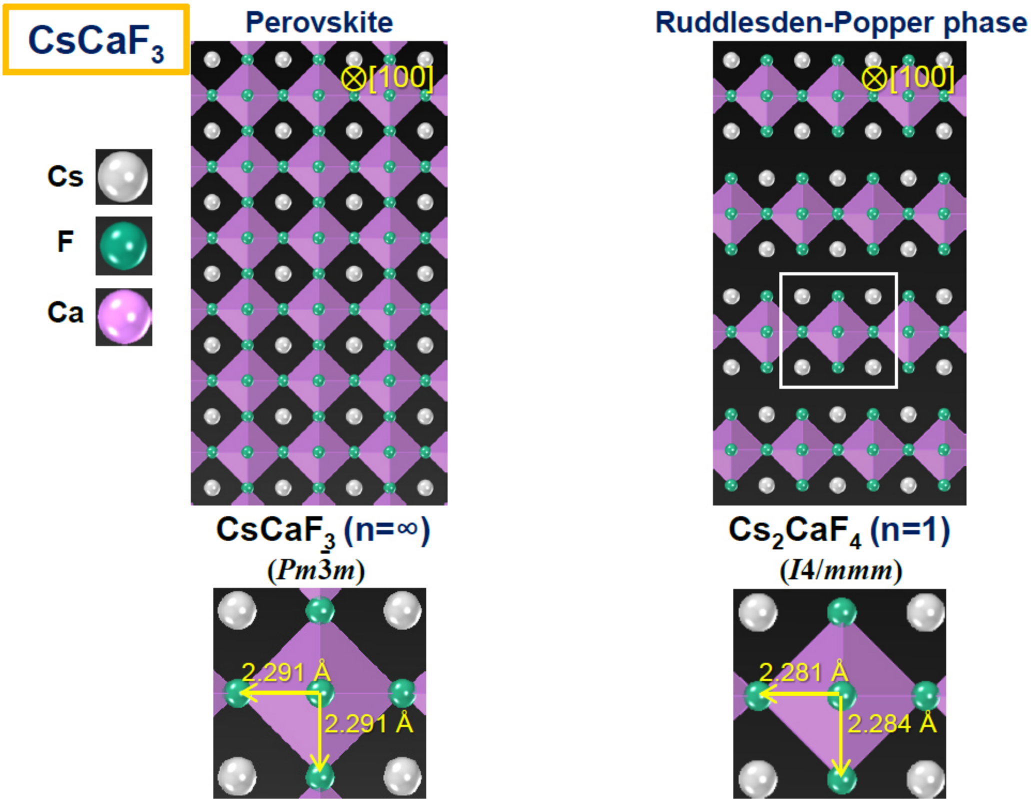

Supplementary Fig. 40 Crystal structures of Cs_2CaF_4 and its perovskite counterpart. No significant *z*-axis elongation of F-anion octahedra in each phase is observed.

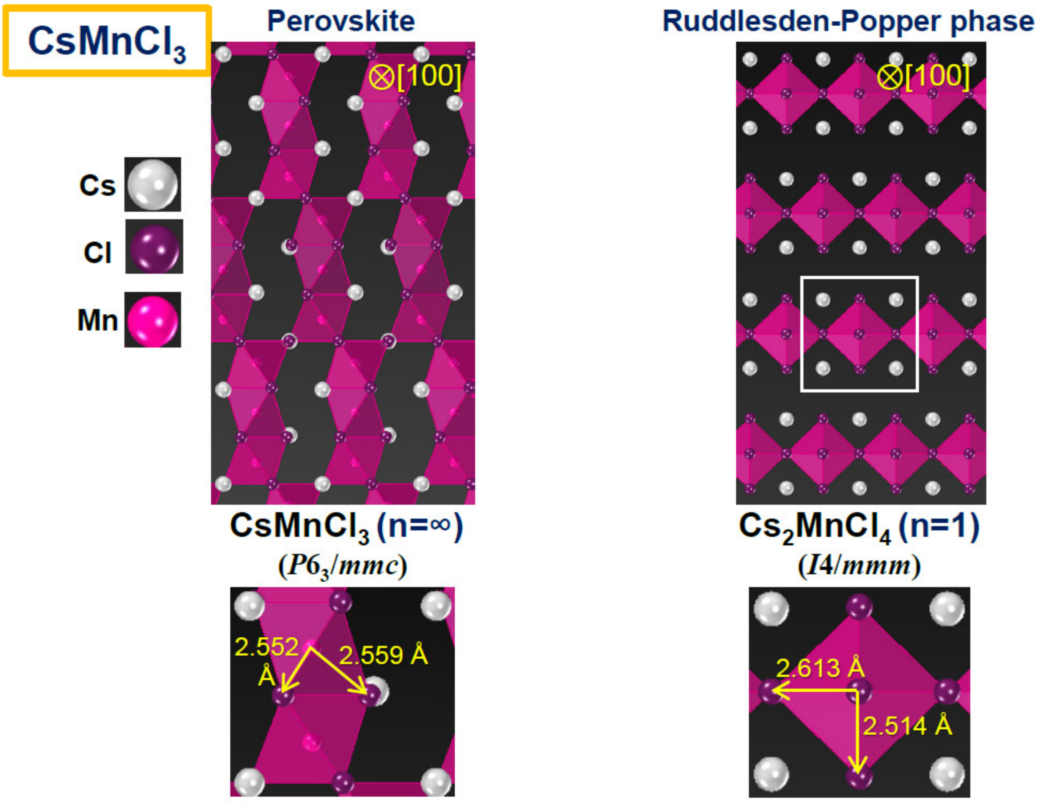

Supplementary Fig. 41 Crystal structures of Cs_2MnCl_4 and its perovskite counterpart. No substantial *z*-axis elongation of Cl-anion octahedra is observed.

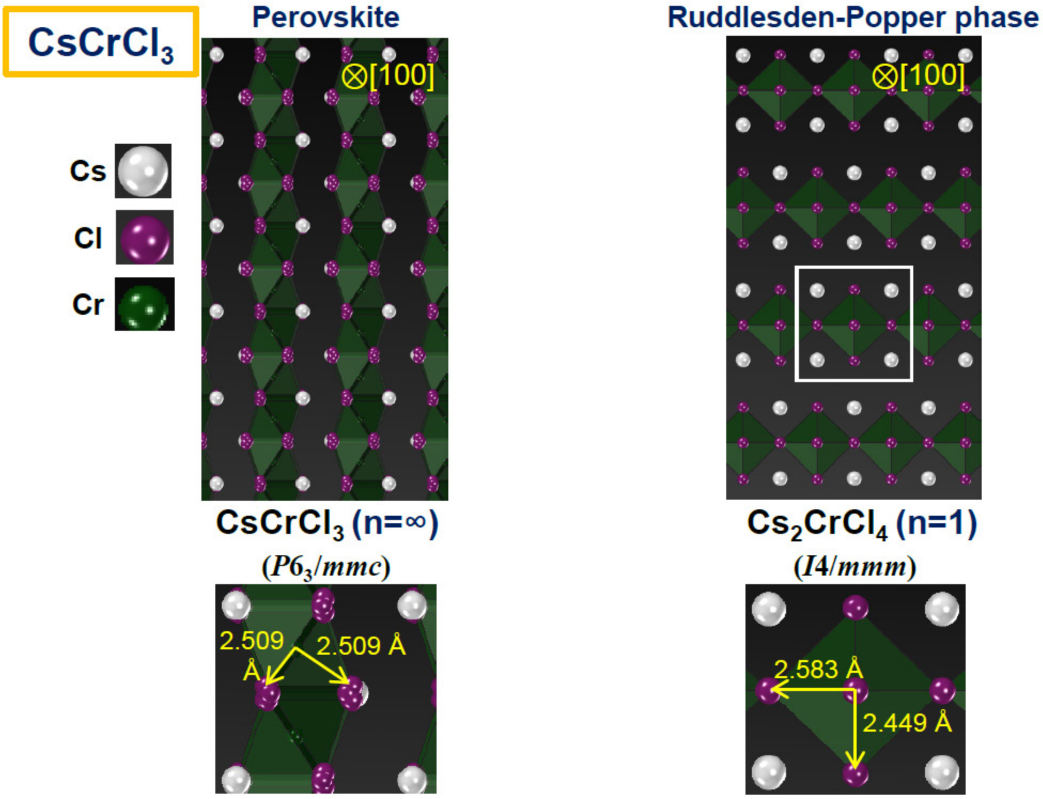

Supplementary Fig. 42 Crystal structures of Cs_2CrCl_4 and its perovskite counterpart. No significant *z*-axis elongation of Cl-anion octahedra in each phase is observed.

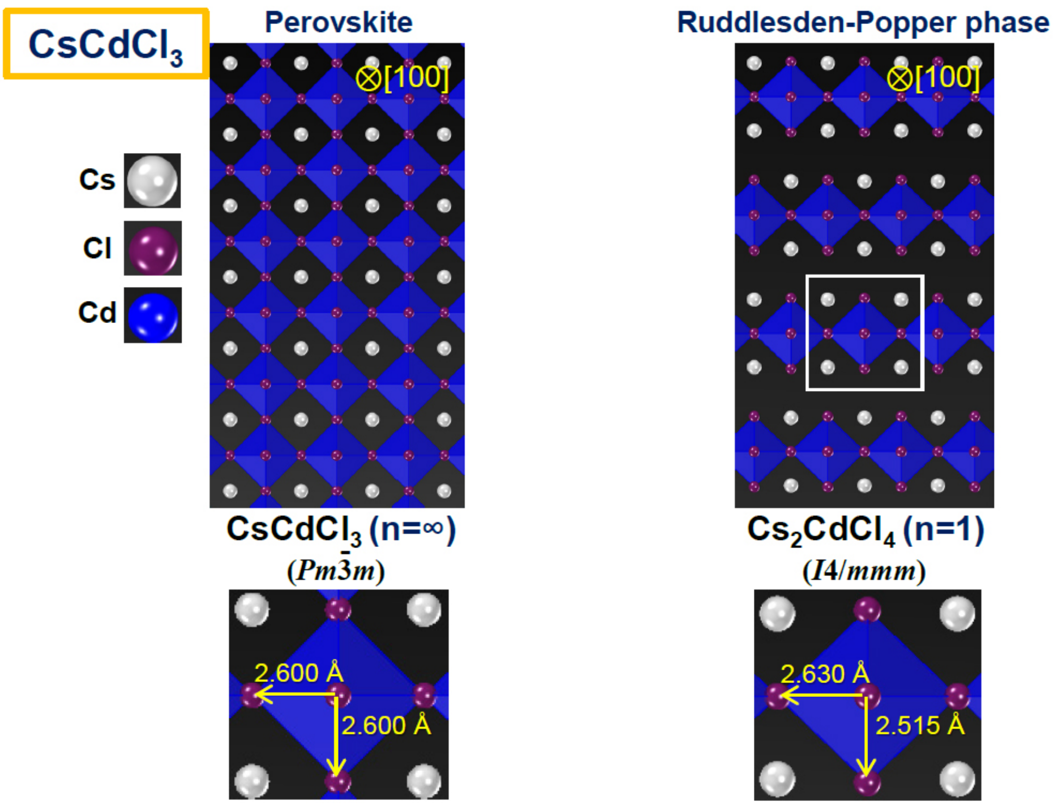

Supplementary Fig. 43 Crystal structures of Cs_2CdCl_4 and its perovskite counterpart. No substantial *z*-axis elongation of Cl-anion octahedra is observed.

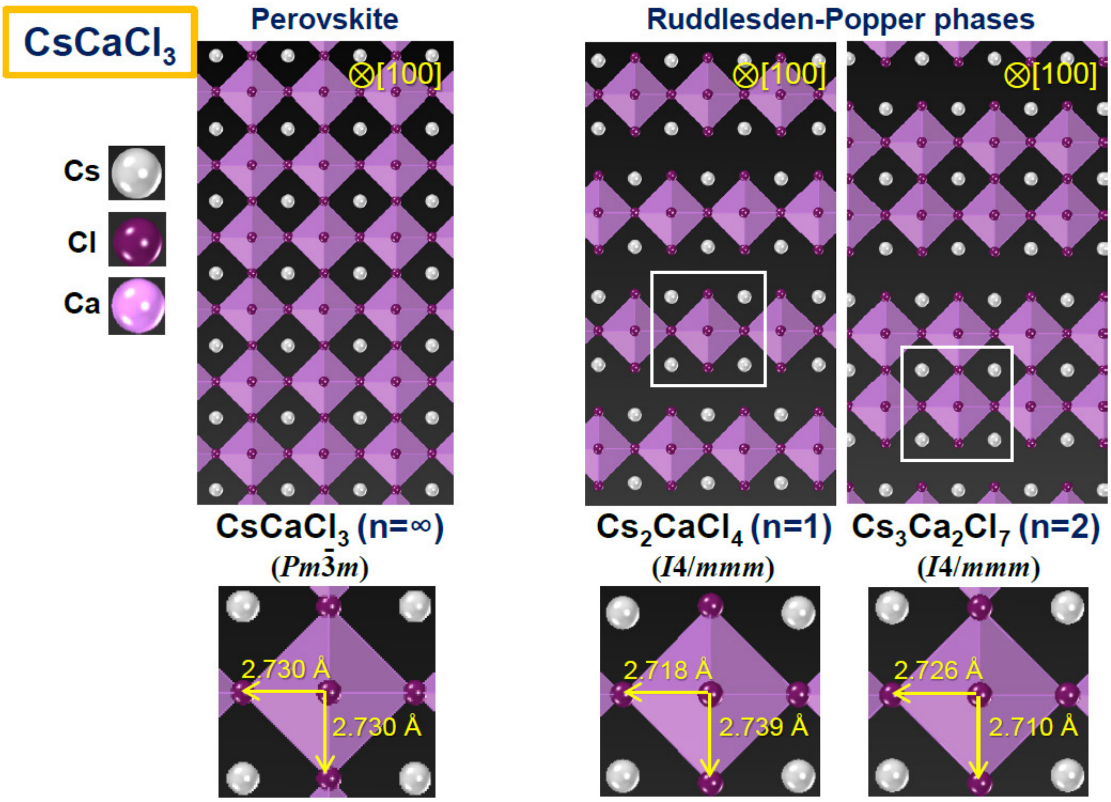

Supplementary Fig. 44 Crystal structures of Cs–Ca RP chlorides along with the perovskite counterpart. No significant *z*-axis elongation of Cl-anion octahedra in each phase is observed.

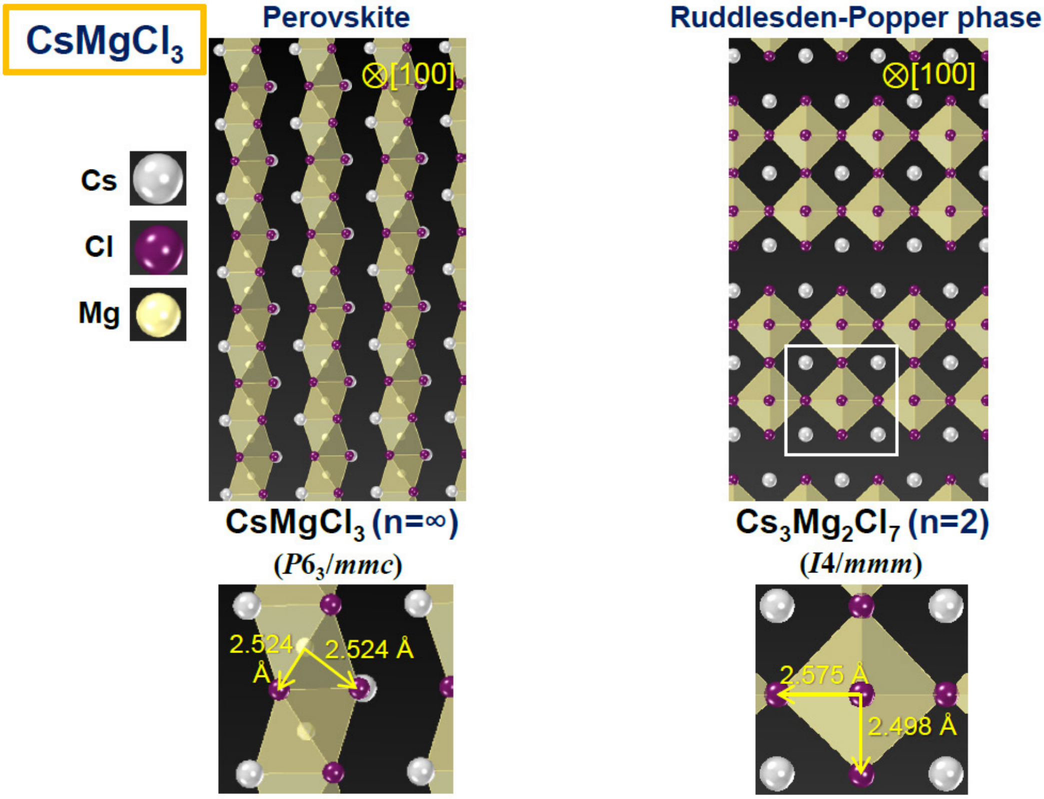

Supplementary Fig. 45 Crystal structures of $Cs_3Mg_2Cl_7$ and its perovskite counterpart. No substantial *z*-axis elongation of Cl-anion octahedra is observed.

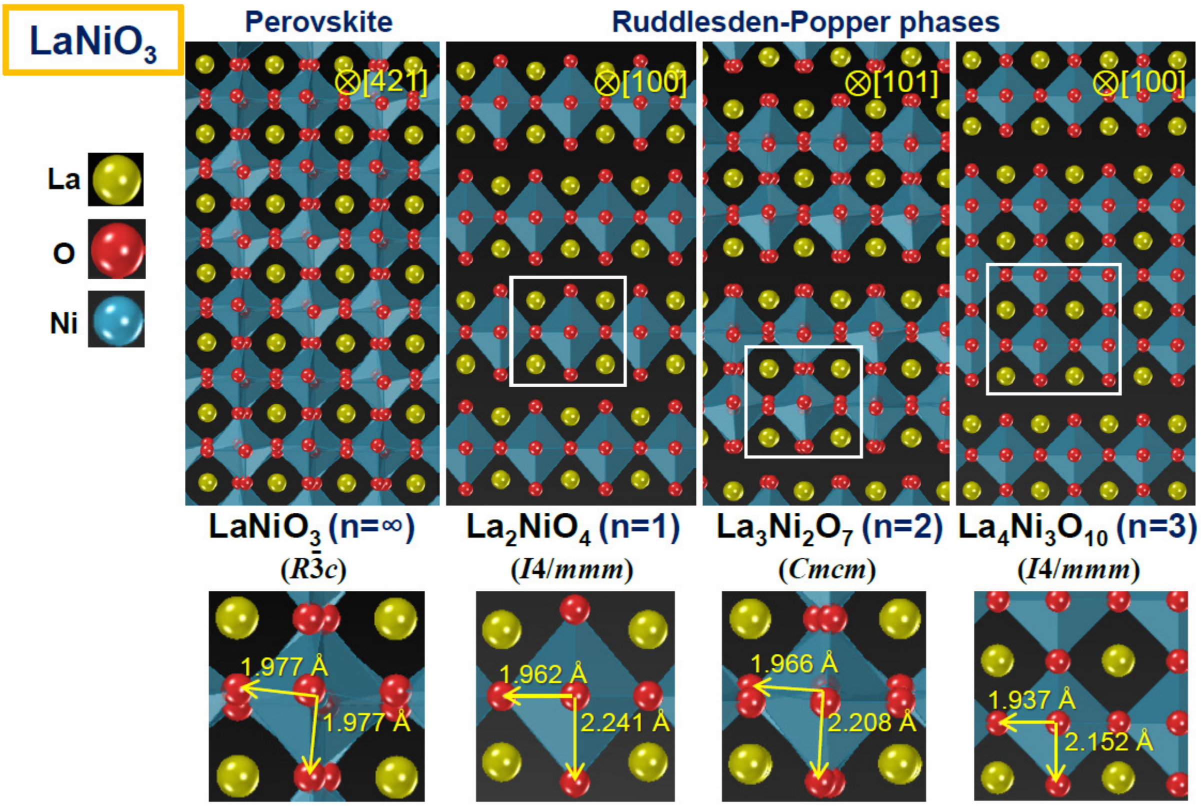

Supplementary Fig. 46 Crystal structures of La–Ni RP oxides along with the perovskite counterpart. More than 11% *z*-axis elongation of oxygen octahedra toward the shear plane in each phase is presented.

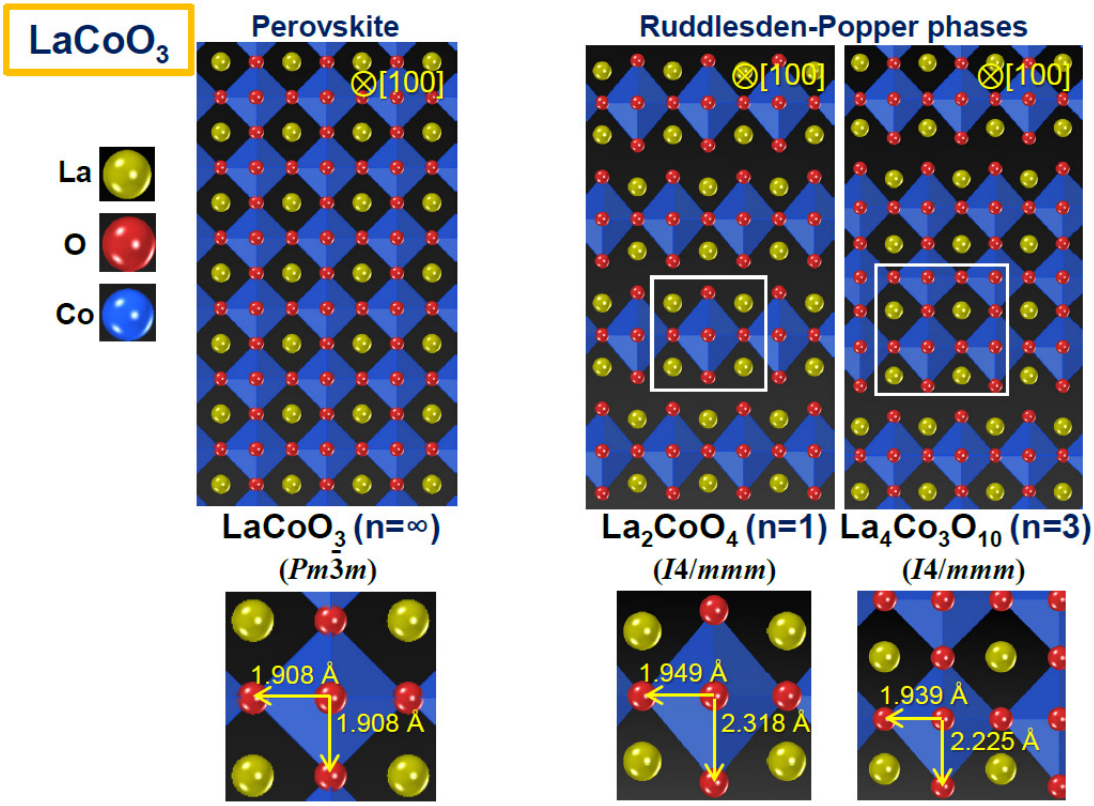

Supplementary Fig. 47 Crystal structures of La–Co RP oxides along with the perovskite counterpart. More than 14% *z*-axis elongation of oxygen octahedra toward the shear plane in each phase is noted.

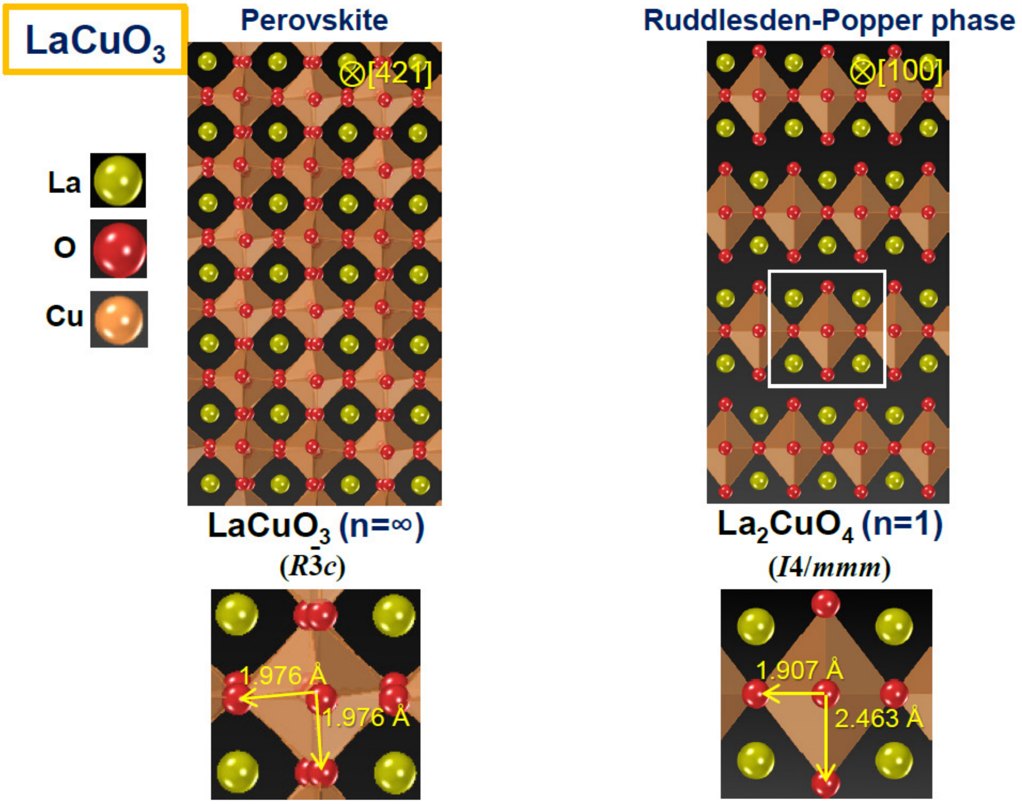

Supplementary Fig. 48 Crystal structures of a La–Cu RP oxide and the perovskite counterpart. More than 29% strong *z*-axis elongation of oxygen octahedra toward the shear plane is noted.

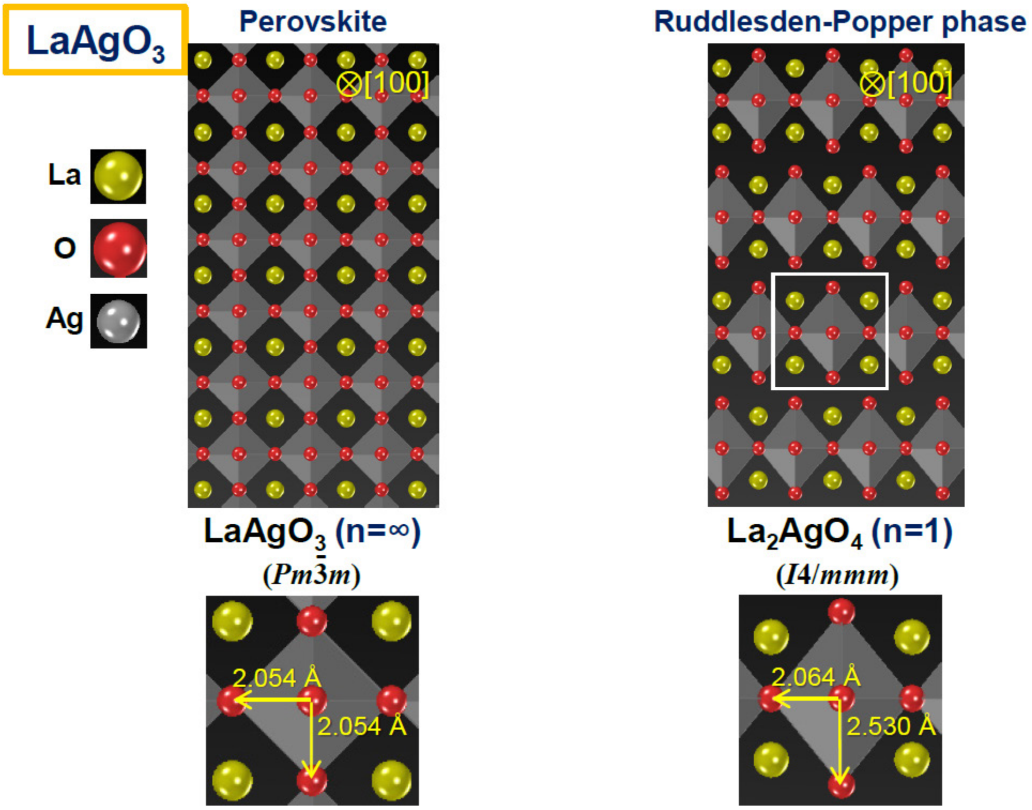

Supplementary Fig. 49 Crystal structures of a La–Ag RP oxide and the perovskite counterpart. More than 22% *z*-axis elongation of oxygen octahedra toward the shear plane is noted.

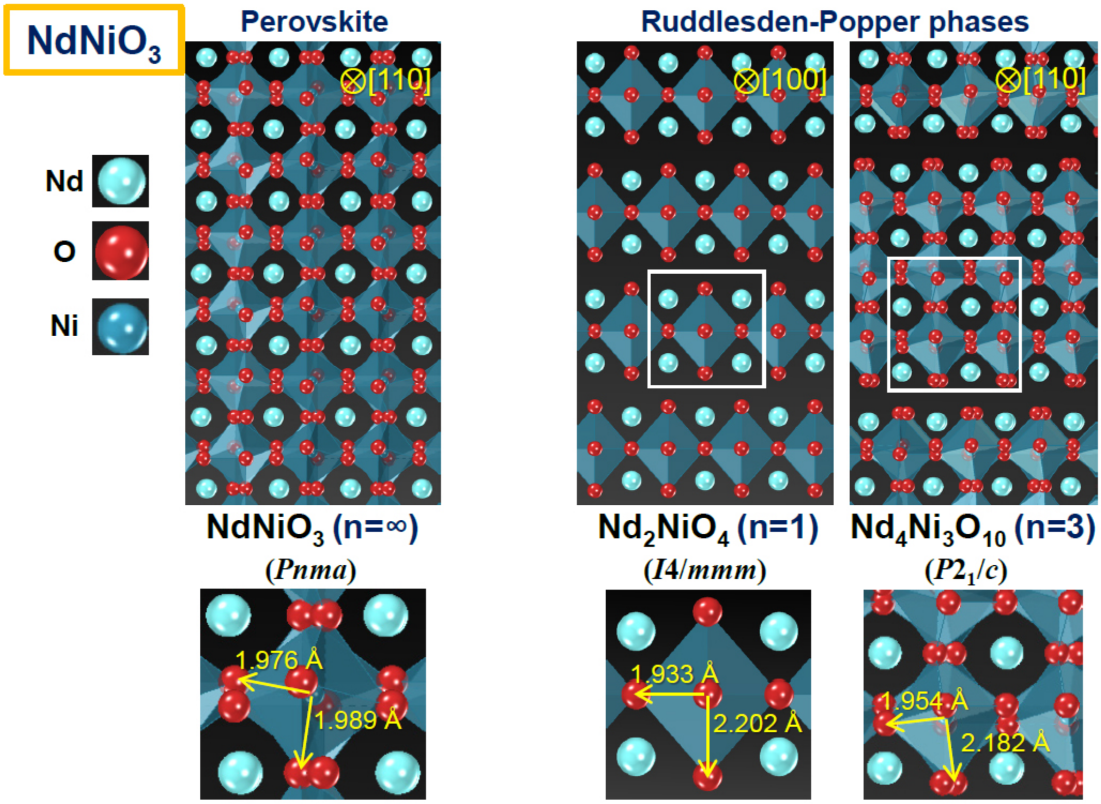

Supplementary Fig. 50 Crystal structures of Nd–Ni RP oxides along with the perovskite counterpart. More than 11% *z*-axis elongation of oxygen octahedra toward the shear plane in each phase is presented.

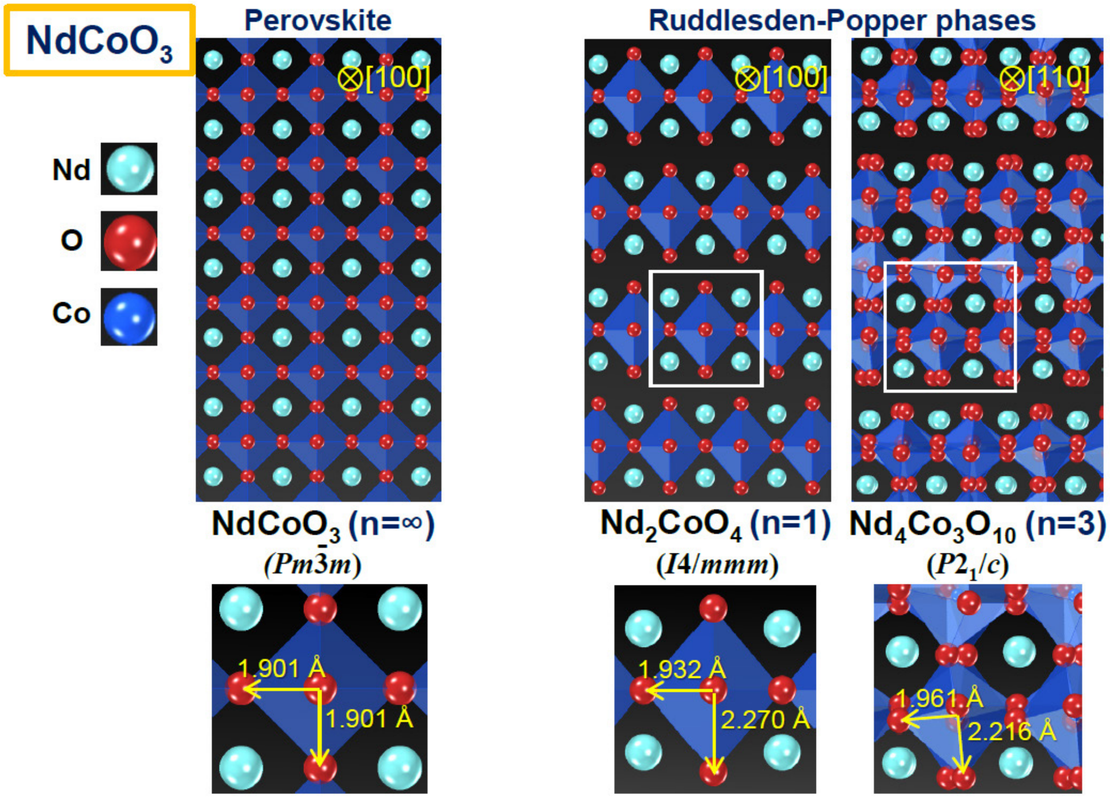

Supplementary Fig. 51 Crystal structures of Nd–Co RP oxides along with the perovskite counterpart. More than 13% *z*-axis elongation of oxygen octahedra toward the shear plane in each phase is noted.

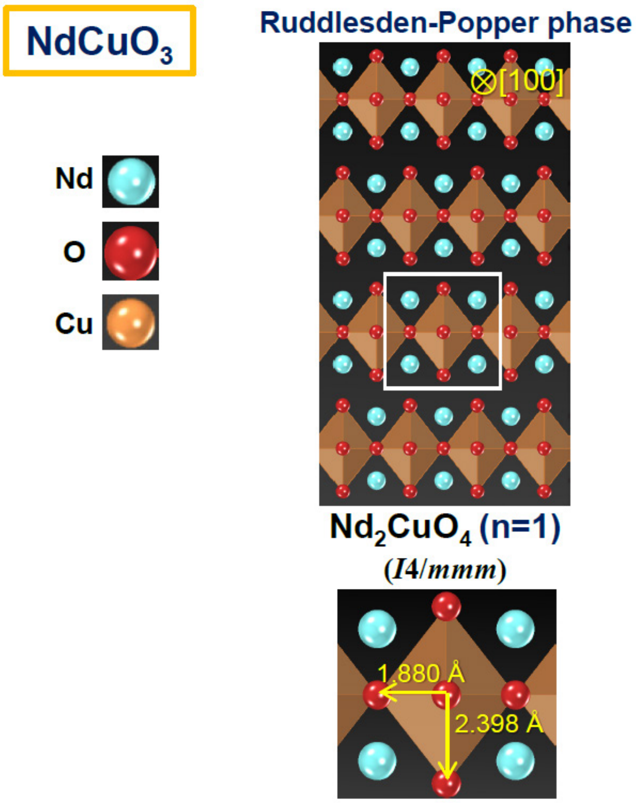

Supplementary Fig. 52 Crystal structure of a Nd–Cu RP oxide. More than 27% strong *z*-axis elongation of oxygen octahedra toward the shear plane is noted.

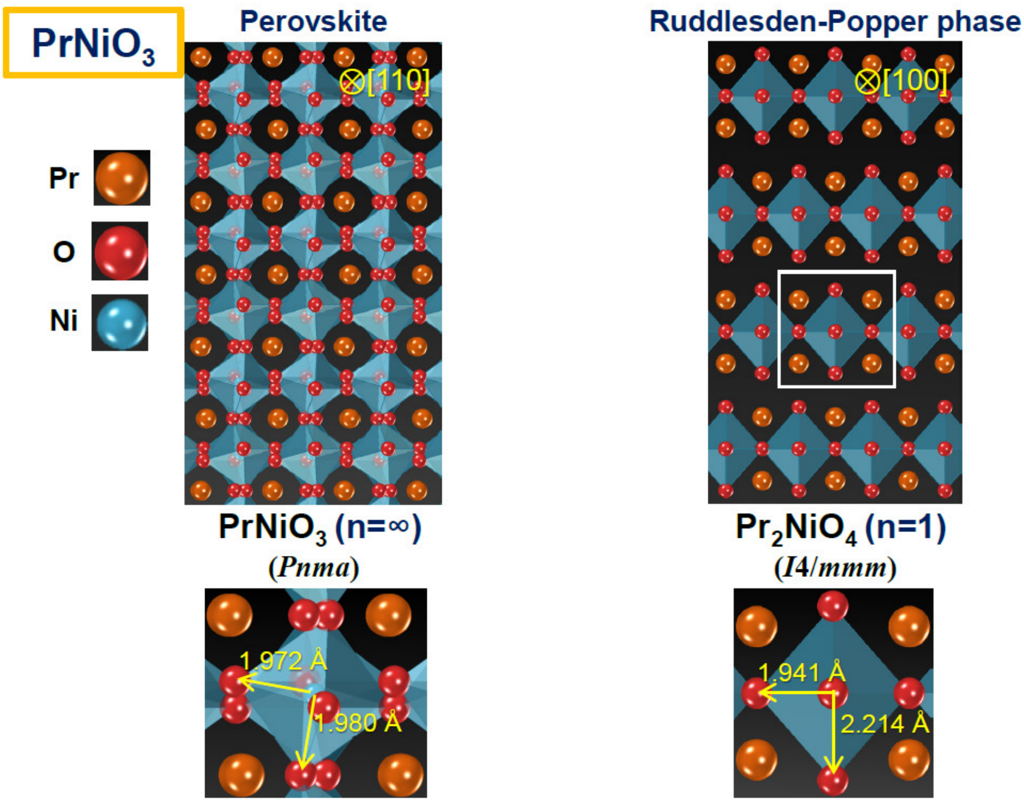

Supplementary Fig. 53 Crystal structures of a Pr–Ni RP oxide and the perovskite counterpart. More than 14% *z*-axis elongation of oxygen octahedra toward the shear plane is noted.

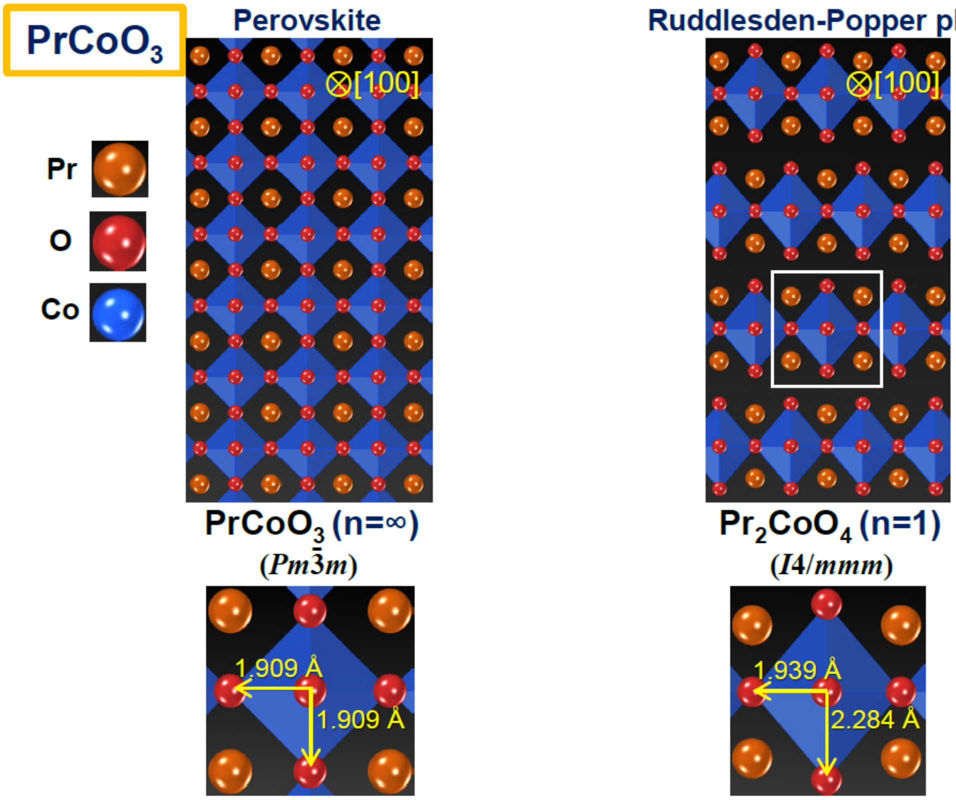

Supplementary Fig. 54 Crystal structures of a Pr–Co RP oxide and the perovskite counterpart. More than 17% strong *z*-axis elongation of oxygen octahedra toward the shear plane is noted.

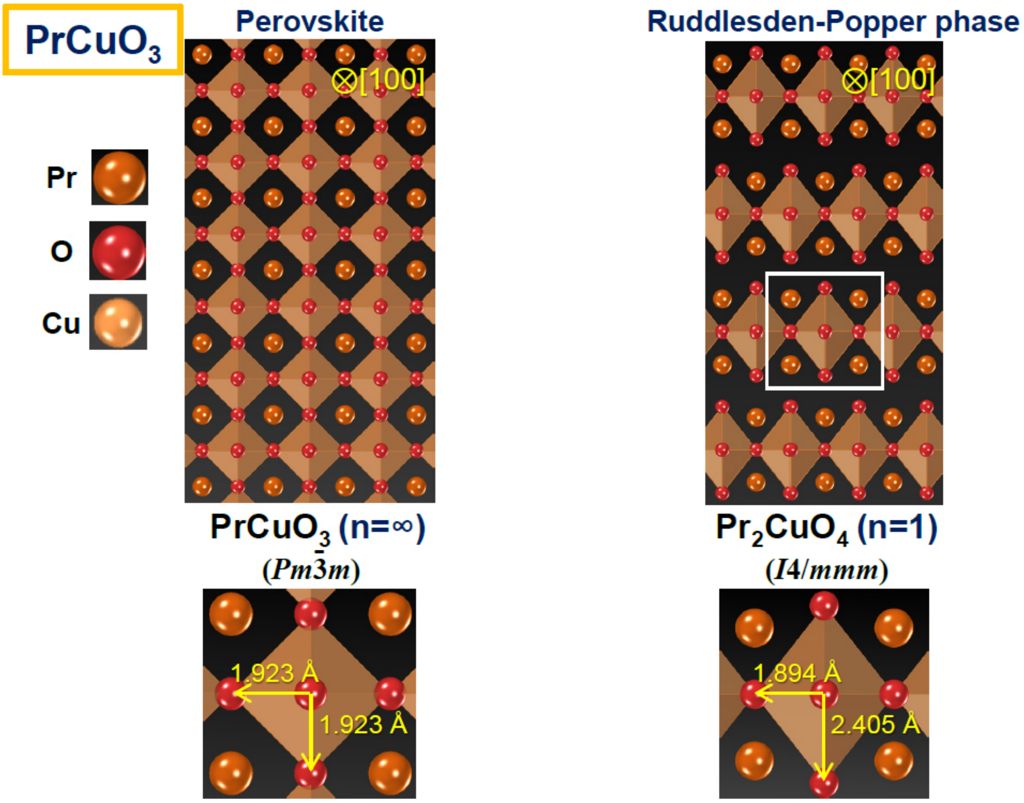

Supplementary Fig. 55 Crystal structures of a Pr–Cu RP oxide and the perovskite counterpart. More than 27% *z*-axis elongation of oxygen octahedra toward the shear plane is noted.

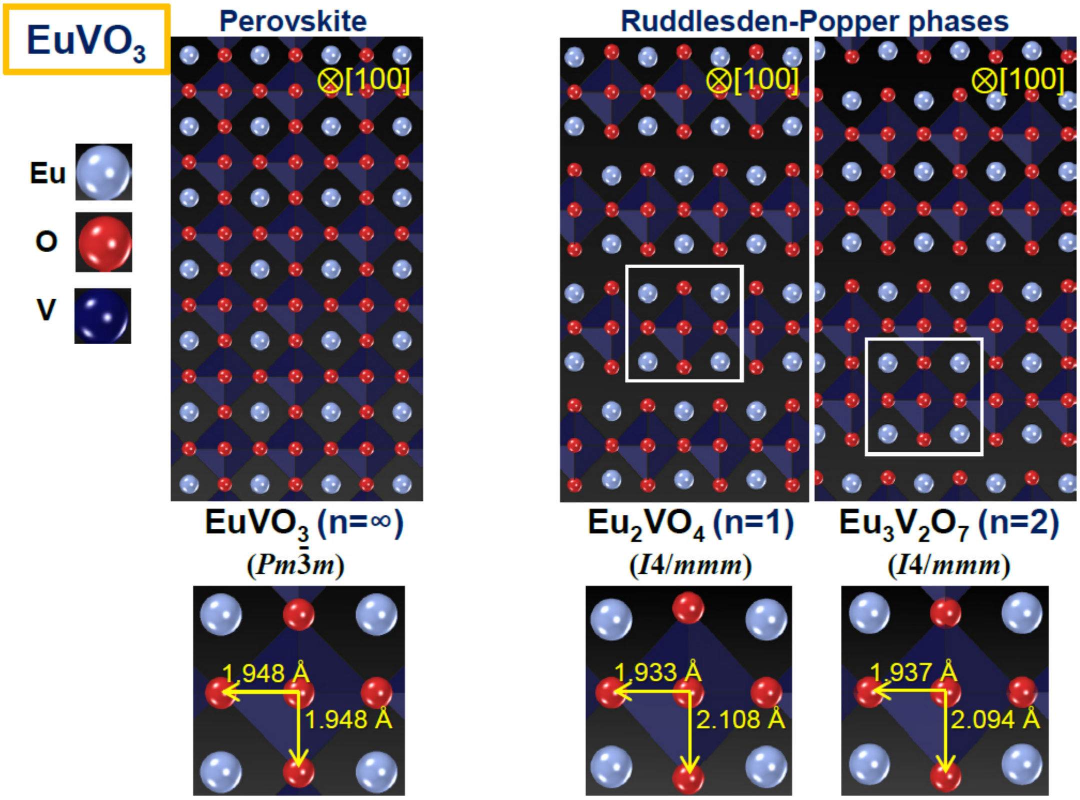

Supplementary Fig. 56 Crystal structures of Eu–V RP oxides along with the perovskite counterpart. More than 8% *z*-axis elongation of oxygen octahedra toward the shear plane in each phase is noted.

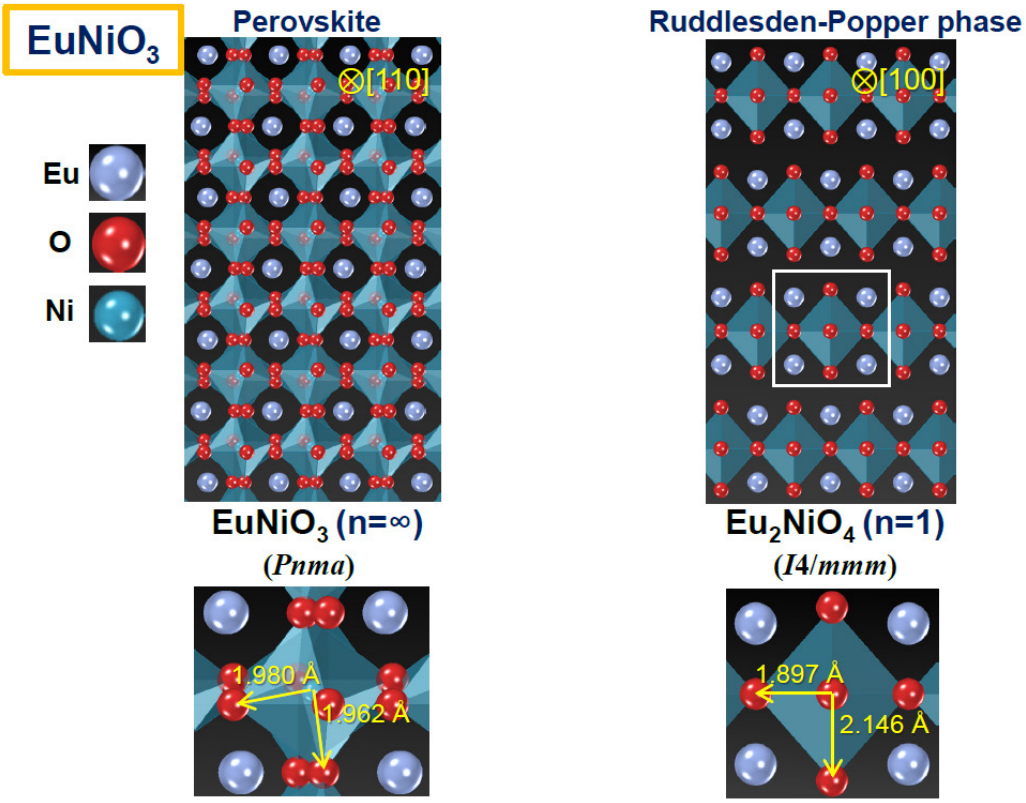

Supplementary Fig. 57 Crystal structures of a Eu–Ni RP oxide and the perovskite counterpart. More than 13% *z*-axis elongation of oxygen octahedra toward the shear plane is noted.

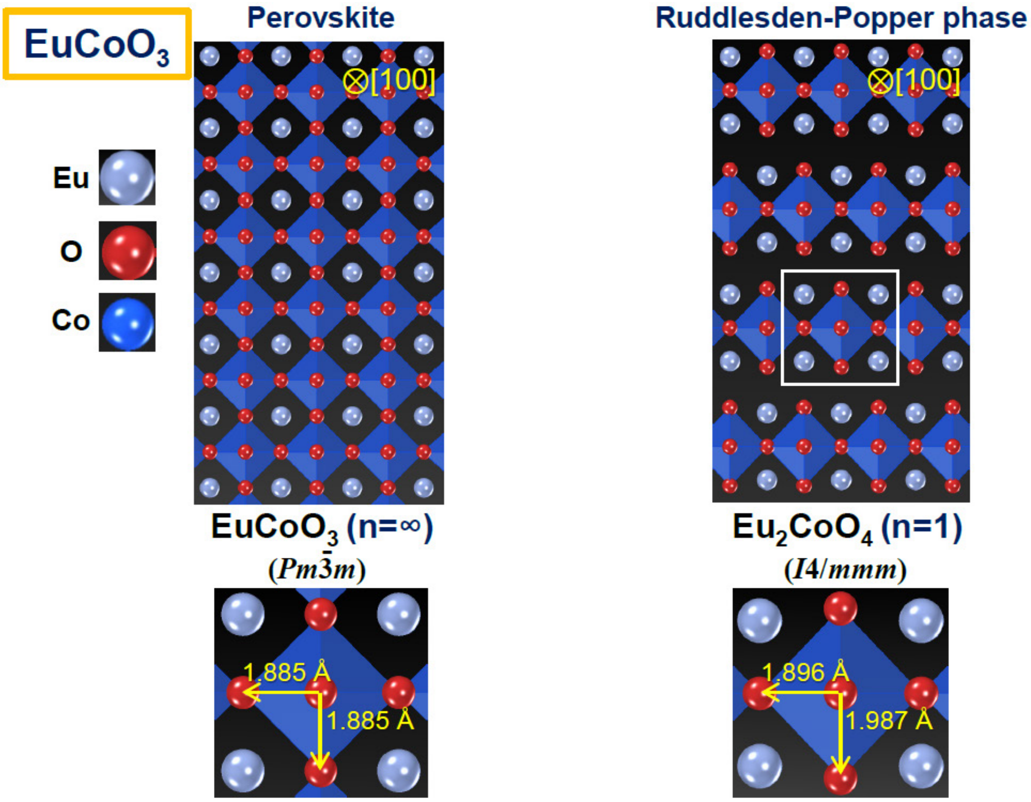

Supplementary Fig. 58 Crystal structures of a Eu–Co RP oxide and the perovskite counterpart. ~5% *z*-axis elongation of oxygen octahedra is presented.

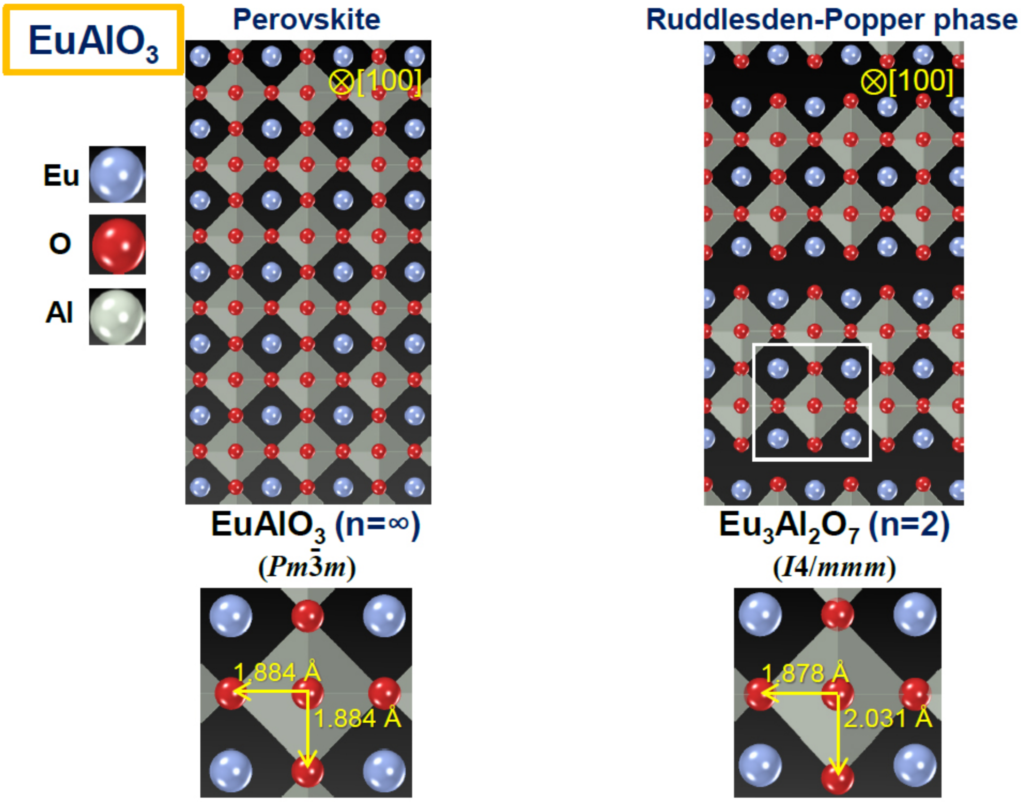

Supplementary Fig. 59 Crystal structures of a Eu–Al RP oxide and the perovskite counterpart. More than 8% *z*-axis elongation of oxygen octahedra toward the shear plane is noted.

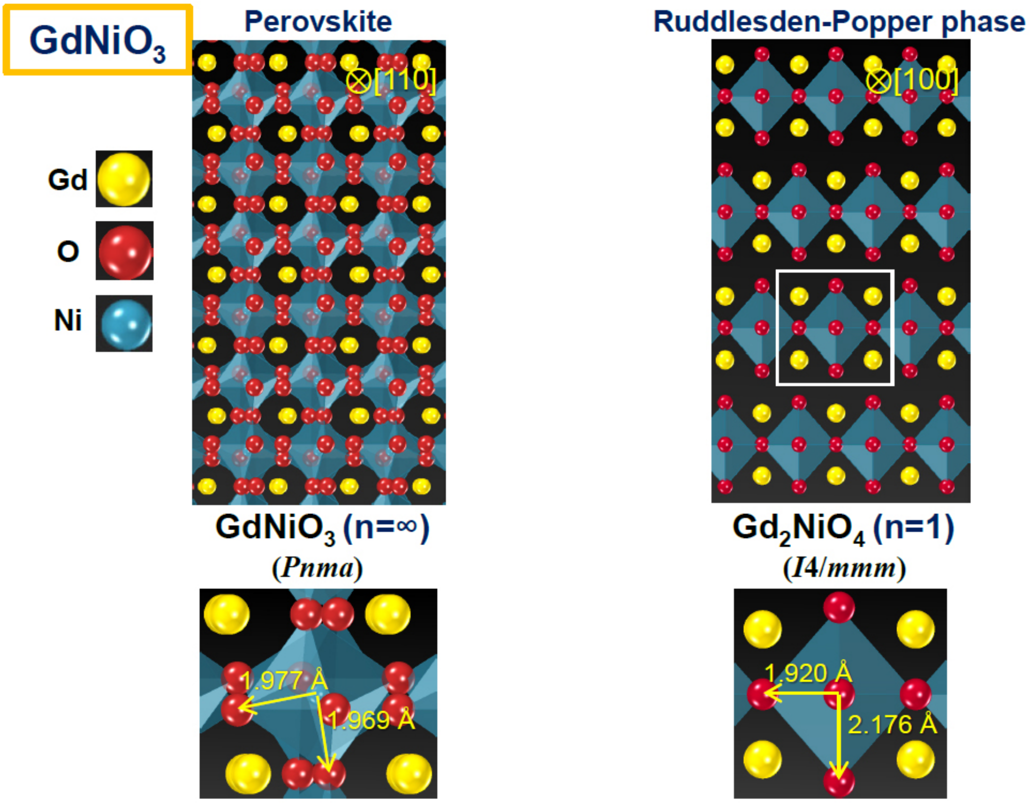

Supplementary Fig. 60 Crystal structures of a Gd–Ni RP oxide and the perovskite counterpart. More than 13% *z*-axis elongation of oxygen octahedra toward the shear plane is noted.

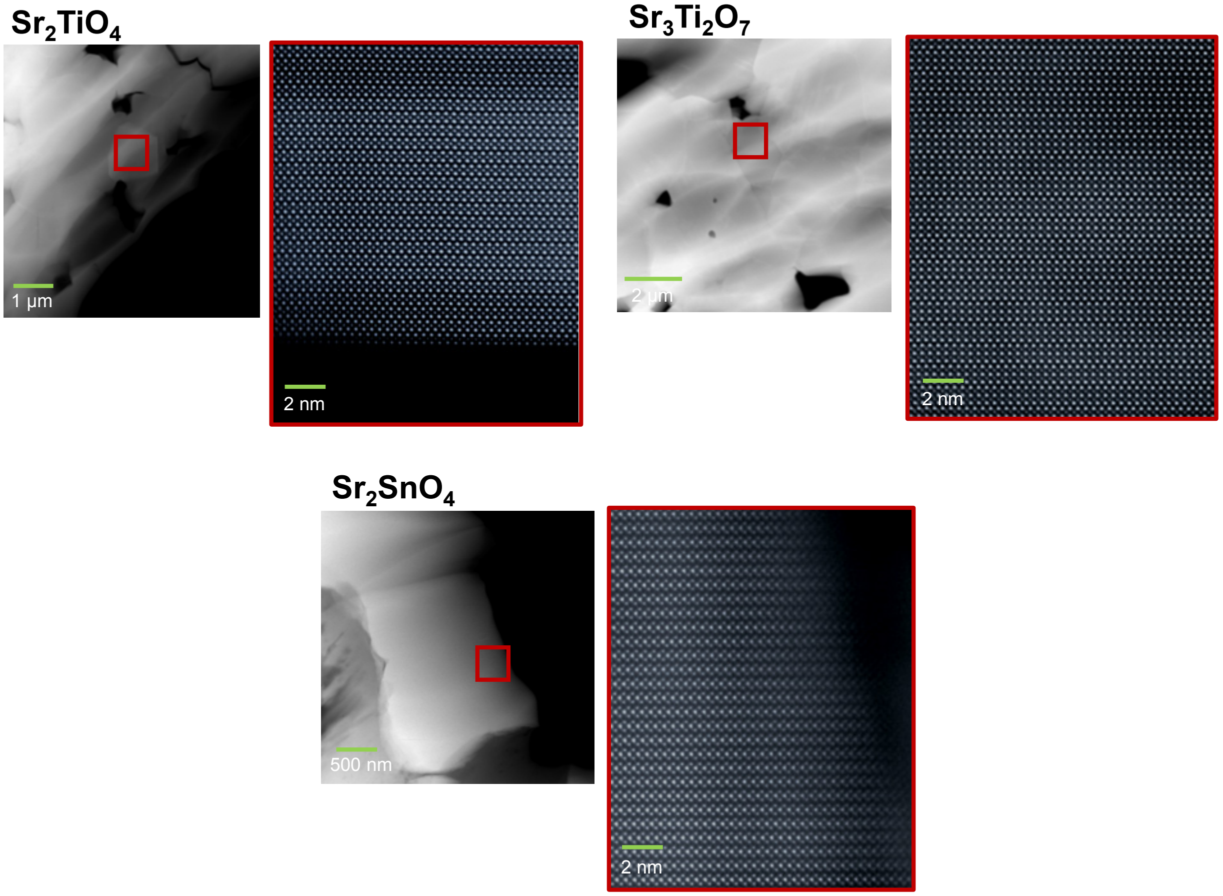

Supplementary Fig. 61 Additional HAADF images of Sr-based RP oxides. Pairs of low- and high-magnification images are provided to verify the successful phase synthesis of RP oxides, Sr_2TiO_4, $Sr_3Ti_2O_7$, and Sr_2SnO_4.

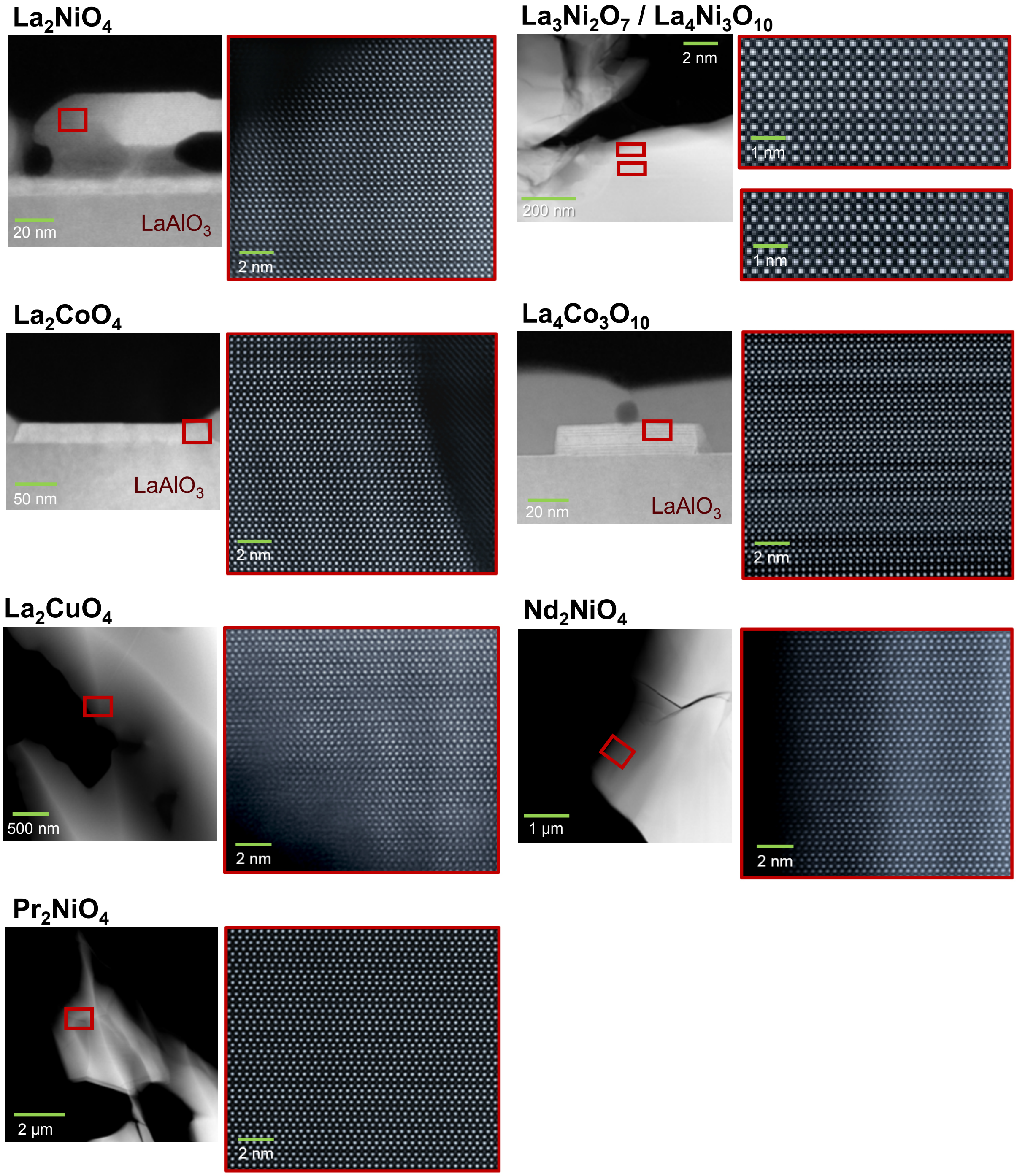

Supplementary Fig. 62. Additional HAADF images of lanthanide-based RP oxides. Pairs of low-, and high-magnification images are provided to verify the successful phase synthesis of the RP oxides. As denoted by red rectangles in the low-magnification image for $La_3Ni_2O_7$ and $La_4Ni_3O_{10}$ (in the form of $A_{n+1}B_nO_{3n+1}$, n = 2 and 3) the atomic-column resolved images were acquired from an identical sample, as the two distinct stacking sequences, n = 2 and 3, were simultaneously present in the same grain in the sample.

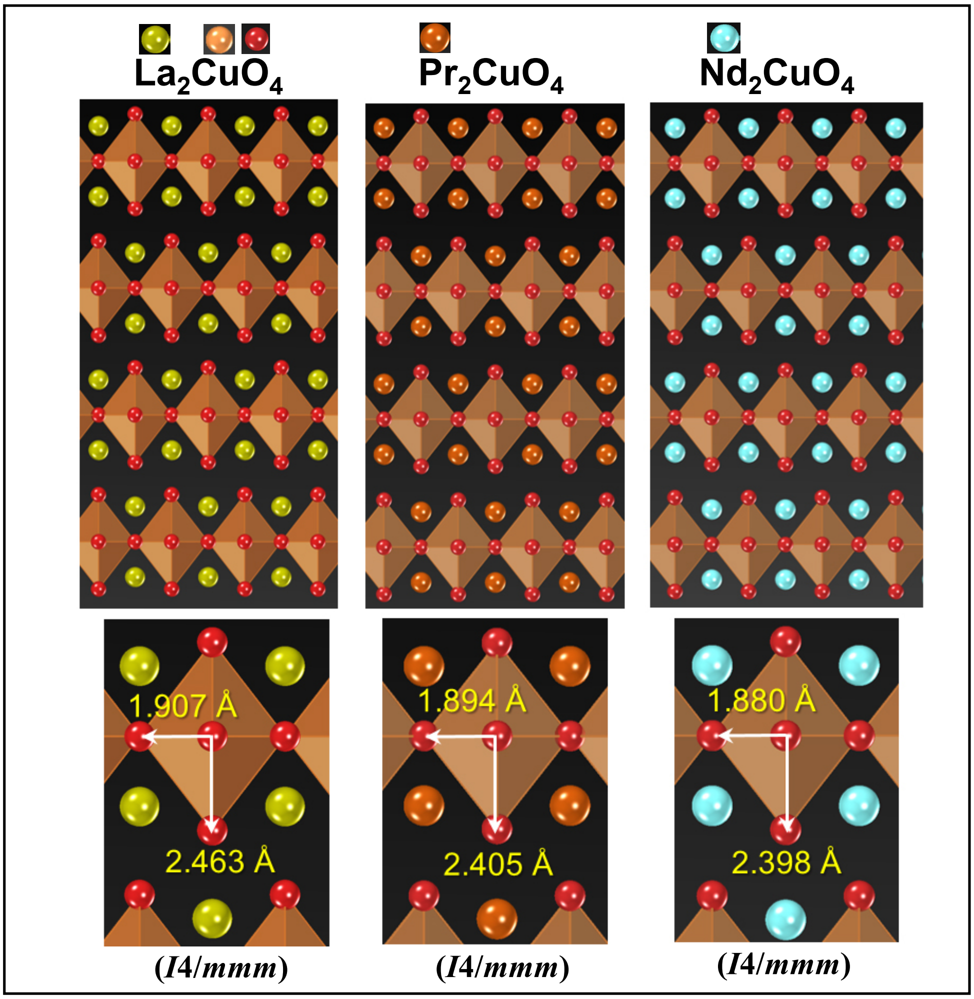

Supplementary Fig. 63 Comparison of crystal structures between La_2CuO_4, Pr_2CuO_4, and Nd_2CuO_4. Notably strong *z*-axis elongation of oxygen octahedra (>27%) is a common feature in these three Cu-based RP oxides.

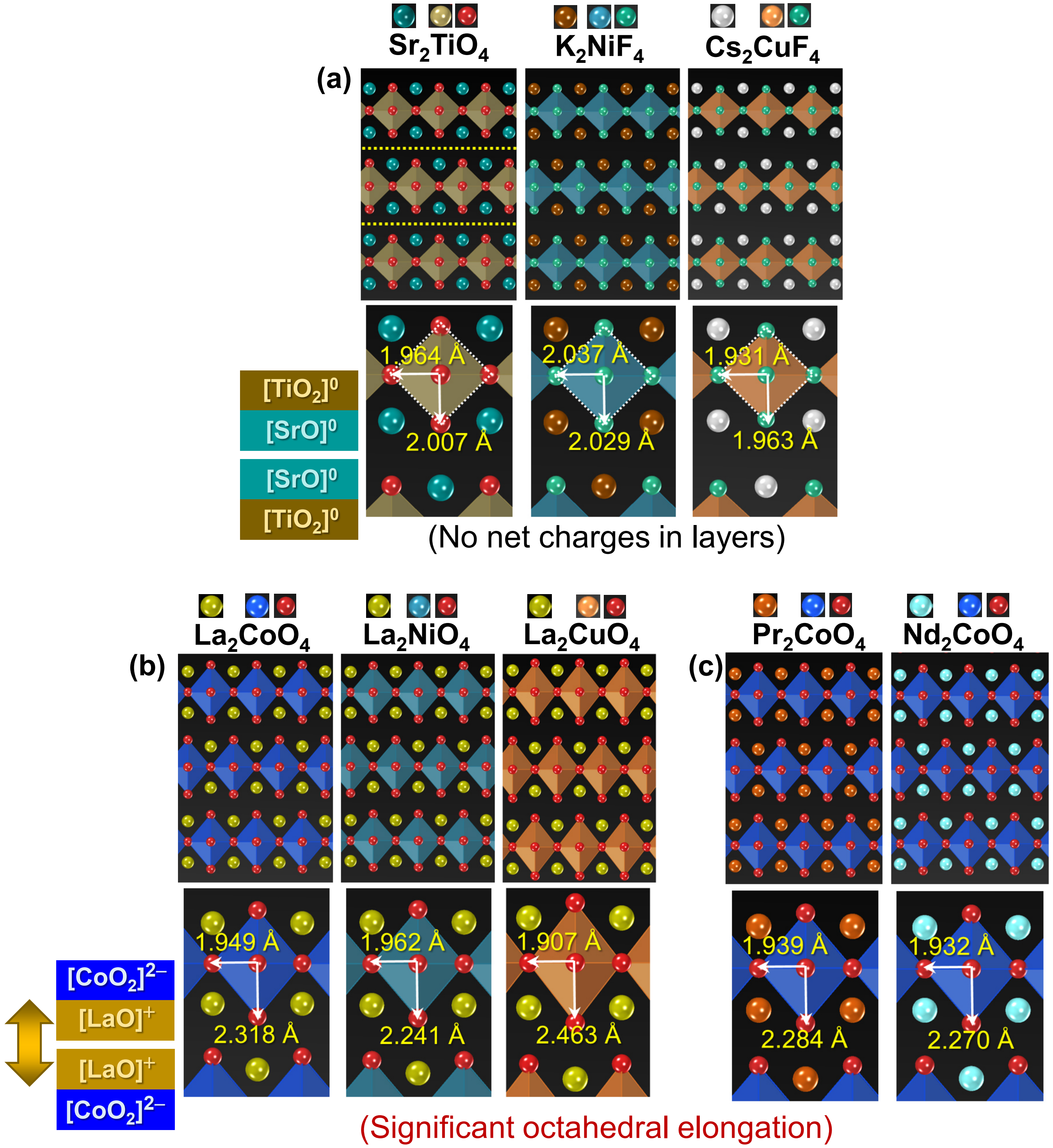

Supplementary Fig. 64 Structure comparisons between A_2BX_4-type RP phases. All the structures have a space group, *I*4/*mmm*. (**a**) Typical A_2BX_4 RP oxides and fluorides with no net charge in the [*A*X] sublayers are shown. Magnified illustrations demonstrate that there is no substantial elongation of anion octahedra. (**b**) La_2BO_4-type RP oxides (*B* = Co, Ni, Cu) are exemplified to clarify the impact of the positively charged $[LaO]^+$ sublayers on the substantial tetragonal distortion of oxygen octahedra. (**c**) In addition to La_2BO_4-type RP oxides, Pr- and Nd-based cobaltates, Pr_2CoO_4 and Nd_2CoO_4, are exemplified to show substantial *z*-axis octahedral elongation in a consistent manner.

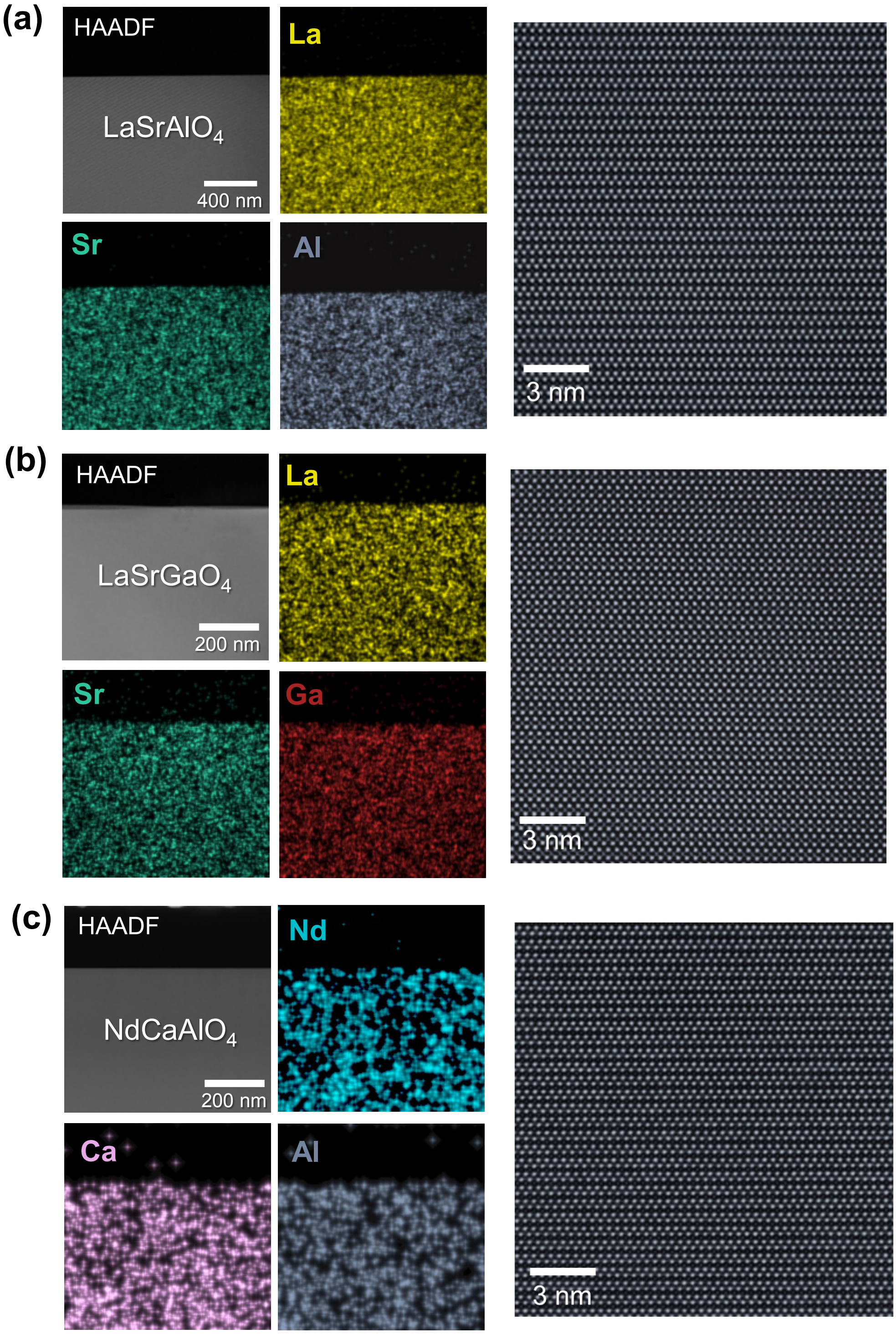

Supplementary Fig. 65 Composition maps and HAADF-STEM images of *A*-site solid-solution RP oxides. Three sets of chemical maps together with the STEM images are shown for (**a**) $LaSrAlO_4$, (**b**) $LaSrGaO_4$, and (**c**) $NdCaAlO_4$, demonstrating the homogeneous distribution of La–Sr and Nd–Ca.
